# AML/T cell interactomics uncover correlates of patient outcomes and the key role of ICAM1 in T cell killing of AML

**DOI:** 10.1038/s41375-024-02255-1

**Published:** 2024-05-09

**Authors:** Ece Canan Sayitoglu, Bogdan A. Luca, Allison Paige Boss, Benjamin Craig Thomas, Robert Arthur Freeborn, Molly Javier Uyeda, Pauline Ping Chen, Yusuke Nakauchi, Colin Waichler, Norman Lacayo, Rosa Bacchetta, Ravindra Majeti, Andrew J. Gentles, Alma-Martina Cepika, Maria Grazia Roncarolo

**Affiliations:** 1 Division of Hematology, Oncology, Stem Cell Transplantation, and Regenerative Medicine, Department of Pediatrics, Stanford University School of Medicine, Stanford, CA 94305 USA; 2 Department of Pathology, Stanford University School of Medicine, Stanford, CA 94305 USA; 3 Stanford Institute for Stem Cell Biology and Regenerative Medicine, Stanford University School of Medicine, Stanford, CA 94305 USA; 4 Stanford Cancer Institute, Stanford University School of Medicine, Stanford, CA 94305 USA; 5 Center for Definitive and Curative Medicine, Stanford University School of Medicine, Stanford, CA 94305 USA; 6 Division of Hematology, Department of Medicine, Stanford University School of Medicine, Stanford, CA 94305 USA

**Keywords:** Tumour immunology, Preclinical research, Tumour immunology

## Abstract

T cells are important for the control of acute myeloid leukemia (AML), a common and often deadly malignancy. We observed that some AML patient samples are resistant to killing by human-engineered cytotoxic CD4^+^ T cells. Single-cell RNA-seq of primary AML samples and CD4^+^ T cells before and after their interaction uncovered transcriptional programs that correlate with AML sensitivity or resistance to CD4^+^ T cell killing. Resistance-associated AML programs were enriched in AML patients with poor survival, and killing-resistant AML cells did not engage T cells in vitro. Killing-sensitive AML potently activated T cells before being killed, and upregulated *ICAM1*, a key component of the immune synapse with T cells. Without ICAM1, killing-sensitive AML became resistant to killing by primary ex vivo-isolated CD8^+^ T cells in vitro, and engineered CD4^+^ T cells in vitro and in vivo. While AML heterogeneity implies that multiple factors may determine their sensitivity to T cell killing, these data show that ICAM1 acts as an immune trigger, allowing T cell killing, and could play a role in AML patient survival in vivo.

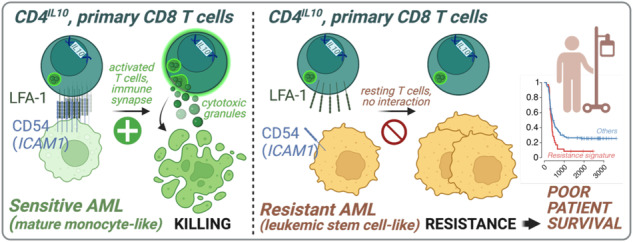

## Introduction

Acute myeloid leukemia (AML) affects ~20,000 new patients annually in the US. Many patients relapse after standard-of-care therapy [1], resulting in poor survival. Adult and pediatric AML patients have only ~30% and ~70% 5-year overall survival (OS), respectively [2, 3]. T cells play an important role in anti-tumor immunity and the long-term survival of AML patients. Elevated frequency of T cells in AML patient bone marrow correlates with increased survival [4]. Peripheral T cells in AML patients can exhibit dysregulated gene expression profiles, showing signs of activation but defects in immune synapse formation [5], which is required for tumor cell killing. Cytotoxic CD8^+^ T cells are considered critical for tumor control [6], but AML can cause CD8^+^ T cell dysfunction [7, 8]. T cell exhaustion and reduced CD4^+^ T helper (Th) cell activity have also been observed in AML [9].

While CD8^+^ T cells recognize AML antigens via HLA class I proteins, relapse in up to 40% of AML patients is driven by the loss of HLA class II [10, 11], which present antigens to CD4^+^ T cells. Antigen-activated CD4^+^ T cells secrete IL-2, IFN-γ, and TNF-α that activate CD8^+^ T cells, helping them kill tumor cells [12]. IFN-γ secreted by CD4^+^ T cells can restore HLA class II expression on AML [10, 11]. Finally, CD4^+^ T cells can also become cytotoxic and kill tumor cells directly [12], demonstrating their important role in the immune response to AML. Yet, the interaction between AML and CD4^+^ T cells, and its effect on AML patient survival, remains poorly understood.

Since primary human cytotoxic CD4^+^ T cells are rare, we opted to study AML-CD4^+^ T cell interaction using engineered cytotoxic CD4^+^ T cells, CD4^IL10^, generated by lentiviral overexpression of *IL10* gene in human CD4^+^ T cells [13–16]. Constitutive expression of *IL10* in CD4^+^ T cells counteracts exhaustion [17] and induces cytotoxic phenotype and functions [18]. CD4^IL10^ cells kill AML cell lines and primary AML samples in vitro [13–15, 19], and inhibit leukemia progression in vivo [14].

While most primary adult [14] and pediatric AML cells are killed by CD4^IL10^ cells, some are resistant to killing [15]. We previously showed that CD200 on resistant AML impairs the cytotoxicity of CD4^IL10^ cells [15], while others described the same effect on CD8^+^ T cells [20] and NK cells [21]. However, the partial inhibitory effect of CD200 was insufficient to explain AML resistance to T cell killing. To better understand AML-T cell interactions leading to AML killing, here we analyze killing-sensitive and -resistant primary AML samples before/after co-culture with CD4^IL10^ cells using single-cell RNA-sequencing (scRNA-seq). We show that resistance-related programs are found in less differentiated, stem cell-like AML, and in patients with worse survival. We also reveal that killing-sensitive, but not killing-resistant AML, successfully activate CD4^IL10^ cells in a process that involves *ICAM1*, which is required for the immune synapse formation with T cells. Finally, we demonstrate that ICAM1 expression is necessary for AML killing by both CD4^IL10^ cells and primary, ex vivo-isolated CD8^+^ T cells.

## Methods

### Primary human cells and cell lines

De-identified pediatric AML bone marrow aspirates were obtained from the Children’s Oncology Group (COG; CA, USA; protocol #AAML-18B2-Q, Table S1). Each sample contained ≥80% blasts (Table S1). Primary adult AML samples were provided by Dr. Ravindra Majeti (Stanford University Medical Center Institutional Review Board protocol # 6453). Primary human CD4^+^ and CD8^+^ T cells were isolated from de-identified healthy donor peripheral blood mononuclear cells (PBMC; Stanford Blood Center, CA, USA) via magnetic beads (Miltenyi Biotec, Germany). CD4^IL10^ cells were generated from healthy donor CD4^+^ cells and functionally tested as described [15, 16]. U937 (CRL-1593.2, ATCC, VA, USA), K562 (CCL-243, ATCC, VA, USA), and primary AML cells were cultured as before [15]. Cell lines were routinely tested for mycoplasma contamination.

### Killing and degranulation assays

CD4^IL10^ cells were co-cultured with target cells at a 1:1 effector to target (E:T) ratio for 3 days (killing) or 6 h (degranulation) as described [15], unless otherwise specified. Killing was calculated as % elimination efficiency (EE): [1 – (AML cell number cultured with T cells) / (AML cell number cultured alone)] × 100 [15]. When indicated, LFA-1 inhibitors (BIRT377, BMS688571, A286982 from Tocris Bioscience, UK; BMS587101 from MedChemExpress, NJ, USA) were added at 0.1, 1, and 10 μM, and EE was measured 24 h after co-culture. Killing assays with primary CD8^+^ T cells are described in Supplementary Methods.

### Flow cytometry

Data was acquired on BD LSR Fortessa and BD FACS Aria II (BD, NJ, USA), and analyzed using FlowJo 10.8 (BD, NJ, USA). Antibodies are listed in Table S2.

### CRISPR/Cas9-mediated genome editing

CRISPR/Cas9 knock-outs were performed as before [22] with minor optimizations (see Supplementary Methods).

### scRNA-seq

Four cryopreserved primary AML samples with pre-determined susceptibility to CD4^IL10^ killing were thawed and rested for 2 h at 37 ^o^C, then FACS-sorted for live cells and aliquoted for scRNA-seq analysis on day 0. Fresh resting CD4^IL10^ cells were also sorted for live cells and aliquoted for scRNA-seq analysis on day 0. Remaining AML and CD4^IL10^ cells were co-cultured for 24 h at a 1:1 ratio, then stained with anti-NGFR and anti-CD33 mAb to purify live CD4^IL10^ cells and AML cells, respectively, via FACS (CD4^IL10^ cells overexpress truncated *NGFR* as a marker gene [14]). After sorting, cells were re-adjusted to a 1:1 ratio to allow equal representation of AML and CD4^IL10^ cells and analyzed by scRNA-seq (24 h timepoint). This was repeated twice, each AML sample was co-cultured with T cells from two different donors. scRNA-seq was completed at Stanford Functional Genomics Facility (SFGF) using the Chromium Single Cell Gene Expression kit (10X Genomics, CA, USA) and Illumina Novaseq (Illumina Inc, CA, USA) sequencer following the manufacturer’s protocols.

### Single-cell analysis

Raw counts output by CellRanger [23] were loaded into Seurat v4.0.5 [24] according to the accompanying vignette; details and modifications from default settings are listed in Supplementary Methods.

### In vivo experiments

Described in Supplementary Methods.

### Statistical analysis

Performed in R 4.0.2 for scRNA-seq, and in GraphPad Prism 9.5.1 for other experiments. Statistical tests used (α at 0.05) are indicated in Figure legends. There was no assumption of normal distribution unless otherwise specified. For the estimation of variation, the median and interquartile range are shown.

## Results

### Single-cell interaction analysis of primary AML and CD4^IL10^ cells

**Fig. 1:**
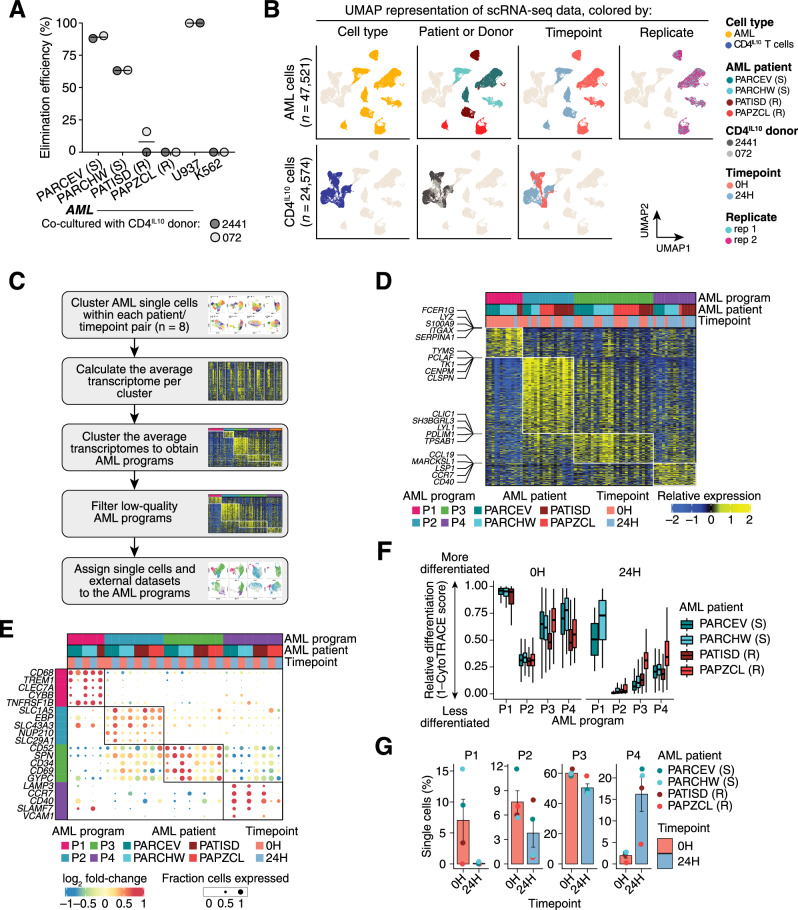
Longitudinal single-cell analysis of primary AML cell/CD4^IL10^ cell interactions identifies AML transcriptional programs. **A** Each aliquot of primary AML cells was co-cultured with CD4^IL10^ cells (*n* = 2) for 3 days in a 1:1 ratio, then enumerated using flow cytometry, and killing expressed as % elimination efficiency (“Methods” section). The X-axis indicates AML sample ID and their sensitivity (S) or resistance (R) to CD4^IL10^ cell killing. CD4^IL10^ killing-sensitive, monocytic myeloid leukemia cell line U937 was used as a positive control, while CD4^IL10^ killing-resistant, erythroleukemic K562 cell line was used as a negative control in each experiment. **B** UMAP representation of scRNA-seq data, showing AML (upper panel) and CD4^IL10^ single cells (lower panel). **C** Workflow depicting the derivation of AML programs (“Supplementary Methods” section). **D** Heatmap of the four AML transcriptional programs identified. Rows represent genes, columns average expression of genes in sample-specific clusters, and colors indicate the relative expression of genes. **E** Top 5 genes encoding surface molecules, ranked by log_2_-fold change, which are differentially expressed in each AML program (“Supplementary Methods” section). Bubble size = the proportion of cells expressing the gene; bubble color = log_2_-fold change. **F** Relative differentiation of single cells assigned to each AML program, across timepoints, using CytoTRACE. **G** Percentage of AML single cells assigned to each program at 0 and 24 h timepoints.

To identify CD4^IL10^ killing-sensitive and -resistant primary AML samples for scRNA-seq analysis, we performed killing assays (“Methods” section) and determined that AML samples PARCEV and PARCHW were sensitive (% Elimination Efficiency, EE, >50%), while AML samples PATISD and PAPZCL were resistant to CD4^IL10^ killing (% EE < 25%) (Figs. 1A and S1; Table S1) [15]. CD4^IL10^ cells expressed the expected phenotype [15, 16], including CD2, CD18, CD226, and other surface proteins (Fig. S2).

To understand how sensitive and resistant AML cells as well as CD4^IL10^ cells change after their interaction, we performed scRNA-seq before (0 h) and 24 h after (24 h) AML-CD4^IL10^ cell co-culture (Fig. S3A). AML and CD4^IL10^ cells were sorted for live cells immediately before single-cell capture to ensure high sample quality (“Methods” section). Unlike in the 3-day killing assay (Fig. S1), 24-h co-culture did not result in complete elimination of sensitive AML cells by CD4^IL10^ cells (Fig. S3B).

Following pre-processing, 72,095 single cells were considered for downstream analysis. Of those, 47,521 cells were AML and 24,574 were CD4^IL10^ cells (Supplementary Methods; Table S3). AML cells showed heterogeneity between patients, but not between replicates (Fig. 1B), showing that sample processing introduced minimal variability. CD4^IL10^ cells displayed less heterogeneity than AML (Fig. 1B). Transcriptome of both AML and CD4^IL10^ cells separated by timepoint, suggesting that in vitro incubation induced transcriptional changes (Fig. 1B).

### AML samples contain cells expressing four different transcriptional programs

To resolve the heterogeneity of AML samples while mitigating patient- and in vitro-induced differences, we employed a two-tiered approach. We first clustered AML single cells within each patient at each timepoint (Supplementary Methods; Fig. S4A), then calculated the average transcriptome per cluster. The averages were then clustered, revealing five AML cell transcriptional programs shared across all patients and timepoints (Fig. S4B, C). One of the programs was low quality (<200 overexpressed markers and >20% of mitochondrial reads; Fig. S4D) and was removed from the analysis.

The remaining four programs revealed AML transcriptional heterogeneity (Figs. 1C, D, and S4E; Table S3). Program P1 was enriched mostly in cells from killing-sensitive AML samples at 0 h. Programs P2 and P3 were enriched in all samples and conditions, while program P4 was found in cells mostly after 24 h of co-culture (Fig. 1D). Top 5 genes encoding surface proteins per program are depicted in Fig. 1E (complete list in Table S3). Cells enriched in program P1 overexpressed genes associated with mature monocytes or myeloid cells, such as *S100A8*, *S100A9, CD68*, *CD14*, integrin- and adhesion protein-encoding genes *ITGAX* and *ITGB2*, *ICAM1* (CD54), and cytokine or cytokine receptor-encoding genes such as *IL10RA*, *TNFRSF1B*, and *IFNGR1*. We did not find notable immune-related genes in program P2. Cells enriched in program P3 overexpressed *CD34*, which is found in hematopoietic stem cells (HSC) and leukemic stem cells (LSC) [25]. AML cells enriched in program P4 overexpressed genes encoding HLA class II and *CD200*, a glycoprotein found on AML and LSC [26] that mediates AML resistance to CD4^IL10^ killing [15] (Fig. 1E, Table S3).

Because AML programs contained genes associated with hematopoietic cell maturation, we assessed the relative differentiation stage of AML cells in programs P1-P4 using CytoTRACE [27]. AML cells in program P1 had the highest, whereas AML cells in programs P2-P4 had lower estimated differentiation (Fig. 1F). Cells that survived 24 h culture, irrespective of the program, displayed a less differentiated signature (*p* < 10^−16^, Wilcoxon test; Fig. 1F). For two samples with reported French-American-British (FAB) AML classification [28] (Table S1), which is based on morphology and differentiation stage, CytoTRACE differentiation staging was in accordance with the FAB; sensitive PARCEV sample was classified as myelomonocytic AML (FAB M4), while resistant PAPZCL was classified as undifferentiated AML (FAB M0).

Next, we examined the representation of AML programs across time. Program P1 was initially present in sensitive AML but disappeared after 24 h (Fig. 1G). Representation of AML cells expressing program P2 was low, and decreased in some, but not all samples after co-culture. The majority of AML cells expressed program P3, which was stable over time, while the representation of AML cells expressing program P4 increased after co-culture (Fig. 1G). Thus, program P1 is associated with sensitivity, while programs P3 and P4 are associated with resistance to CD4^IL10^ cell-mediated killing.

### AML transcriptional programs correlate with AML sensitivity and patient survival

**Fig. 2:**
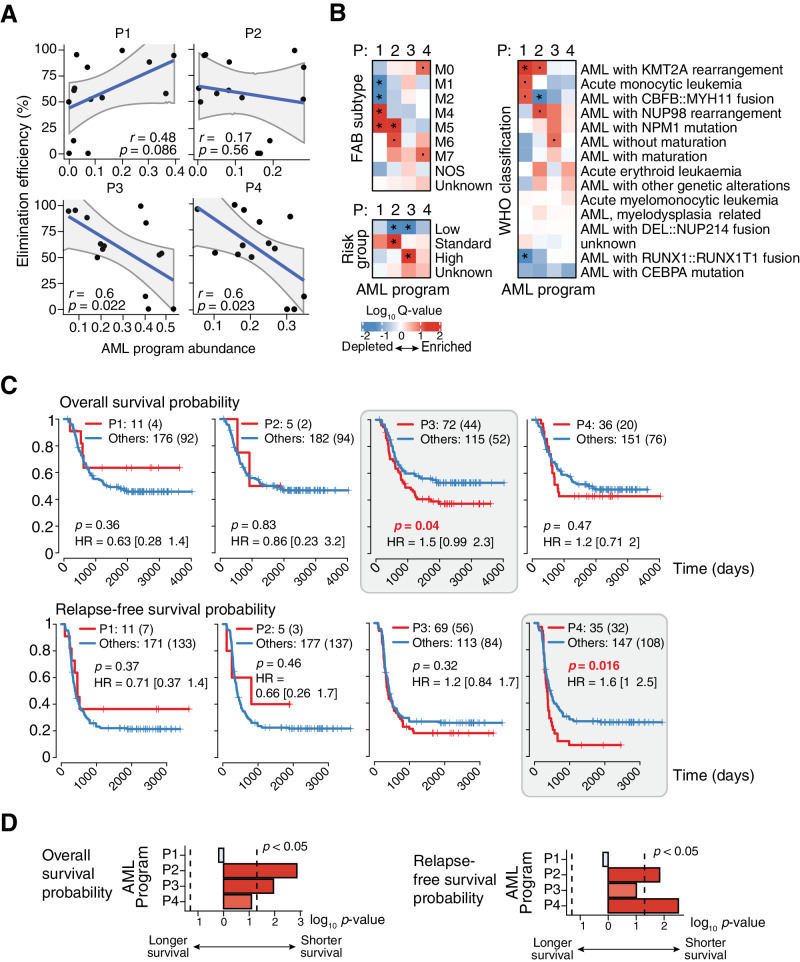
AML transcriptional programs that correlate with resistance to T cell killing also correlate with poor patient survival. **A** The association of AML program abundance with sensitivity to CD4^IL10^ killing across AML samples with known elimination efficiencies (*n* = 14) that were analyzed by bulk RNA-seq^7^ (Supplementary Methods). **B** Heatmap of the log_10_ *q*-values of the enrichment of AML programs in AML samples from the TARGET cohort (Supplementary Methods) with known French-American British (FAB) subtypes (top left), risk groups (bottom left), and WHO subtypes (right). Enrichment was calculated by performing Wilcoxon tests between the program abundance of samples from each subtype and the rest of the categories. * –*q* < 0.05, · –*q* < 0.1. **C** Kaplan–Meier plots of the association of samples from the TARGET cohort grouped by the dominant AML program with overall survival (top row) and relapse-free survival (bottom row). *p* – log-rank *p*-value, HR – hazard ratio. Numbers in square brackets indicate the 95% confidence interval of the hazard ratio, and the numbers in round brackets indicate the number of events. **D** The –log_10_ *p*-values of the association of AML program abundance with overall survival (left) and relapse-free survival (right), calculated using univariate Cox models.

Next, we correlated the abundance of each AML transcriptional program in bulk RNA-seq samples from our previously published AML cohort (*n* = 14) [15], which had known sensitivity to CD4^IL10^ killing. In line with scRNA-seq results, the abundance of program P1 positively correlated with killing, while the abundance of programs P3 and P4 showed a significant negative correlation with killing (Fig. 2A).

As tumor biology studies often utilize model cell lines, we analyzed the enrichment of AML programs P1-P4 in commercially available leukemia and lymphoma cell lines from the Cancer Cell Line Encyclopedia (CCLE) RNA-seq dataset [29] (Fig. S5A–C, Table S4). The cell lines enriched in program P1 included monocytic THP-1 and U937, which we use as killing-sensitive controls in CD4^IL10^ killing assays [13–15] (Fig. S5B, C). Other cell lines, such as the killing-resistant K562 with undifferentiated/erythroleukemic phenotype (negative control for CD4^IL10^ killing), exhibited low P1 but high P3 and/or P4 scores (Fig. S5B, C). Thus, the relative AML program abundance in RNA-seq data of tumor cell lines can indicate their sensitivity to CD4^IL10^ cell killing.

We also evaluated the association of AML transcriptional programs with the FAB classification [28], the WHO classification [30], and risk groups in a large cohort of pediatric AML samples from the National Cancer Institute (NCI) TARGET RNA-seq dataset (Figs. 2B and S5D**)**. Program P1, associated with sensitivity to killing, was significantly (*q* < 0.05) depleted from FAB subtypes M1 and M2 (acute myeloblastic leukemia with minimal maturation or with maturation, respectively), and significantly enriched in AML subtypes M4 (acute myelomonocytic leukemia) and M5 (acute monocytic leukemia) (Fig. 2B). P1 was also significantly enriched in WHO subtype AML with *KMT2A* rearrangement (*q* < 0.05), acute monocytic leukemia, and AML with *CBFB::MYH11* fusion (Fig. 2B). P1 program was not associated with risk groups, but it was significantly depleted from samples with *RUNX1::RUNX1T1* fusion (Fig. 2B), which is associated with worse prognosis in pediatric AML compared to patients without this mutation [31].

Program P2 (with undefined association to killing, Fig. 2A) was significantly enriched in FAB subtypes M5 (*q* < 0.05) and M6 (acute erythroid leukemia) and WHO subtypes AML with *KMT2A*, and *NUP98* rearrangement, which has a poor prognosis [32]. Program P2 was significantly depleted from AML with *CBFB::MYH11* fusion (*q* < 0.05), which is mainly associated with good survival [33]; significantly depleted from low-risk AML, and significantly enriched in AML with standard risk (Fig. 2B). Program P3, associated with resistance to killing, was enriched in AML without maturation, significantly (*q* < 0.05) enriched in high-risk AML, while significantly depleted from low-risk AML. Program P4, also associated with resistance to killing, was enriched in AML subtype M0 (undifferentiated acute myeloblastic leukemia) and M7 (acute megakaryoblastic leukemia; Fig. 2B). Altogether, AML sensitivity-associated program P1 was expressed in mature myeloid-like cells with mutations linked to better survival, while AML program P2 and resistance-associated programs P3 and P4 were expressed in less differentiated cells, and linked to higher risk AML.

Finally, we examined the association of AML programs with patient survival using the same TARGET RNA-seq dataset [15] (*n* = 187). Patients with enrichment of sensitivity-associated program P1 in their samples showed a non-significant trend towards better survival (Fig. 2C). In contrast, AML patients with enrichment of resistance-associated programs P3 and P4 had significantly reduced overall survival (OS) and relapse-free survival (RFS), respectively (Fig. 2C). These enrichments were also consistent when considering the abundance of programs as continuous variables in univariate Cox models, where program P2 was also found in patients with worse outcomes (Fig. 2D). After adjusting the analysis to account for the high-risk patients, we still observed the significant association of programs P2 and P4 with worse survival (Fig. S5E).

Altogether, AML programs identified by studying the AML/T cell interaction reinforce the importance of T cell response in AML elimination, and suggest that the enrichment of T cell killing-resistance programs in patient AML cells could indicate a risk of poor outcome.

### Only killing-sensitive AML cells activate CD4^IL10^ cells

**Fig. 3:**
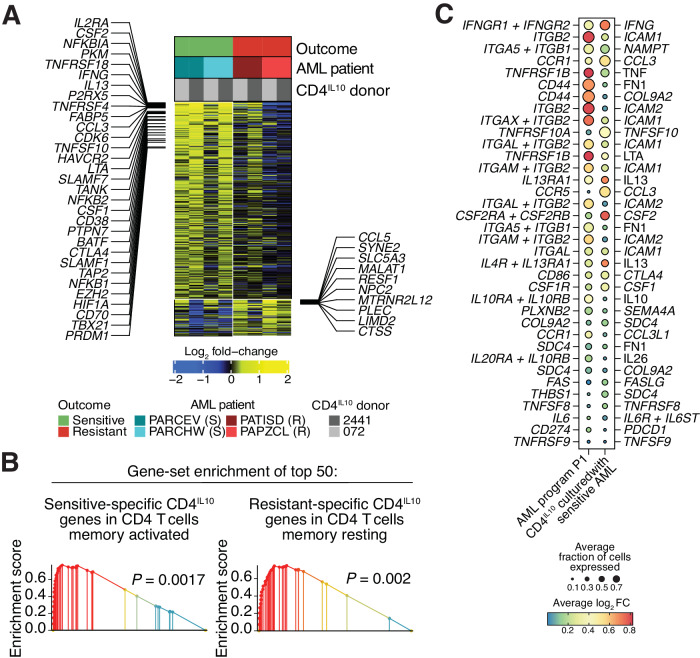
Killing-sensitive AML cells activate CD4^IL10^ cells. **A** Heatmap of the genes significantly changing in CD4^IL10^ cells after co-culture with sensitive or resistant AML cells (Supplementary Methods). Rows – genes, columns – CD4^IL10^ samples, colors – gene expression. **B** Gene-set enrichment of the top 50 genes uniquely overexpressed in CD4^IL10^ after co-culture with sensitive AML cells (left) and with resistant AML cells (right) (“Supplementary Methods”). **C**. Bubble plots representing the potential cell-cell interactions between AML cells expressing sensitivity-related program P1 and CD4^IL10^ cells co-cultured with sensitive AML (Supplementary Methods). Bubble sizes are proportional to the average fraction of single cells in each population expressing the genes encoding the first and second interaction partners. Colors indicate the average log_2_ fold change of genes encoding the interaction partners in the AML program P1 relative to other AML cells and in the CD4^IL10^ cells after culture with sensitive AML. The plus sign separates subunits of multi-subunit receptor complexes.

We next analyzed the transcriptional changes in CD4^IL10^ cells after interaction with sensitive or resistant AML cells (Fig. 3A). The transcriptome of CD4^IL10^ cells co-cultured with sensitive AML was markedly more perturbed than those with resistant AML (1,646 vs. 343 differentially expressed genes). This suggests that CD4^IL10^ cells make more (or higher affinity) interactions with sensitive AML compared to the resistant ones. CD4^IL10^ cells co-cultured with sensitive AML upregulated T cell activation-induced genes *IL2RA* (CD25) and *TNFRSF4* (OX40), inflammatory cytokines *CSF1* (M-CSF), *CSF2* (GM-CSF), *IFNG*, and *LTA* (lymphotoxin A), metabolism-related genes *FABP5* and *PKM*, and T cell activation-induced co-inhibitory molecules *HAVCR2* (TIM3) and *CTLA4*, among others (Fig. 3A; full list in Table S5). Gene-set enrichment analysis (GSEA) [34] revealed that CD4^IL10^ cells co-cultured with sensitive AML cells have a transcriptional signature of activated memory CD4^+^ T cells, while those co-cultured with resistant AML have a signature of resting memory CD4^+^ T cells (Figs. 3B and S6A). These data suggest that sensitive AML cells can engage and activate T cells, which facilitates their killing, whereas the resistant AML cells do not activate T cells and survive the interaction.

To identify potential receptor-ligand interactions between AML and CD4^IL10^ cells, we leveraged the CellChat database of receptor-ligand pairs [35] (Supplementary Methods), analyzing the genes upregulated in both AML and CD4^IL10^ cells after their interaction. For AML sensitivity-associated program P1, some of the top interacting pairs included AML genes *IFNGR1* and *TNFRSF1B*, which encode receptors for IFN-γ, TNF-α and LTA, and CD4^IL10^ cell cytokine genes *IFNG,* *TNF* and *LTA*. AML cells also upregulated integrins *ITGB2* (encoding CD18*), ITGAL* (CD11a), *ITGAM* (CD11b), and *ITGAX* (CD11c), which can bind to *ICAM1* (encoding ICAM1, also known as CD54) and *ICAM2* (CD102) upregulated on CD4^IL10^ cells (Fig. 3C).

AML cells enriched in resistance-associated programs P3 and P4 displayed a less abundant interaction profile with CD4^IL10^ cells. For AML program P3, the top interacting pair was between *LGALS9* (galectin-9) on AML cells and *CD44* on CD4^IL10^ cells (Fig. S6B**)**. For AML program P4, the top interacting pair was between *ICAM1*, here expressed on AML cells and *ITGAL* (CD11a) on CD4^IL10^ cells (Fig. S6C). Notably, while ICAM1 expression increased in P4-expressing AML cells after co-culture with CD4^IL10^ cells, the overall abundance of ICAM1 in resistant AML samples was very low compared to sensitive AML samples (Fig. S6D).

Altogether, sensitive AML successfully activates T cells, likely by making more cell-cell interactions, and are subsequently killed by them; whereas resistant AML fail to activate T cells and escape T cell killing.

### ICAM1/LFA-1 interaction is required for AML killing by CD4^IL10^ and CD8^+^ T cells

**Fig. 4:**
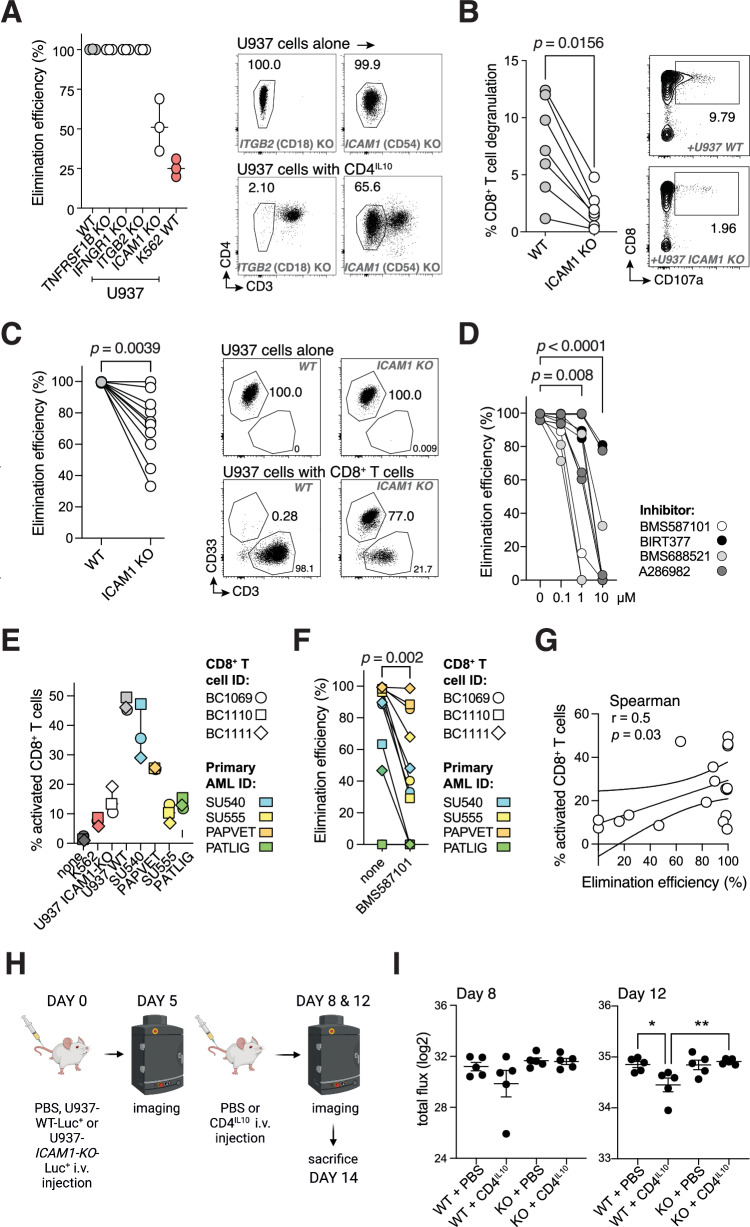
ICAM1/LFA-1 interaction is essential for AML killing by CD4^IL10^ and ex vivo-isolated CD8^+^ T cells. **A** Elimination efficiency of U937 cell line after CRISPR/Cas9-mediated knock-out (KO) of selected genes by CD4^IL10^ cells; each dot represents a CD4^IL10^ donor (*n* = 3). WT = wild type; U937 WT: positive control, K562 WT: negative control. Representative FACS plots from one donor against U937-*ITGB2*-KO and U937-*ICAM1*-KO are shown on the right; numbers indicate the percentage in the AML gate. **B** Degranulation of CD8^+^ T cells, expressed as % CD8^+^CD107a^+^ cells within live singlet CD3^+^ T cells, against U937 WT and U937-*ICAM1*-KO cells; each circle represents a CD8^+^ T cell donor (*n* = 7). Representative FACS plots from one donor are shown on the right; numbers indicate percent degranulating CD8^+^ T cells. **C** Elimination efficiency of U937 WT and U937-*ICAM1*-KO cells by CD8^+^ T cells (*n* = 10). Wilcoxon test, *p* < 0.05. Representative FACS plots from one donor are shown on the right; numbers indicate the percentage in the AML gate. **D** Elimination efficiency of U937 cell line 24 h after treatment with LFA-1 inhibitors at indicated concentrations; each line represents a CD4^IL10^ donor (*n* = 3); circles are color-coded by the inhibitor as indicated. Dunn’s post hoc test results are shown, following Friedman ANOVA (*p* < 0.0001). **E** CD8^+^ T cells were co-cultured for 24 h with indicated target cells; activation was measured as a percent of CD69^+^CD25^+/^^−^ cells within the live CD8^+^ T cell gate. **F** Elimination efficiency of indicated target cell lines and primary AML cells by CD8^+^ T cells after 3 days of co-culture in the absence or presence of LFA-1 inhibitor BMS587101 (10 μM). **G** Spearman correlation of percent activation of CD8^+^ T cells and the elimination efficiency of target cells. **H** Timeline of mice injections; *n* = 10 mice per group. **I** Graphs showing total flux (log normalized) obtained from imaging data on days 8 and 12. 1-way ANOVA with Bonferroni multiple comparison test; *p* = n.s. at day 8, *p* = 0.0075 at day 12.

Next, we knocked-out several highly expressed P1 genes identified by the interaction analysis from the killing-sensitive AML cell line U937 and quantified the knock-out effect on U937 sensitivity to CD4^IL10^ cell killing (“Methods” section). Knockout of TNF-α receptor (encoded by *TNFRSF1B*), IFN-γ receptor (*IFNGR1),* or CD18 integrin (*ITGB2*) in U937 cells did not alter their sensitivity to killing (Figs. 4A and S7A).

CD18 and CD11a are subunits of LFA-1 (lymphocyte function-associated antigen 1). LFA-1 on T cells binds to ICAM1 on target cells, enabling immune synapse formation and T cell-mediated killing [36]. CD4^IL10^ cells constitutively express high levels of CD18 [14] (*ITGB2*; Fig. S2) and CD11a [16] (*ITGAL*, Fig. S6E, F), which were not further upregulated in CD4^IL10^ cells interacting with killing-sensitive AML cells, and thus not identified by interaction analysis (Fig. 3C). However, *ICAM1* is one of the top overexpressed genes in sensitivity-associated program P1 (Table S3). Therefore, we also knocked out *ICAM1* on U937 cells (U937-*ICAM1*-KO). ICAM1 knock-out significantly decreased U937 sensitivity to CD4^IL10^ killing (Figs. 4A and S7A). Thus, ICAM1 expression on AML is necessary for AML killing by CD4^IL10^ cells.

To test whether ICAM1/LFA-1 interaction governs AML killing by primary CD8^+^ T cells, which mediate anti-AML immunity in vivo [36, 37], we co-cultured human CD8^+^ T cells with K562 cells, wild-type U937 cells, or U937-*ICAM1*-KO cells. Like CD4^IL10^ cells, CD8^+^ T cells did not degranulate and kill resistant K562 cells (“Supplementary Methods” section, Fig. S7C). To bypass the endogenous TCRs on primary human CD8^+^ T cells, we treated U937 cells, which express Fc receptors, with soluble anti-CD3 antibody. These treated U937 cells provide a non-specific TCR stimulus together with their own co-stimulatory signals to primary CD8^+^ T cells. CD8^+^ T cells degranulated and killed almost all wild-type U937 cells, whereas degranulation and killing of U937-*ICAM1*-KO cells by primary CD8^+^ T cells was significantly impaired (Fig. 4B, C). Thus, ICAM1 expression on AML is necessary for AML killing by primary CD8^+^ T cells.

To understand whether the ICAM1 receptor on T cells, LFA-1, is equally important as ICAM1 for AML sensitivity to killing, we added small molecule inhibitors of LFA-1 or ICAM1/LFA-1 interaction to the AML/CD4^IL10^ cell co-culture. All four inhibitors significantly increased the resistance of wild-type U937 cells to CD4^IL10^ killing in a dose-dependent manner (Fig. 4D), further confirming the role of ICAM1/LFA-1 interaction for effective AML killing by T cells.

Furthermore, we showed that only the killing-sensitive, wild-type U937, but not resistant U937-*ICAM1*-KO cells, potently induce primary CD8^+^ T cell activation, measured by frequency of CD69^+^CD25^+/^^−^ CD8^+^ T cells after 24 h co-culture (Figs. 4E and S7D). We also co-cultured CD8^+^ T cells with primary adult (SU540, SU555) and pediatric (PAPVET, PATLIG) AML samples, which we previously categorized as sensitive to CD4^IL10^ killing. Out of these AML samples, two induced strong CD8^+^ T cell activation (Figs. 4E and S7D), and three were killed by CD8^+^ T cells (Fig. 4F). Sample PATLIG expressed low CD64 and ICAM1, either of which could explain its resistance to killing, as low CD64 expression is likely insufficient to cross-link the anti-CD3 antibody (Fig. S8A, B). When ICAM1 was expressed on AML, LFA-1 inhibitor significantly impaired primary CD8^+^ T cell killing of primary AML (Figs. 4F, S7D, and S8A, B). Accordingly, we also observed a positive correlation between the degree of CD8^+^ T cell activation at 24 h and AML killing (Fig. 4G).

Finally, we injected U937-WT-Luciferase (Luc)^+^ or U937-*ICAM1-KO*-Luc^+^ cells into NSG mice. Five days later, we administered CD4^IL10^ cells into half of the U937-treated mice, and imaged them to follow tumor progression (Figs. 4H, I and S9). On days 8 and 12, we observed a reduction in tumor burden in CD4^IL10^-treated U937-WT, but not in the *ICAM1-KO* group; the reduction on day 12 was statistically significant (Fig. 4I). Thus, ICAM1 expression on killing-sensitive AML cells contributes to AML clearance by CD4^IL10^ cells in vivo. Altogether, these data suggest that ICAM1/LFA-1 interaction is important in AML elimination by cytotoxic CD4^+^ and CD8^+^ T cells, which could influence AML control in vivo.

## Discussion

In this study, we leveraged longitudinal single-cell transcriptomics to understand the determinants of AML sensitivity or resistance to T cell killing. We found that transcriptional programs P3 and P4, enriched in AML cells resistant to CD4^IL10^ cell killing, were significantly associated with poor patient survival. Providing this association is validated in independent cohorts, genes in programs P3 and P4 could be derived into useful biomarkers to identify high-risk patients suitable for a more aggressive therapeutic approach. Genetic aberrations with well-defined risks are not present in all patients [38], leaving many AML patients without prognostic biomarkers [39, 40].

CD4^IL10^ cells recognize and kill most primary AML cells in vitro independent of antigen recognition via TCR [13–15], and inhibit leukemia in vivo [14]. These properties make them useful to study T cell-AML interaction, bypassing the requirement to isolate rare AML antigen-specific T cells from AML patients [41]. CD200-mediated AML resistance to T cell killing was observed both on CD4^IL10^ cells and primary human CD8^+^ T cells, and we show herein that *ICAM1* expression on AML has a comparable effect on CD4^IL10^ cell and primary, ex vivo-isolated human CD8^+^ T cell function. Thus, CD4^IL10^ cells are a relevant model to study AML-T cell interaction.

Moreover, CD4^IL10^ cells can be developed as an immunotherapy for patients with killing-sensitive AML. Post allo-HSCT (allogeneic hematopoietic stem cell transplantation), CD4^IL10^ cells would inhibit graft vs host disease (GvHD) via secretion of IL-10, while boosting graft vs leukemia (GvL). Also, CD4^IL10^ cells could be a less toxic alternative to 2nd round of consolidation chemotherapy in high-risk patients pre-HSCT. Currently, there is an effort to develop therapeutic strategies for leukemias that increase overall T cell function but do not solely depend on a single antigen recognition [42]. CD4^IL10^ cells meet these requirements thanks to their antigen-independent killing. Importantly, top-upregulated genes in CD4^IL10^ cells co-cultured with sensitive AML included IFN-γ, which counteracts the loss of HLA class II expression observed in AML relapse [10, 11]. Besides IFN-γ, CD4^IL10^ cells co-cultured with sensitive AML produced pro-inflammatory TNF-α and LTA. These data illustrate that, despite constitutive expression of *IL10*, CD4^IL10^ cells predominantly activate their effector rather than immune regulatory functions when interacting with AML.

We also show herein that a key interaction that defined AML sensitivity to killing by CD4^IL10^ cells and primary human CD8^+^ T cells is mediated by ICAM1, which facilitates immune synapse formation with T cells. These data add a mechanistic basis to existing studies showing that functional T cell responses are important for AML patient survival [4, 5]. AML cells in patients refractory to HSCT downregulate the HLA II genes [10, 11], and deregulate co-stimulatory molecules [11], both of which are critical to activate CD4^+^ T cells that help CD8^+^ T cells. In AML patients that relapse after HSCT, memory T cells appear exhausted [43]. AML patients that do not respond to chemotherapy have senescent T cells [7], which diminishes their capacity to kill autologous AML cells [8]. CD8^+^ T cells from AML patients did not make functional immune synapses, thus failing to kill AML [5].

Our study also has some limitations. The size of our longitudinal scRNA-seq sample set is limited and analysis of additional AML samples may reveal other mechanisms of AML sensitivity. Nevertheless, our scRNA-seq includes over 40,000 single AML cells and longitudinal analysis that led to novel results, increasing our understanding of AML/T cell interactions and identifying potential therapeutic approaches. Another limitation is the sample size of the public dataset used in the outcome analysis, which did not permit detailed patient stratification according to treatment regimens and other clinical data.

In summary, we reveal that AML cells express transcriptional programs associated with sensitivity or resistance to T cell killing. Sensitivity-associated programs are found in mature myeloid-like AML, while resistance-associated programs are found in stem cell-like AML and linked to poor survival. Moreover, we show that killing-sensitive, but not resistant AML cells, activate CD4^IL10^ cells and primary CD8^+^ T cells. Disruption of only one interaction, between ICAM1 on sensitive AML cells and LFA-1 on T cells, is sufficient to increase AML resistance to killing both in vitro and in vivo by CD4^IL10^ cells, and in vitro by primary CD8^+^ T cells. ICAM1 thus acts as an immune trigger, with an opposing function to immune checkpoint proteins, and it is possible that a therapeutic enhancement of ICAM1/LFA-1 interaction between AML and T cells could enable T cell activation and tumor control. Overall, this study highlights the importance of AML-T cell interactions in AML immune escape, suggesting that the ability of AML tumor cells to productively engage T cells is important for AML patient survival.

### Supplementary information

Supplementary Methods

Supplementary figure legends

Supplemental Material

## Data Availability

scRNA-seq data have been deposited in the Genome Expression Omnibus, accession number GSE254282.

## References

[1] Jacobsohn DA, Loken MR, Fei M, Adams A, Brodersen LE, Logan BR (2018). Outcomes of measurable residual disease in pediatric acute myeloid leukemia before and after hematopoietic stem cell transplant: validation of difference from normal flow cytometry with chimerism studies and Wilms tumor 1 gene expression. Biol Blood Marrow Transplant.

[2] Klein K, de Haas V, Kaspers GJL (2018). Clinical challenges in de novo pediatric acute myeloid leukemia. Expert Rev Anticancer Ther.

[3] De Kouchkovsky I, Abdul-Hay M (2016). Acute myeloid leukemia: a comprehensive review and 2016 update. Blood Cancer J.

[4] Ismail MM, Abdulateef NAB (2017). Bone marrow T-cell percentage: a novel prognostic indicator in acute myeloid leukemia. Int J Hematol.

[5] Le Dieu R, Taussig DC, Ramsay AG, Mitter R, Miraki-Moud F, Fatah R (2009). Peripheral blood T cells in acute myeloid leukemia (AML) patients at diagnosis have abnormal phenotype and genotype and form defective immune synapses with AML blasts. Blood.

[6] Durgeau A, Virk Y, Corgnac S, Mami-Chouaib F (2018). Recent advances in targeting CD8 T-cell immunity for more effective cancer immunotherapy. Front Immunol.

[7] Knaus HA, Berglund S, Hackl H, Blackford AL, Zeidner JF, Montiel-Esparza R (2018). Signatures of CD8+ T cell dysfunction in AML patients and their reversibility with response to chemotherapy. JCI Insight.

[8] Rutella S, Vadakekolathu J, Mazziotta F, Reeder S, Yau TO, Mukhopadhyay R (2022). Immune dysfunction signatures predict outcomes and define checkpoint blockade-unresponsive microenvironments in acute myeloid leukemia. J Clin Invest.

[9] Li Z, Philip M, Ferrell PB (2020). Alterations of T-cell-mediated immunity in acute myeloid leukemia. Oncogene.

[10] Christopher MJ, Petti AA, Rettig MP, Miller CA, Chendamarai E, Duncavage EJ (2018). Immune escape of relapsed AML cells after allogeneic transplantation. N Engl J Med.

[11] Toffalori C, Zito L, Gambacorta V, Riba M, Oliveira G, Bucci G (2019). Immune signature drives leukemia escape and relapse after hematopoietic cell transplantation. Nat Med.

[12] Tay RE, Richardson EK, Toh HC (2021). Revisiting the role of CD4(+) T cells in cancer immunotherapy-new insights into old paradigms. Cancer Gene Ther.

[13] Andolfi G, Fousteri G, Rossetti M, Magnani CF, Jofra T, Locafaro G (2012). Enforced IL-10 expression confers type 1 regulatory T cell (Tr1) phenotype and function to human CD4(+) T cells. Mol Ther.

[14] Locafaro G, Andolfi G, Russo F, Cesana L, Spinelli A, Camisa B (2017). IL-10-engineered human CD4(+) Tr1 cells eliminate myeloid leukemia in an HLA class I-dependent mechanism. Mol Ther.

[15] Cieniewicz B, Uyeda MJ, Chen PP, Sayitoglu EC, Liu JM, Andolfi G (2021). Engineered type 1 regulatory T cells designed for clinical use kill primary pediatric acute myeloid leukemia cells. Haematologica.

[16] Liu JM, Chen P, Uyeda MJ, Cieniewicz B, Sayitoglu EC, Thomas BC (2021). Pre-clinical development and molecular characterization of an engineered type 1 regulatory T-cell product suitable for immunotherapy. Cytotherapy.

[17] Guo Y, Xie YQ, Gao M, Zhao Y, Franco F, Wenes M (2021). Metabolic reprogramming of terminally exhausted CD8(+) T cells by IL-10 enhances anti-tumor immunity. Nat Immunol.

[18] Chan IH, Wu V, Bilardello M, Mar E, Oft M, Van Vlasselaer P (2015). The potentiation of IFN-gamma and induction of cytotoxic proteins by pegylated IL-10 in human CD8 T cells. J Interferon Cytokine Res.

[19] Magnani CF, Alberigo G, Bacchetta R, Serafini G, Andreani M, Roncarolo MG (2011). Killing of myeloid APCs via HLA class I, CD2 and CD226 defines a novel mechanism of suppression by human Tr1 cells. Eur J Immunol.

[20] Coles SJ, Hills RK, Wang EC, Burnett AK, Man S, Darley RL (2012). Expression of CD200 on AML blasts directly suppresses memory T-cell function. Leukemia.

[21] Coles SJ, Wang EC, Man S, Hills RK, Burnett AK, Tonks A (2011). CD200 expression suppresses natural killer cell function and directly inhibits patient anti-tumor response in acute myeloid leukemia. Leukemia.

[22] Uyeda MJ, Freeborn RA, Cieniewicz B, Romano R, Chen PP, Liu JM (2021). BHLHE40 regulates IL-10 and IFN-gamma production in T Cells but does not interfere with human type 1 regulatory T cell differentiation. Front Immunol.

[23] Zheng GX, Terry JM, Belgrader P, Ryvkin P, Bent ZW, Wilson R (2017). Massively parallel digital transcriptional profiling of single cells. Nat Commun.

[24] Hao Y, Hao S, Andersen-Nissen E, Mauck WM, Zheng S, Butler A (2021). Integrated analysis of multimodal single-cell data. Cell..

[25] O’Reilly E, Zeinabad HA, Szegezdi E (2021). Hematopoietic versus leukemic stem cell quiescence: challenges and therapeutic opportunities. Blood Rev.

[26] Ho JM, Dobson SM, Voisin V, McLeod J, Kennedy JA, Mitchell A (2020). CD200 expression marks leukemia stem cells in human AML. Blood Adv.

[27] Gulati GS, Sikandar SS, Wesche DJ, Manjunath A, Bharadwaj A, Berger MJ (2020). Single-cell transcriptional diversity is a hallmark of developmental potential. Science.

[28] Bennett JM, Catovsky D, Daniel MT, Flandrin G, Galton DA, Gralnick HR (1976). Proposals for the classification of the acute leukaemias. French-American-British (FAB) co-operative group. Br J Haematol.

[29] Ghandi M, Huang FW, Jane-Valbuena J, Kryukov GV, Lo CC, McDonald ER (2019). Next-generation characterization of the Cancer Cell Line Encyclopedia. Nature..

[30] Khoury JD, Solary E, Abla O, Akkari Y, Alaggio R, Apperley JF (2022). The 5th edition of the World Health Organization Classification of Haematolymphoid Tumours: myeloid and histiocytic/dendritic neoplasms. Leukemia.

[31] Yamato G, Shiba N, Yoshida K, Hara Y, Shiraishi Y, Ohki K (2018). RUNX1 mutations in pediatric acute myeloid leukemia are associated with distinct genetic features and an inferior prognosis. Blood.

[32] Struski S, Lagarde S, Bories P, Puiseux C, Prade N, Cuccuini W (2017). NUP98 is rearranged in 3.8% of pediatric AML forming a clinical and molecular homogenous group with a poor prognosis. Leukemia.

[33] Huang BJ, Smith JL, Wang YC, Taghizadeh K, Leonti AR, Ries RE (2021). CBFB-MYH11 fusion transcripts distinguish acute myeloid leukemias with distinct molecular landscapes and outcomes. Blood Adv.

[34] Newman AM, Liu CL, Green MR, Gentles AJ, Feng W, Xu Y (2015). Robust enumeration of cell subsets from tissue expression profiles. Nat Methods.

[35] Jin S, Guerrero-Juarez CF, Zhang L, Chang I, Ramos R, Kuan CH (2021). Inference and analysis of cell-cell communication using CellChat. Nat Commun.

[36] Reina M, Espel E. Role of LFA-1 and ICAM-1 in cancer. Cancers. 2017;9. 10.3390/cancers9110153 PMC5704171 29099772

[37] Schmits R, Kundig TM, Baker DM, Shumaker G, Simard JJ, Duncan G (1996). LFA-1-deficient mice show normal CTL responses to virus but fail to reject immunogenic tumor. J Exp Med.

[38] Padmakumar D, Chandraprabha VR, Gopinath P, Vimala Devi ART, Anitha GRJ, Sreelatha MM (2021). A concise review on the molecular genetics of acute myeloid leukemia. Leuk Res.

[39] Lonetti A, Pession A, Masetti R (2019). Targeted therapies for pediatric AML: gaps and perspective. Front Pediatr.

[40] Jones LM, Tarlock K, Cooper T (2021). Targeted therapy in pediatric AML: an evolving landscape. Paediatr Drugs.

[41] Scheibenbogen C, Letsch A, Thiel E, Schmittel A, Mailaender V, Baerwolf S (2002). CD8 T-cell responses to Wilms tumor gene product WT1 and proteinase 3 in patients with acute myeloid leukemia. Blood.

[42] Daver N, Alotaibi AS, Bucklein V, Subklewe M (2021). T-cell-based immunotherapy of acute myeloid leukemia: current concepts and future developments. Leukemia.

[43] Noviello M, Manfredi F, Ruggiero E, Perini T, Oliveira G, Cortesi F (2019). Bone marrow central memory and memory stem T-cell exhaustion in AML patients relapsing after HSCT. Nat Commun.

