# Supplementary material for: AML/T cell interactomics uncover correlates of patient outcomes and the key role of ICAM1 in T cell killing of AML
Source: Leukemia. 2024 May 9;38(6):1246–55. doi: 10.1038/s41375-024-02255-1 (PMC11147760; doi:10.1038/s41375-024-02255-1)
Supplement: Supplementary file 1 — Supplementary Methods [file 41375_2024_2255_MOESM1_ESM.docx]

**Supplementary Methods**

*Killing assay and degranulation assay details.* In CD8^+^ T cell killing assays, sensitive U937 and resistant K562 cells were pre-treated with 200 ng/ml soluble anti-CD3 monoclonal antibody (mAb; OKT3, Miltenyi Biotec) for 15 min at 37^o^C, allowing the anti-CD3 mAb bind to Fc-receptors on U937 cells (control K652 cells do not express Fc receptors). Next, freshly isolated human CD8^+^ T cells were added to pre-treated target cells at a 1:1 E:T ratio, and co-cultured for 3 days with 50 IU/ml IL-2 and a final concentration of 50 ng/ml anti-CD3 mAb. Remaining cells were then quantified via flow cytometry and counting beads (C36950, Thermo Fisher Scientific, MA, US) (1). For CD8^+^ T cell degranulation assay, target cells were pre-treated with anti-CD3 mAb as above, and co-cultured with human CD8^+^ T cells at 1:1 E:T ratio with anti-CD107a-APC mAb (BD Biosciences, CA, USA). After 1 h, brefeldin A and monensin (BioLegend, CA, USA; used as following manufacturer’s instructions) were added to each well, cells were cultured for 4 more hours, stained with a viability dye and anti-CD3 and anti-CD8 mAb (**Table S2**).

*Single cell RNA data preprocessing.* After loading the 10X Genomics CellRanger output into Seurat 4.0.5, genes expressed in less than 3 cells and ribosomal genes were filtered out, and 28,570 genes remained. Cells expressing less than 500 reads and/or 500 features, and more than 50% mitochondrial reads were also filtered out. The mitochondrial cut-off was higher than suggested in Seurat (2) vignette describing *ex-vivo* isolated peripheral blood mononuclear cell (PBMC) analysis, because cultured AML cells and CD4^IL10^ cells are expected to be more metabolically active than resting PBMC. Notably, both AML and CD4^IL10^ cells at each time point were FACS-sorted for live cells, and 10X capture and processing for scRNA-seq started within 1h post-sort.

*Bulk RNA data preprocessing.* NCI TARGET bulk- RNA-seq dataset and the dataset used in **Figure 3A** were processed using STAR (3), as described previously (1). For each dataset, the transcript-per-million (TPM) values were used. TPM profiles of cell-lines from Cancer Cell Line Encyclopedia (CCLE) were downloaded from the Broad Institute’s website (https://data.broadinstitute.org/ccle/CCLE_RNAseq_rsem_genes_tpm_20180929.txt). Gene IDs were mapped to gene symbols using EnsDb.Hsapiens.v86 (4) package. The expression matrix was filtered for protein-coding genes and hematopoietic cell-lines. The resulting expression values were re-converted to TPM.

*Cell type annotation.* Cells were assigned to cell types using marker genes *ANPEP* (CD13), *CD33,* *CD3D* and *CD3E*. The Wilcoxon test was used to assess differential gene expression between clusters, and *p*-values were adjusted for multiple hypothesis testing using the Benjamini-Hochberg method. Clusters with a *q*-value < 0.05 for *ANPEP* or *CD33* were considered AML, and those with *q*-value < 0.05 for *CD3D* or *CD3E* were considered CD4^IL10^ cells. Clusters over-expressing both AML and CD4^IL10^ markers, or none of the two, were filtered out.

*Identification of AML transcriptional programs.* We first clustered AML cells from each patient/timepoint pair, then averaged the expression of each gene across cells from each patient/timepoint pair within each cluster and merged the resulting average transcriptomes into a single matrix, $T^{*}$. We filtered out clusters (columns) with < 25 cells and genes (rows) with no expression, resulting in matrix $T$. We identified AML cell programs by applying non-negative matrix factorization (NMF) (5) on matrix $T$ using 25 NMF restarts as published (6).

*Selection of AML transcriptional programs.* We selected the number of programs at which the Cophenetic coefficient fell below 0.99 and ignored programs with less than 200 marker genes in the downstream analysis. To assign single cells and external expression data to the programs, the recovery procedure described in Luca et al (6) was used. The single cells and bulk samples for which the abundance of the filtered programs was highest were considered unassigned. We used a recovery procedure (6) to assign external expression data to programs, considering the single cells and bulk samples with the highest abundance of the filtered program as unassigned.

*AML-induced CD4^IL10^ cell transcriptional changes.* DGE analysis was performed between CD4^IL10^ cells before and after co-culture with each AML sample. The Wilcoxon test z-scores of CD4^IL10^ cells co-cultured with sensitive and resistant AML cells were separately combined into meta-z scores (7), with the natural logarithm of the number of cells as weights and converted to two-sided *p*-values. Genes with a *q*-value < 0.05 (Benjamini-Hochberg procedure) in only one of the two conditions were analyzed further.

*Ligand-receptor analysis*. Potential ligand-receptor interactions between AML and CD4^IL10^ were identified using the CellChat database (8), focusing on overexpressed genes in AML programs and in CD4^IL10^ cells after co-culture with sensitive or resistant AML, and interactions for which all ligand genes were overexpressed in an AML program and all receptor genes in the CD4^IL10^ cells, or vice versa.

*Seurat analysis.* Raw counts were scaled to 10,000 reads per cell, and log-normalized. The top 2000 genes were selected using the *vst* method implemented in function *FindVariableFeatures*, run with the default parameters. Clusters of cells were identified by *FindNeighbors*, using the top 30 PCA components and otherwise default parameters, and *FindClusters,* with the resolution parameter set to 0.8 and otherwise default parameters. UMAP plots were generated using the top 30 PCA components with the *RunUMAP* function.

*Differential gene expression analysis.* Unless otherwise specified, DGE analysis was performed using the function *FindMarkers,* run with parameters: mean.fxn = rowMeans, min.cells.group = 0, min.cells.feature = 0, min.pct = 0, logfc.threshold = 0, only.pos = FALSE, max.cells.per.ident = 500, return.thresh = 1.

*Gene-set enrichment analysis.* We leveraged full transcriptomes of T cells: CD4 naïve, T cells: CD4 memory resting, and T cells: CD4 memory activated from Newman et al (9). For each cell population, we ordered each gene in the transcriptome by calculating the average log_2_ fold change of each population relative to the others. From the gene lists identified as described in section *AML-induced CD4^IL10^ cell transcriptional changes*, top 50 with the highest average log_2_ fold change across CD4^IL10^ cells cultured with sensitive and resistant samples were evaluated in mean log2 fold change-ordered transcriptomes using pre-ranked Gene Set Enrichment Analysis (GSEA) (*fgsea* R package)(10), with 1,000 permutations.

*Optimization of published CRISPR/Cas9 knock-out strategy (11)*. As previously described (11), we used the kits (Synthego, CA, USA) that contained 3 individual single-guide (12) RNAs per target to knock out individual *IFNGR1, TNFRSF1B,* and *ITGB2*. Ribonucleoprotein (RNP) complexes were generated by mixing sgRNAs (30 pmol each) with 62 pmol of HiFi Cas9 (Integrated DNA Technologies, IA, USA). We optimized the published protocol by adding of 4 μM electroporation enhancer (Integrated DNA Technologies, IA, USA) and using the SF Cell Line nucleofection solution (Lonza, Switzerland) for the sensitive AML cell line U937. *ICAM1* knockout was made by first cloning an *ICAM1*-targeting sgRNA into a modified lentiCRISPRv2 plasmid with an RFP reporter instead of puromycin (RFP subclone kindly provided by Prof. Ravindra Majeti; original clone was a gift from Feng Zhang, Addgene plasmid #52961). Third generation lentivirus was produced as described (1). RFP^+^ICAM1^-^ cells were FACS-sorted one week post transduction. Knockout was confirmed by flow cytometry or Sanger sequencing ≥ 5 days after editing.

*U937 in vivo experiments*. U937 WT or *ICAM1-KO* cells were transduced with the lentiviral vector expressing GFP-Luciferase (plasmid was a kind gift from Dr. Le Cong) and sorted via FACS based on GFP and ICAM-1 expression. All NSG mice (all male because U937 cell line originated from a male patient) were purchased from Jackson Labs (Bar Harbor, ME) at 5 - 6 weeks of age. PBS, 1 - 1.4 million U937-WT-Luc^+^ or U937-*ICAM1-KO*-Luc^+^ cells were injected to mice (n = 10 mice per cohort, randomly grouped and no blinding was done) at 8 weeks of age intravenously. Imaging was performed using IVIS imaging system (PerkinElmer, Shelton, CT) after intraperitoneal luciferin (Promega, Madison, WI) injections according to the manufacturer’s protocol. Five days post U937 or PBS injection, half of the mice in each cohort (n = 5 per group) were injected with 2 million CD4^IL10^ cells intravenously. Imaging was done on days 8 and 12 post U937 injection, and mice were sacrificed on day 14. The number of mice per group were selected based on a previously published study using U937-Luc cells in NSG mice (13). All *in vivo* studies were approved by the Administrative Panel on Laboratory Animal Care (APLAC) at Stanford University and were in compliance with the ethical guidelines for animal welfare.

**REFERENCES:**

1. Cieniewicz B, Uyeda MJ, Chen PP, Sayitoglu EC, Liu JM, Andolfi G, et al. Engineered type 1 regulatory T cells designed for clinical use kill primary pediatric acute myeloid leukemia cells. Haematologica. 2020;Online ahead of print.

2. Hao Y, Hao S, Andersen-Nissen E, Mauck WM, 3rd, Zheng S, Butler A, et al. Integrated analysis of multimodal single-cell data. Cell. 2021;184(13):3573-87 e29.

3. Dobin A, Davis CA, Schlesinger F, Drenkow J, Zaleski C, Jha S, et al. STAR: ultrafast universal RNA-seq aligner. Bioinformatics. 2013;29(1):15-21.

4. Rainer J. R package version 2.99. 0. EnsDb Hsapiens v86: Ensembl based annotation package. 2017.

5. Brunet JP, Tamayo P, Golub TR, Mesirov JP. Metagenes and molecular pattern discovery using matrix factorization. Proc Natl Acad Sci U S A. 2004;101(12):4164-9.

6. Luca BA, Steen CB, Matusiak M, Azizi A, Varma S, Zhu C, et al. Atlas of clinically distinct cell states and ecosystems across human solid tumors. Cell. 2021;184(21):5482-96 e28.

7. Lipták T. On the combination of independent tests. Magyar Tud Akad Mat Kutato Int Közl. 1958;3:171-96.

8. Jin S, Guerrero-Juarez CF, Zhang L, Chang I, Ramos R, Kuan CH, et al. Inference and analysis of cell-cell communication using CellChat. Nat Commun. 2021;12(1):1088.

9. Newman AM, Liu CL, Green MR, Gentles AJ, Feng W, Xu Y, et al. Robust enumeration of cell subsets from tissue expression profiles. Nat Methods. 2015;12(5):453-7.

10. Korotkevich G, Sukhov V, Budin N, Shpak B, Artyomov MN, Sergushichev A. Fast gene set enrichment analysis. bioRxiv. 2021:060012.

11. Uyeda MJ, Freeborn RA, Cieniewicz B, Romano R, Chen PP, Liu JM, et al. BHLHE40 Regulates IL-10 and IFN-gamma Production in T Cells but Does Not Interfere With Human Type 1 Regulatory T Cell Differentiation. Front Immunol. 2021;12:683680.

12. Taghiloo S, Asgarian-Omran H. Immune evasion mechanisms in acute myeloid leukemia: A focus on immune checkpoint pathways. Crit Rev Oncol Hematol. 2021;157:103164.

13. Wang J, Chen S, Xiao W, Li W, Wang L, Yang S, et al. CAR-T cells targeting CLL-1 as an approach to treat acute myeloid leukemia. J Hematol Oncol. 2018;11(1):7.
