# Supplementary material for: AML/T cell interactomics uncover correlates of patient outcomes and the key role of ICAM1 in T cell killing of AML
Source: Leukemia. 2024 May 9;38(6):1246–55. doi: 10.1038/s41375-024-02255-1 (PMC11147760; doi:10.1038/s41375-024-02255-1)
Supplement: Supplementary file 2 — Supplementary figure legends [file 41375_2024_2255_MOESM2_ESM.docx]

**Supplementary figure legends:**

**Figure S1. Flow cytometry-based killing assay. A.** Killing assay gating strategy, depicting representative dot plots of effector (CD4^IL10^ cells) and target cells (AML cells) after 3-day co-culture at 1:1 ratio. CountBright beads (Thermo Fisher) are added to each sample, and cells quantified according to manufacturer’s instructions. In the top-left dot plot, beads can be seen on the upper left corner (9.56%). After gating for single and live cells, CD45^+^CD33^+^ cells are considered to be AML cells, and NGFR^+^ cells T cells (CD4^IL10^ cells are engineered to express truncated NGFR marker gene). **B.** Killing assay results, representative flow cytometry plots. Raw cell counts recorded in AML gate are displayed on each dot plot; for elimination efficiency calculations (**Methods),** raw cell counts are adjusted using the number of added and recorded CountBright beads.

**Figure S2. CD4^IL10^ cell phenotype.** At the end of the 14-day expansion, CD4^IL10^ cells were stained with two separate panels for characteristic CD4^IL10^ cell surface proteins^5-8^. **A.** The gating strategy. **B.** Histograms showing surface protein expression on CD4^IL10^ cells from donor 2441 and **C.** from donor 072, both gated through CD3^+^CD4^+^ NGFR^+^ live single cells

**Figure S3. Single-cell RNA-seq experiment design and sorting of AML and CD4^IL10^ cells. A.** Design of the scRNA-seq experiment. Purified live CD4^IL10^ cells, sensitive AML, and resistant AML samples (n = 2 for each) were analyzed by scRNA-seq first at day 0 (at thaw for AML, at the end of expansion cycle for CD4^IL10^ cells), and then after 24h co-culture in 1:1 ratio; co-cultured cells were again purified for live cells before scRNA-seq using fluorescence-activated cell sorting (FACS). Data was generated in two independent experiments, each with all AML samples but with a different CD4^IL10^ donor. **B.** Flow cytometry plots of one resistant (top row; sample PATISD) and sensitive (bottom row; sample PARCEV) primary AML cell sample after co-culture with CD4^IL10^ cells for 24 hours at 1:1 ratio; gated through live single cells. Data shown are representative of two independent experiments.

**Figure S4. Identification of AML programs in scRNA-seq data.** UMAP plots of AML single cells per AML patient and timepoint, colored by cluster (**Methods**). **B.** Cophenetic coefficients used for selecting the number of AML programs. The highest number of AML programs (5) for which the cophenetic coefficient was >0.99 was selected. **C.** Heatmap depicting the 5 initial AML programs identified, Rows - genes, columns - average expression of genes in sample-specific clusters, and colors - the relative expression of genes. **D.** The proportion of AML single cells assigned to each AML program, grouped by their mitochondrial read content. **E.** UMAP plots of AML single cells per AML patient and timepoint, colored by AML program (**Methods**).

**Figure S5. Analysis of AML transcriptional programs in bulk RNA-seq data, related to Figure 3. A.** Heatmap depicting the expression of AML program markers across hematopoietic cell lines, grouped by the dominant program (**Supplemental Methods**). **B.** Heatmap of the AML program abundances across cell lines from hematopoietic cancers. Rows – cell lines, columns – AML programs, colors – program abundance. **C.** The abundance of AML programs in selected cell lines. **D.** Heatmap depicting the expression of AML program markers across samples from the TARGET cohort, grouped by the dominant program. **E.** The –log_10_ *p*-values of the association of AML program abundance with overall survival (**left**) and relapse free survival (**right**) upon high-risk adjustment, calculated using a bivariate cox model with the AML program abundance and high-risk classification as covariates. High risk was identified if at least one of the following were present: t(6;9) highly correlated with FLT3-ITD; t(4;11), t(6;11); t(10;11); monosomy 7; FLT3-ITD positive.

**Figure S6. Gene-set enrichment of CD4^IL10^ signatures with CD4 T cell subtypes, related to Figure 4. A.** Gene-set enrichment of the top 50 genes over-expressed in CD4^IL10^ after co-culture with sensitive and resistant AML, in gene expression signatures derived from naïve, resting memory, and activated memory CD4^+^ T cells (**Supplemental Methods**). **B-C.** Bubble plots representing the potential cell-cell interactions between AML cells in program P3 (**B**) and P4 (**C**) and CD4^IL10^ cells cultured with resistant AML (**Methods**). Bubble sizes are proportional to the average fraction of single cells in each population expressing the genes encoding the first and second interaction partners. Colors indicate the average log_2_ fold change of genes encoding the interaction partners in the indicted AML program relative to other AML cells and in the CD4^IL10^ cells after culture with resistant AML. **D.** Expression of *ICAM1* in four primary AML samples analyzed by scRNA-seq across cells enriched in programs P1-P4. **E-F.** Expression of *ITGB2* (CD18), *ITGAL* (CD11a) and *ICAM1* (CD54) in: (**E**) CD4^IL10^ single cells and (**F**) CD4^IL10^ cells after the expansion cycle (resting state) in an independent, previously published bulk RNA-seq dataset^17^.

**Figure S7. The effects of ICAM1 knock-out and ICAM1/LFA-1 interaction inhibitors on T cell degranulation and killing. A.** Flow cytometry surface staining of U937 cells (unstained: pink, wild-type: purple, knock-out: orange) for TNF-α receptor (encoded by *TNFRSF1B*), IFN-γ receptor (*IFNGR1*), CD18 subunit of LFA-1 (*ITGB2*), and CD54 adhesion protein (*ICAM1*), minimum 5 days post genome editing. HEK293FT cells, which do not express TNF-α receptor, were used as a negative control (green). **B.** Representative flow cytometry plots of CD8^+^ T cells (n = 7) co-cultured alone, with PMA+I or with K562, for 5hr to detect degranulation, expressed as % CD8^+^CD107a^+^ cells within live singlet CD3^+^ T cells. PMA+I (phorbol 12-myristate 13-acetate + ionomycin) was a positive control for degranulation. Summary of control conditions for degranulation are shown in the bottom-right insert. **C.** Representative FACS plots from one CD4^IL10^ donor co-cultured 24 hr with U937 WT cells at 1:1 ratio in the presence of indicated LFA-1 inhibitors at concentrations indicated on each plot; numbers indicate percentage in AML gate. DMSO alone is used as vehicle control. **D.** Representative FACS plots from one CD8^+^ T cell donor 24hr after interaction with indicated target cells. Activation was measured as percent CD69^+^CD25^+/-^ CD8^+^ T cells.

**Figure S8. Expression of ICAM1 and CD64 on cell lines and primary AML cells**. **A**. Flow cytometry surface staining for high-affinity Fc receptor CD64 and ICAM1 (encoding CD54) on U937 wild-type cells (U937), U937 ICAM1 knock out cells (CD54ko), and on four primary AML samples (adult, SU540 and SU555; pediatric, PAPVET and PATLIG), depicted as histograms of specific stainings (blue) overlayed over each cell type’s unstained control (red). Numbers on histograms indicate the geometric mean fluorescence intensity (gMFI) ratio between specific marker and unstained control. **B.** Cumulative gMFI values from A.

**Figure S9. Imaging of U937 *in vivo* tumor progression.** Images taken on days 5, 8 and 12 post PBS, U937-WT-Luc^+^ or U937-*ICAM1-KO*-Luc^+^ injections are shown.
