# Supplementary material for: AML/T cell interactomics uncover correlates of patient outcomes and the key role of ICAM1 in T cell killing of AML
Source: Leukemia. 2024 May 9;38(6):1246–55. doi: 10.1038/s41375-024-02255-1 (PMC11147760; doi:10.1038/s41375-024-02255-1)
Supplement: Supplementary file 3 — Supplemental Material [file 41375_2024_2255_MOESM3_ESM.pdf]

Figure S1

A

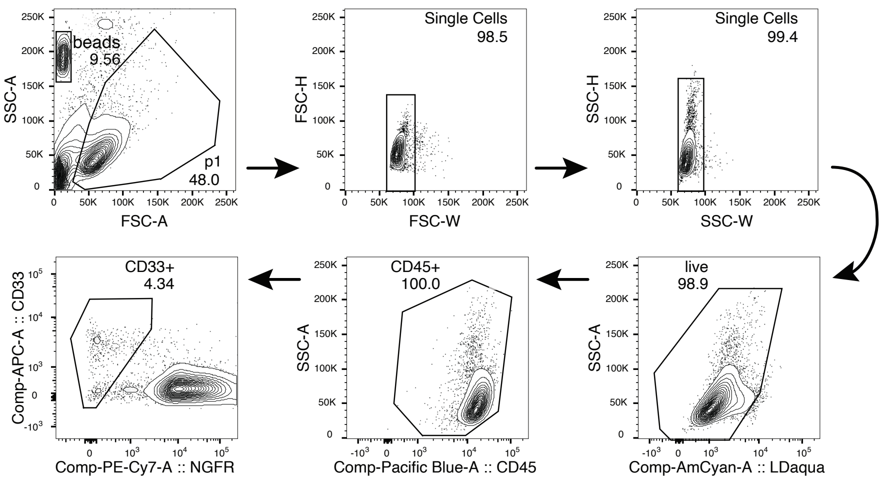

beads
9.56
p1
48.0
SSC-A
FSC-A
Single Cells
98.5
FSC-H
FSC-W
Single Cells
99.4
SSC-H
SSC-W
live
98.9
SSC-A
Comp-AmCyan-A :: LDaqua
CD45+
100.0
SSC-A
Comp-Pacific Blue-A :: CD45
CD33+
4.34
Comp-APC-A :: CD33
Comp-PE-Cy7-A :: NGFR

B

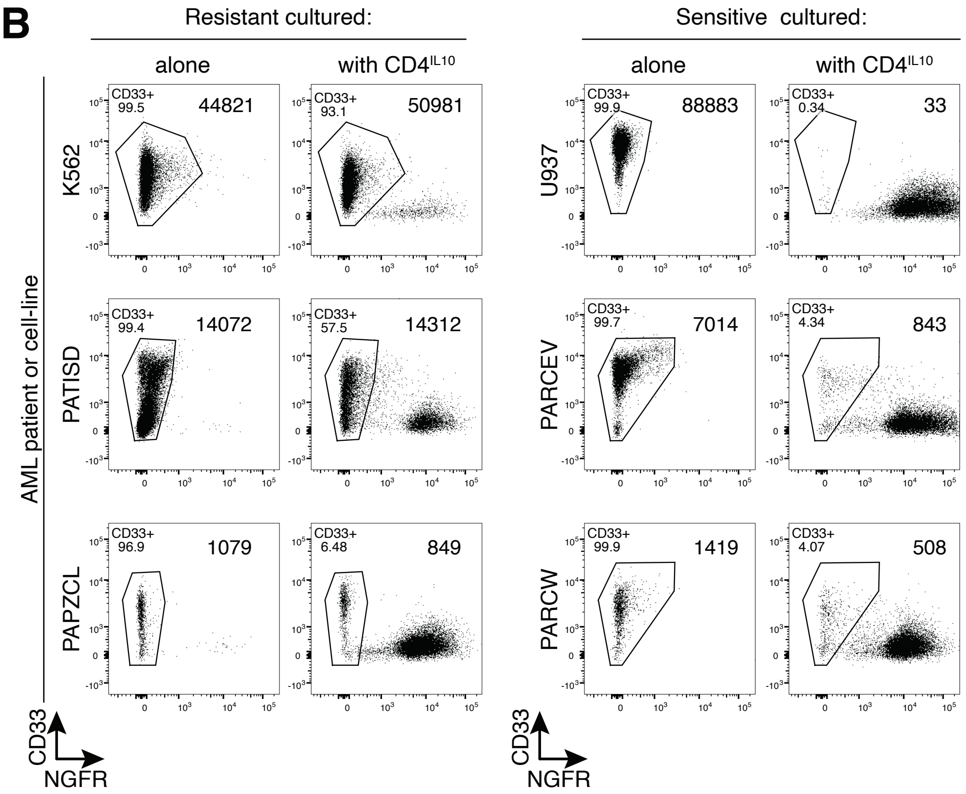

Resistant cultured:
alone
with CD4IL10
Sensitive cultured:
alone
with CD4IL10
AML patient or cell-line
K562
CD33+ 99.5
44821
CD33+ 93.1
50981
U937
CD33+ 99.9
88883
CD33+ 0.34
33
PATISD
CD33+ 99.4
14072
CD33+ 57.5
14312
PARCEV
CD33+ 99.7
7014
CD33+ 4.34
843
PAPZCL
CD33+ 96.9
1079
CD33+ 6.48
849
PARCW
CD33+ 99.9
1419
CD33+ 4.07
508
CD33
NGFR
CD33
NGFR

Figure S2

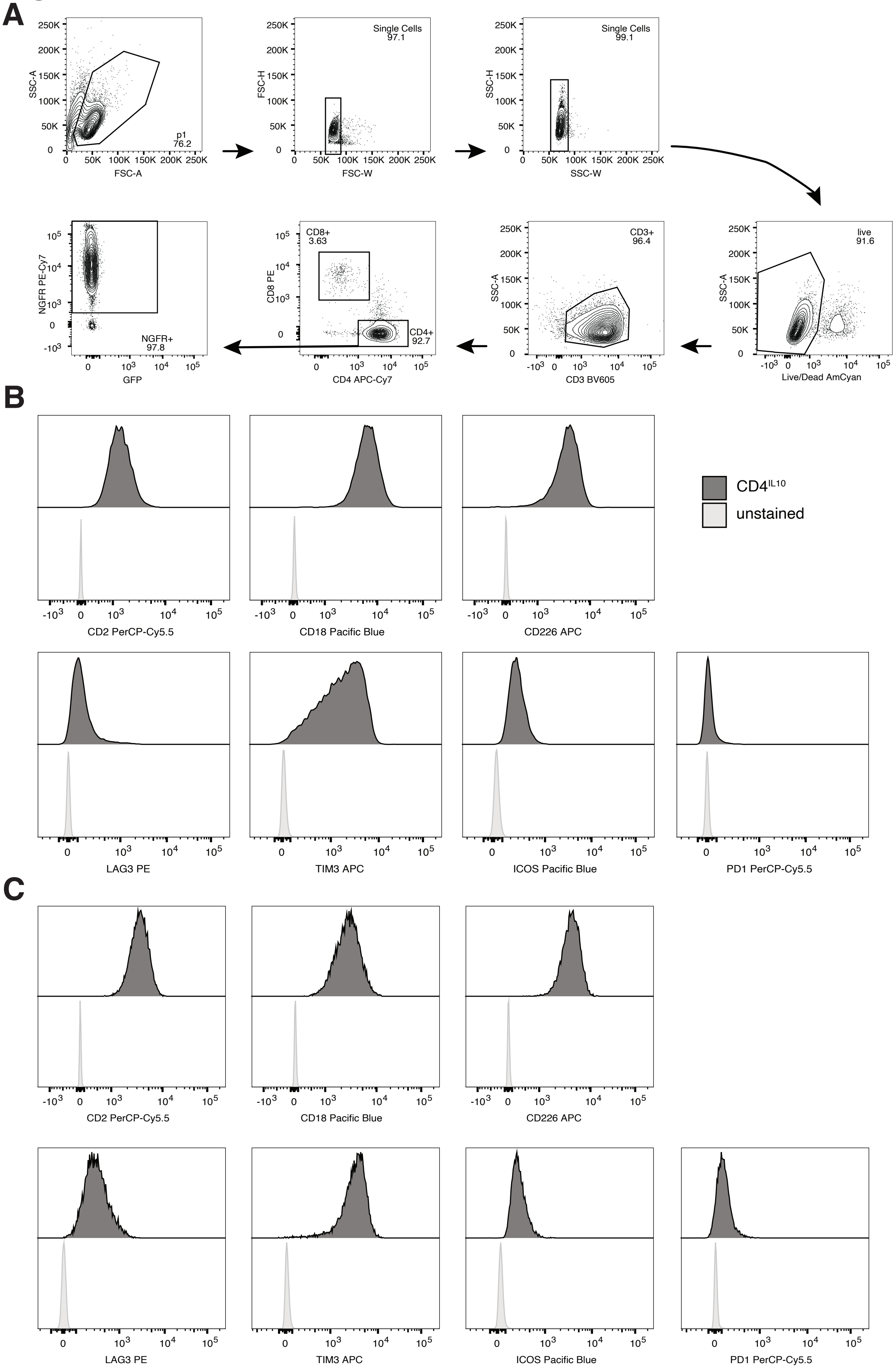
A
p1
76.2
Single Cells
97.1
Single Cells
99.1
SSC-A
FSC-A
FSC-H
FSC-W
SSC-H
SSC-W
live
91.6
Live/Dead AmCyan
CD3+
96.4
CD3 BV605
CD8+
3.63
CD4+
92.7
CD8 PE
CD4 APC-Cy7
NGFR+
97.8
NGFR PE-Cy7
GFP
B
CD4IL10
unstained
CD2 PerCP-Cy5.5
CD18 Pacific Blue
CD226 APC
LAG3 PE
TIM3 APC
ICOS Pacific Blue
PD1 PerCP-Cy5.5
C
CD2 PerCP-Cy5.5
CD18 Pacific Blue
CD226 APC
LAG3 PE
TIM3 APC
ICOS Pacific Blue
PD1 PerCP-Cy5.5

Figure S3

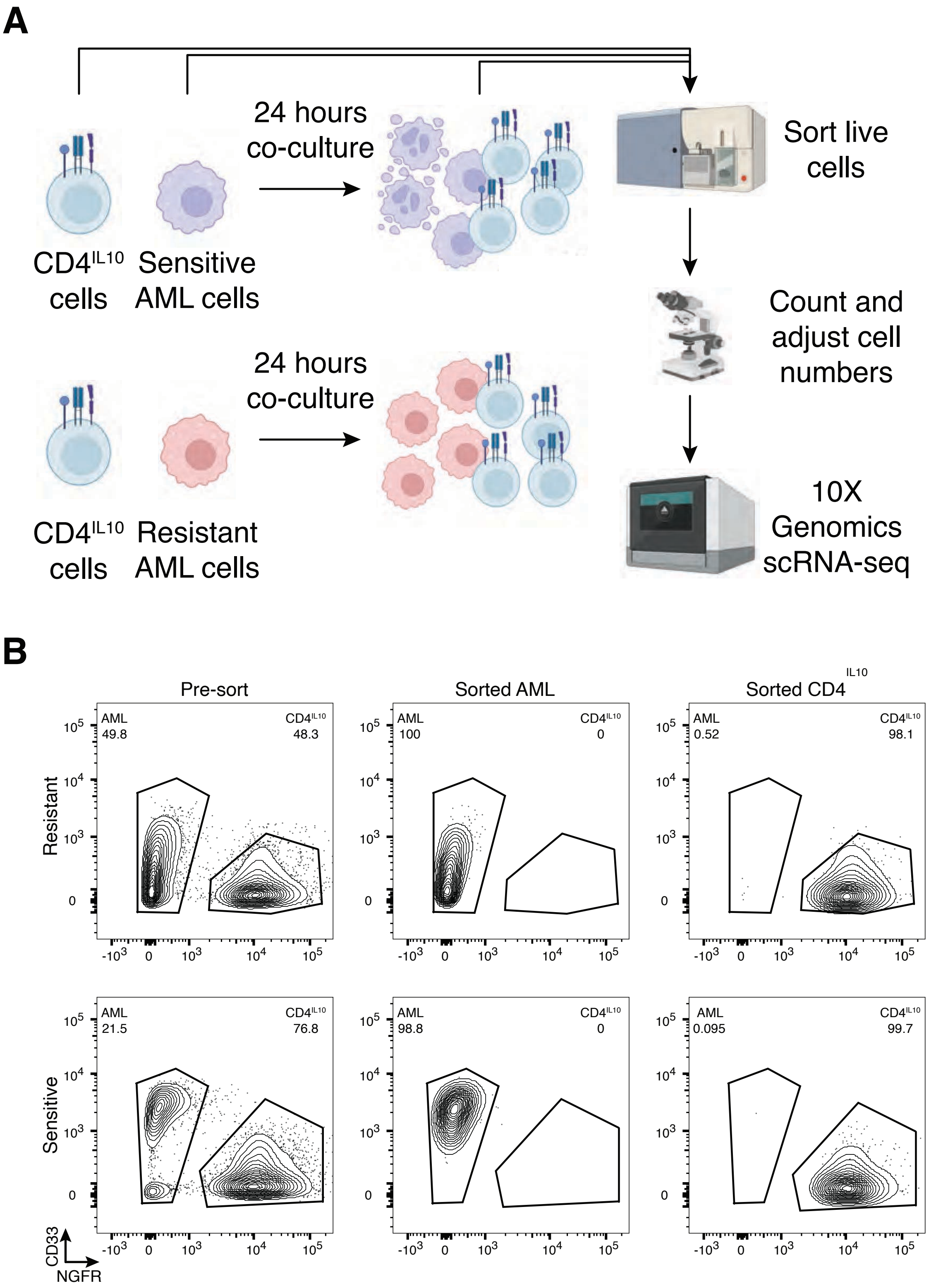
A
24 hours co-culture
Sort live cells
CD4^IL10 cells
Sensitive AML cells
Count and adjust cell numbers
24 hours co-culture
CD4^IL10 cells
Resistant AML cells
10X Genomics scRNA-seq
B
Pre-sort
Sorted AML
Sorted CD4^IL10
Resistant
AML 49.8
CD4^IL10 48.3
AML 100
CD4^IL10 0
AML 0.52
CD4^IL10 98.1
Sensitive
AML 21.5
CD4^IL10 76.8
AML 98.8
CD4^IL10 0
AML 0.095
CD4^IL10 99.7
CD33
NGFR

Figure S4

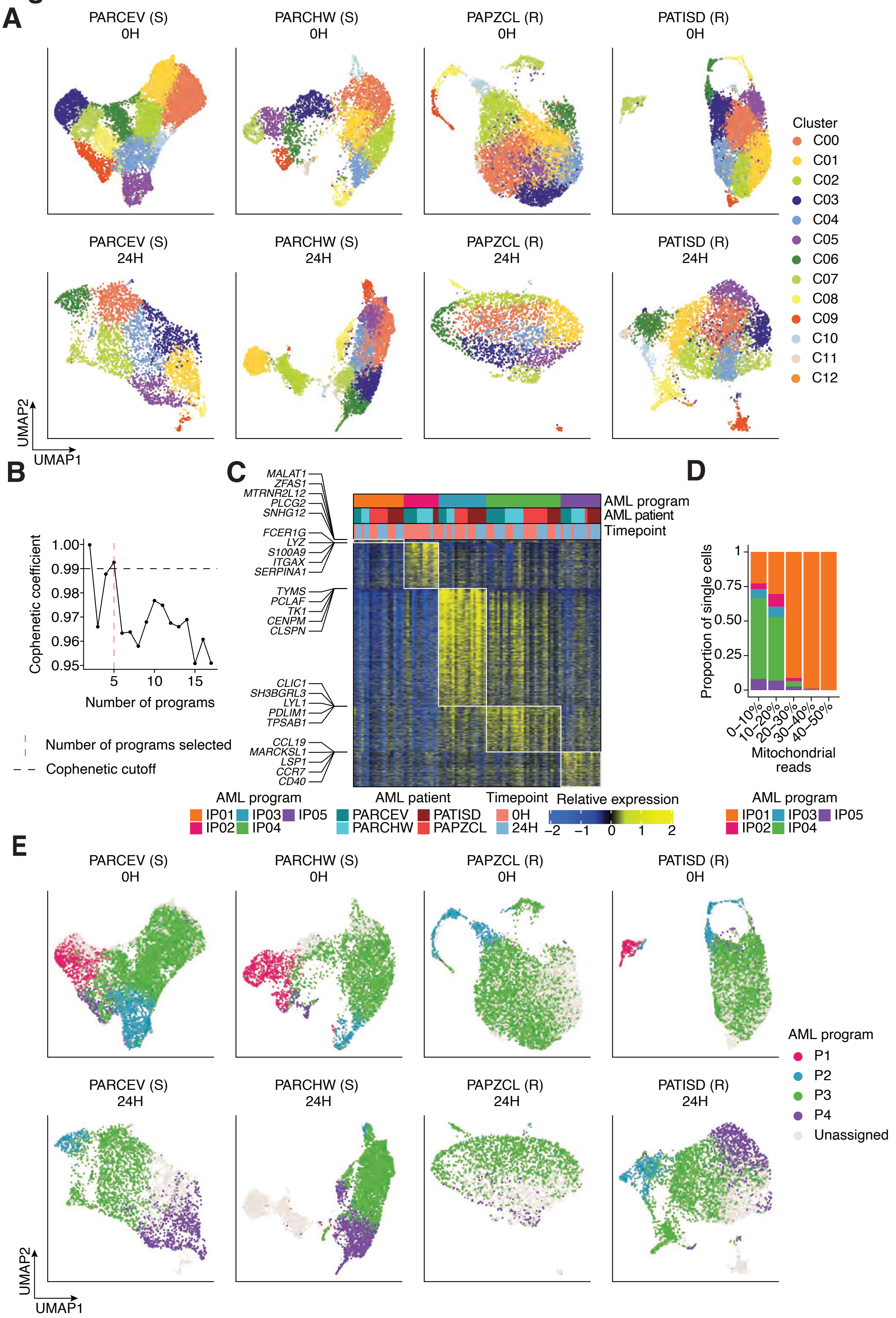
A
PARCEV (S) 0H
PARCHW (S) 0H
PAPZCL (R) 0H
PATISD (R) 0H
PARCEV (S) 24H
PARCHW (S) 24H
PAPZCL (R) 24H
PATISD (R) 24H
Cluster
C00
C01
C02
C03
C04
C05
C06
C07
C08
C09
C10
C11
C12
UMAP2
UMAP1
B
Cophenetic coefficient
1.00
0.99
0.98
0.97
0.96
0.95
5
10
15
Number of programs
Number of programs selected
Cophenetic cutoff
C
MALAT1
ZFAS1
MTRNR2L12
PLCG2
SNHG12
FCER1G
LYZ
S100A9
ITGAX
SERPINA1
TYMS
PCLAF
TK1
CENPM
CLSPN
CLIC1
SH3BGRL3
LYL1
PDLIM1
TPSAB1
CCL19
MARCKSL1
LSP1
CCR7
CD40
AML program
AML patient
Timepoint
AML program
IP01 IP03 IP05
IP02 IP04
AML patient
PARCEV PATISD
PARCHW PAPZCL
Timepoint
0H
24H
Relative expression
−2 −1 0 1 2
D
Proportion of single cells
1
0.75
0.50
0.25
0
0–10%
10–20%
20–30%
30–40%
40–50%
Mitochondrial reads
AML program
IP01 IP03 IP05
IP02 IP04
E
PARCEV (S) 0H
PARCHW (S) 0H
PAPZCL (R) 0H
PATISD (R) 0H
PARCEV (S) 24H
PARCHW (S) 24H
PAPZCL (R) 24H
PATISD (R) 24H
AML program
P1
P2
P3
P4
Unassigned
UMAP2
UMAP1

Figure S5

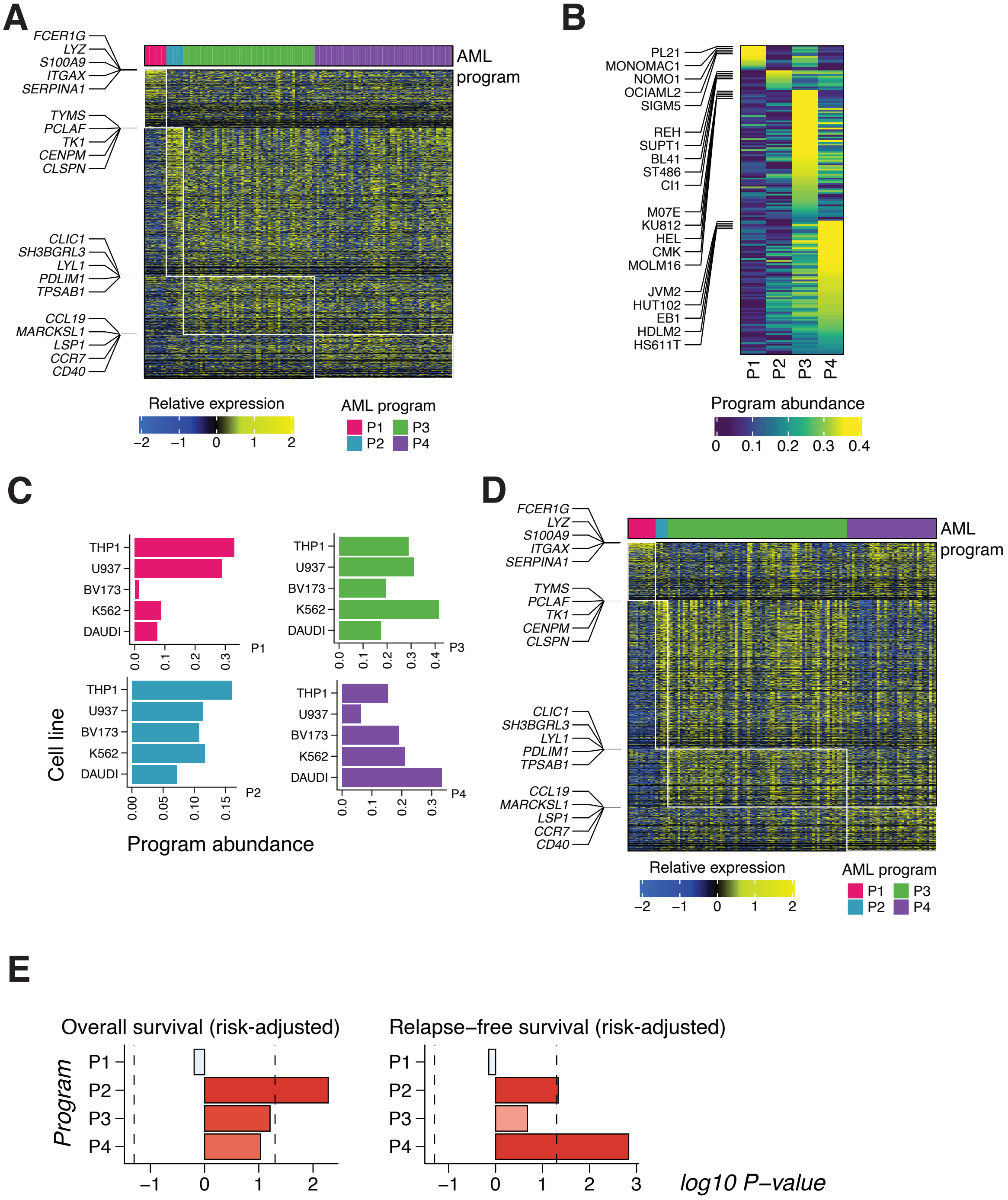

A
FCER1G
LYZ
S100A9
ITGAX
SERPINA1
TYMS
PCLAF
TK1
CENPM
CLSPN
CLIC1
SH3BGRL3
LYL1
PDLIM1
TPSAB1
CCL19
MARCKSL1
LSP1
CCR7
CD40
AML program
Relative expression
−2 −1 0 1 2
P1 P2 P3 P4
B
PL21
MONOMAC1
NOMO1
OCIAML2
SIGM5
REH
SUPT1
BL41
ST486
CI1
M07E
KU812
HEL
CMK
MOLM16
JVM2
HUT102
EB1
HDLM2
HS611T
P1 P2 P3 P4
Program abundance
0 0.1 0.2 0.3 0.4
C
THP1
U937
BV173
K562
DAUDI
Cell line
Program abundance
D
E
Overall survival (risk-adjusted)
Relapse−free survival (risk-adjusted)
Program
log10 P−value

Figure S6

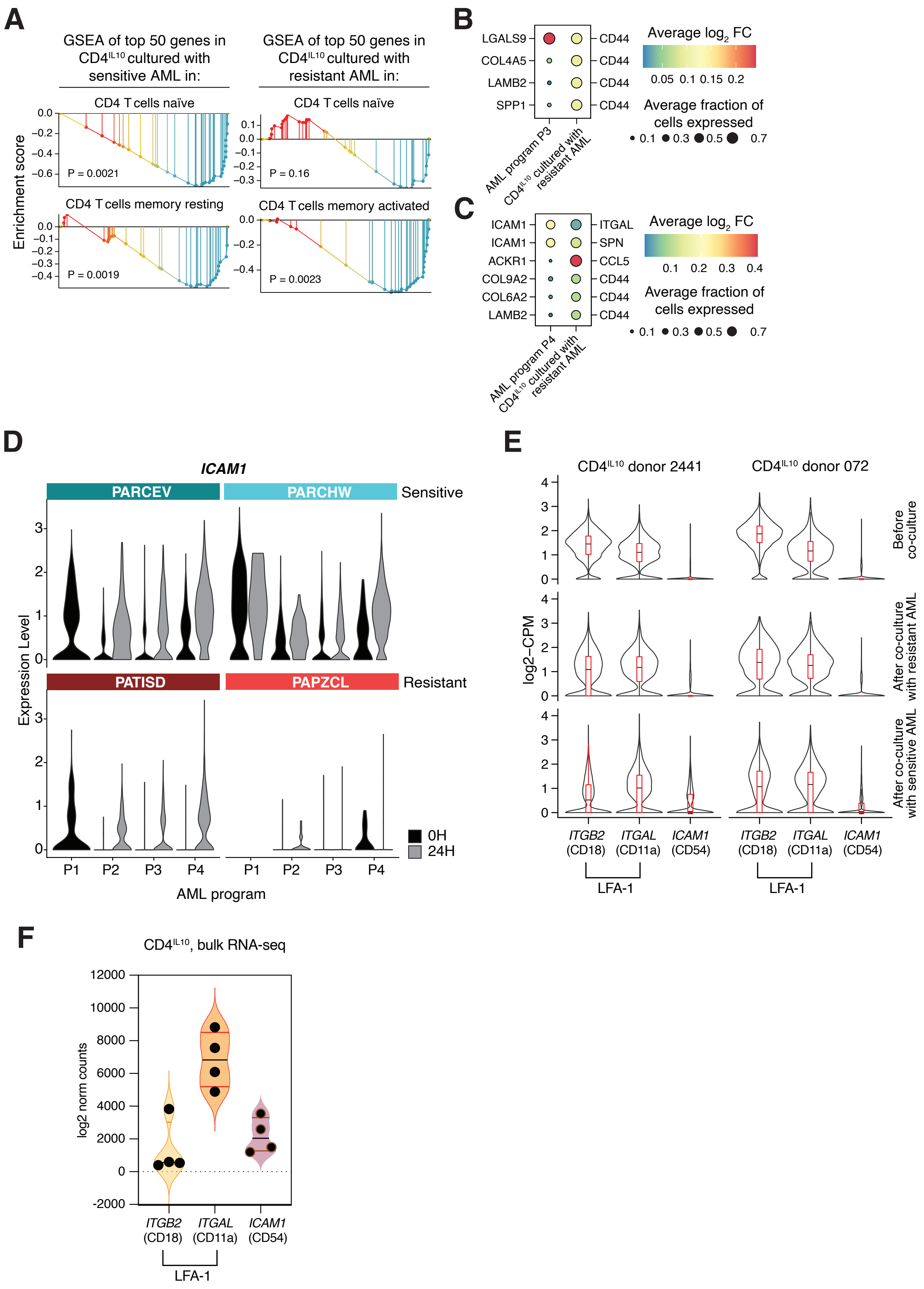

A
GSEA of top 50 genes in CD4IL10 cultured with sensitive AML in:
GSEA of top 50 genes in CD4IL10 cultured with resistant AML in:
CD4 T cells naïve
P = 0.0021
P = 0.16
CD4 T cells memory resting
CD4 T cells memory activated
P = 0.0019
P = 0.0023
Enrichment score
B
LGALS9
COL4A5
LAMB2
SPP1
CD44
AML program P3
CD4IL10 cultured with resistant AML
Average log2 FC
0.05 0.1 0.15 0.2
Average fraction of cells expressed
0.1 0.3 0.5 0.7
C
ICAM1
ACKR1
COL9A2
COL6A2
LAMB2
ITGAL
SPN
CCL5
CD44
AML program P4
CD4IL10 cultured with resistant AML
Average log2 FC
0.1 0.2 0.3 0.4
Average fraction of cells expressed
0.1 0.3 0.5 0.7
D
ICAM1
PARCEV
PARCHW
Sensitive
PATISD
PAPZCL
Resistant
Expression Level
0H
24H
P1 P2 P3 P4
AML program
E
CD4IL10 donor 2441
CD4IL10 donor 072
log2−CPM
Before co-culture
After co-culture with resistant AML
After co-culture with sensitive AML
ITGB2 (CD18)
ITGAL (CD11a)
ICAM1 (CD54)
LFA-1
F
CD4IL10, bulk RNA-seq
log2 norm counts
12000
10000
8000
6000
4000
2000
0
-2000
ITGB2 (CD18)
ITGAL (CD11a)
ICAM1 (CD54)
LFA-1

Figure S7

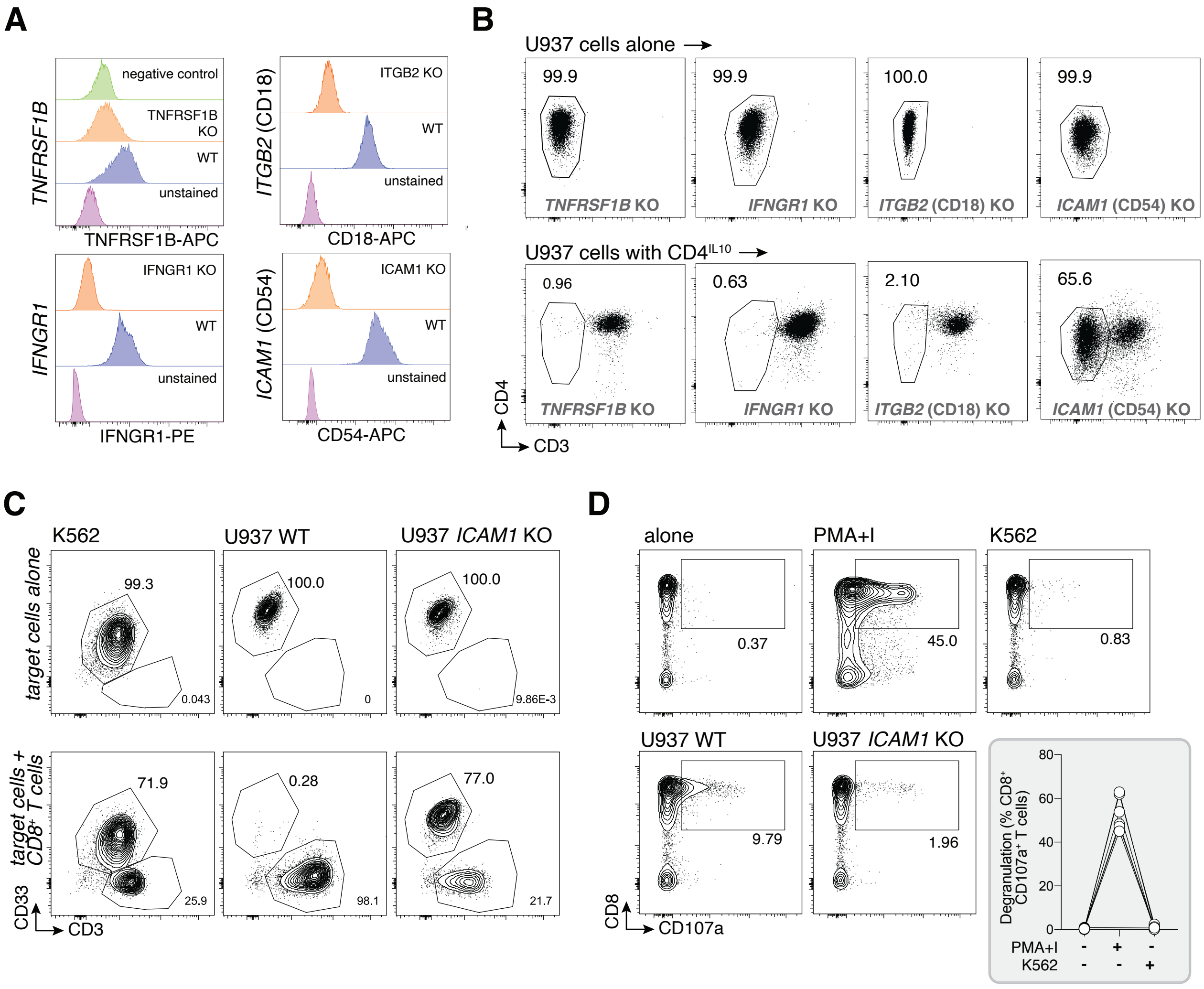
A
negative control
TNFRSF1B KO
WT
unstained
TNFRSF1B
TNFRSF1B-APC
ITGB2 KO
ITGB2 (CD18)
CD18-APC
IFNGR1 KO
IFNGR1
IFNGR1-PE
ICAM1 KO
ICAM1 (CD54)
CD54-APC
B
U937 cells alone
99.9
99.9
100.0
99.9
TNFRSF1B KO
IFNGR1 KO
ITGB2 (CD18) KO
ICAM1 (CD54) KO
U937 cells with CD4IL10
0.96
0.63
2.10
65.6
CD4
CD3
C
K562
U937 WT
U937 ICAM1 KO
target cells alone
99.3
100.0
100.0
0.043
0
9.86E-3
target cells + CD8+ T cells
71.9
0.28
77.0
25.9
98.1
21.7
CD33
CD3
D
alone
PMA+I
K562
0.37
45.0
0.83
U937 WT
U937 ICAM1 KO
9.79
1.96
CD8
CD107a
Degranulation (% CD8+ CD107a+ T cells)
80
60
40
20
0
PMA+I
K562

Figure S8

A

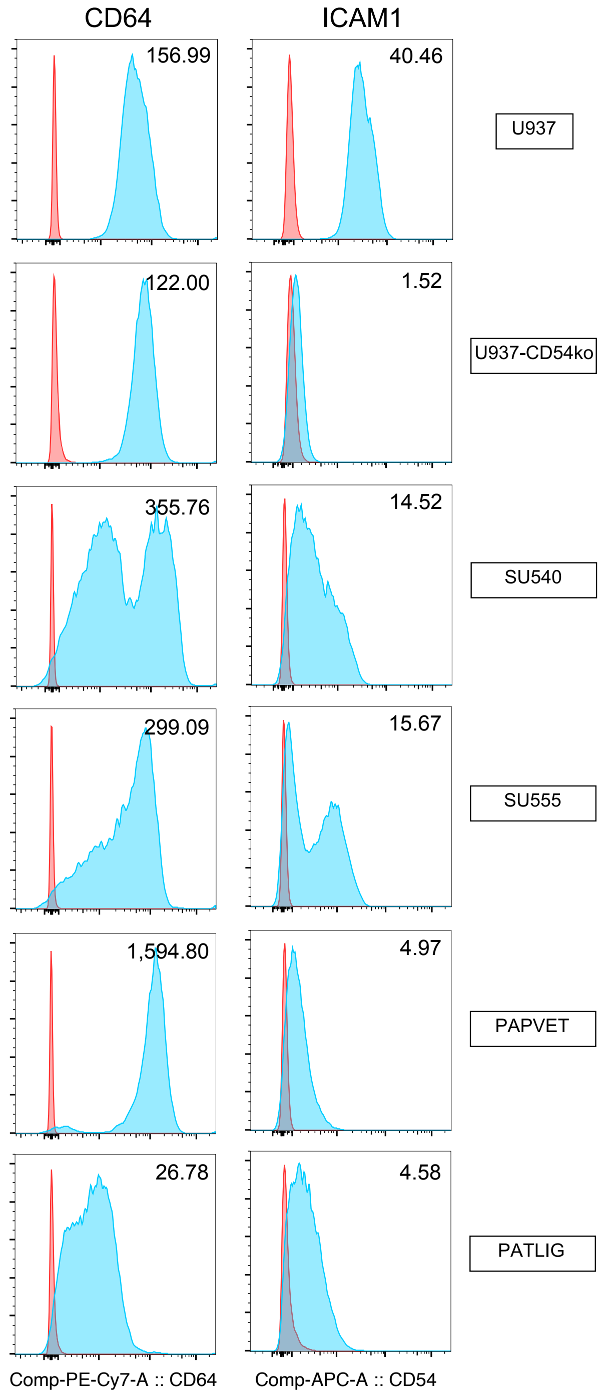
CD64
ICAM1
156.99
40.46
U937
122.00
1.52
U937-CD54ko
355.76
14.52
SU540
299.09
15.67
SU555
1,594.80
4.97
PAPVET
26.78
4.58
PATLIG
Comp-PE-Cy7-A :: CD64
Comp-APC-A :: CD54

B

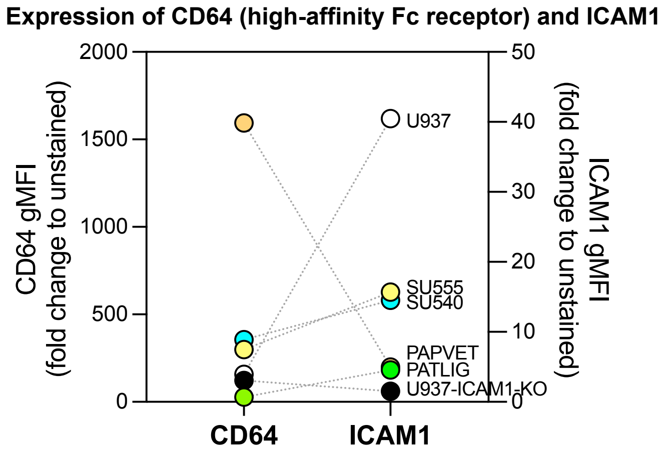
Expression of CD64 (high-affinity Fc receptor) and ICAM1
CD64 gMFI
(fold change to unstained)
ICAM1 gMFI
(fold change to unstained)
2000
1500
1000
500
0
50
40
30
20
10
0
U937
SU555
SU540
PAPVET
PATLIG
U937-ICAM1-KO
CD64
ICAM1

Figure S9

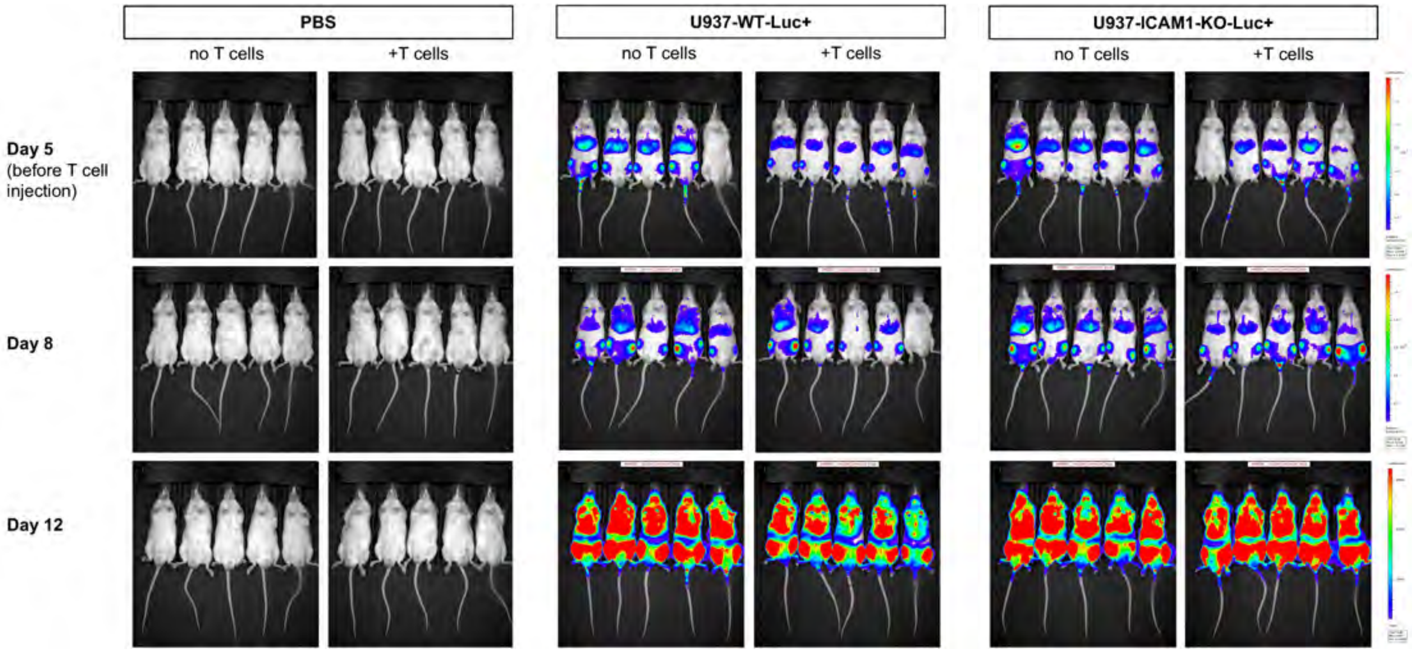

PBS
U937-WT-Luc+
U937-ICAM1-KO-Luc+
no T cells
+T cells
no T cells
+T cells
no T cells
+T cells
Day 5
(before T cell injection)
Day 8
Day 12

Table S1. Clinical data related to primary AML patient samples

AML patient	Response to CD4^{IL10} killing	FAB subtype	WHO classification	Cytogenetics onset	Blasts in bone marrow (%)	Age at diagnosis (days)	Risk group
PARCEV	Sensitive	M4	acute myelomonocytic leukemia	47,XX,+8(12)	94	4152	High
PARCHW	Sensitive	Unknown	AML with CBFB::MYH11 fusion	46,XY,inv(16)(p13.1q22)(17)/46,XY(3)	80	4209	Low
PATISD	Resistant	NOS	AML, myelodysplasia-related	45,XY,t(3;3)(q21;q26),-7(20)	91	3901	High
PAPZCL	Resistant	M0	AML, myelodysplasia-related	46,XY,del(5)(q13q31)(13)/46,XY(7)	97	5439	High

Legend: AML = acute myeloid leukemia; FAB = French-American-British classification of AML; WHO = World Health Organization classification of AML, 2022 update

Table S2. List of antibodies used.

Antibody	Clone	Company	Dilution	Panel		
				phenotyping	killing assay	sorting
CD2-PerCP-Cy5.5	RPA-2.10	Biolegend	1:50	+		
CD3-BV605 or PerCP-Cy5.5	OKT3	Biolegend	1:100	+	+	
CD4-APC-Cy7	RPA-T4	Biolegend	1:100	+	+	
CD8-PE-Cy7	SK1	BD Biosciences	1:100		+	
CD8-PE	SK1	BD Biosciences	1:100	+		
CD18-BV421 or APC	6.7	BD Biosciences	1:50	+		
CD25-AF700	BC96	Biolegend	1:100	+		
CD33-APC	AC104.3E3	Miltenyi Biotec	1:50		+	+
CD45-Pacific Blue	HI100	Biolegend	1:100		+	
CD54-APC	15.2	Tonbo Biosciences	1:50	+		
CD64-PE-Cy7	10.1	BD Biosciences	1:50	+		
CD226-APC	11A8	Biolegend	1:50	+		
CD271 (NGFR)-PE-Cy7	ME20.4	Biolegend	1:100	+	+	+
LAG3-PE	REA351	Miltenyi Biotec	1:33	+		
TIM3-AF647	7D3	BD Biosciences	1:50	+		
ICOS-Pacific Blue	C398.4A	Biolegend	1:50	+		
PD1-PerCP-Cy5.5	EH12.2H7	Biolegend	1:50	+		
Ghost dye violet 510 live/dead dye		Tonbo Biosciences	1:250	+	+	+
IFNGR1-PE	GIR-208	Biolegend	1:50	+		
TNFRSF1B-APC	3G7A02	Biolegend	1:50	+		
CD69-BV605	FN50	Biolegend	1:100	+		

Table S3. Genes differentially expressed in AML programs, related to **Figure 1**.

Gene symbol -= The symbol of gene for which the differential expression is assessed.
AML program = The AML program in which differential expression is assessed.
Average log2 fold change =
The weighted average LOG2 fold change of gene in cells assigned to The program indicated in column B relative to all The other cells, across all AML patient/timepoint pairs.
Avg. percent of cells assigned to program with CPM > 0 - The weighted average percentage of cells assigned to the program with CPM > 0, across all AML patient/timepoint pairs.
Avg. percent of cells not assigned to program with CPM > 0 =
The weighted average percentage of cells not assigned to the program with CPM > 0, across all AML patient/timepoint pairs.
DE meta z-score =
The meta z-score obtained by aggregating individual z-score statistics of differential expression (DE), across all AML patient/timepoint pairs, using Liptak's method (Methods).
DE *p*-value - The value obtained by converting the value in column **DE meta z-score** to a two-sided *p*-value.
DE *q*-value - The *q*-value corresponding to the *p*-value in column **DE *p*-value** after adjusted for multiple hypothesis testing using Benjamini-Hochberg method.
Suface protein - Flag indicating whether the gene is a surface protein, as annotated in Cell Surface Protein Atlas (*Bausch-Fluck et al. 2018*)**.**

Note: For columns C-F, the the natural log of the number of cells assigned to the program was used as weight.

Gene symbol	AML program	Average $\log_2$ fold change	Avg. percent of cells assigned to program with CPM > 0	Avg. percent of cells not assigned to program with CPM > 0	DE meta z-score	DE *p*-value	DE *q*-value	Surface protein
CD68	P1	1.15	0.91	0.38	37.93	0.000	0.000	TRUE
TREM1	P1	0.93	0.75	0.17	36.37	0.000	0.000	TRUE
GPR183	P1	0.92	0.80	0.39	28.73	0.000	0.000	TRUE
PLAUR	P1	0.91	0.87	0.59	26.50	0.000	0.000	TRUE
EVI2B	P1	0.90	0.89	0.64	31.40	0.000	0.000	TRUE
FCGRT	P1	0.84	0.82	0.33	31.53	0.000	0.000	TRUE
EMP3	P1	0.84	0.99	0.88	27.69	0.000	0.000	TRUE
TNFRSF1B	P1	0.82	0.65	0.25	22.74	0.000	0.000	TRUE
ITGB2	P1	0.81	0.90	0.61	30.09	0.000	0.000	TRUE
MS4A6A	P1	0.80	0.60	0.08	32.06	0.000	0.000	TRUE
CYBB	P1	0.79	0.62	0.09	32.11	0.000	0.000	TRUE
CD83	P1	0.79	0.85	0.63	24.62	0.000	0.000	TRUE
CLEC7A	P1	0.77	0.55	0.08	30.46	0.000	0.000	TRUE
ITGAX	P1	0.68	0.58	0.10	29.07	0.000	0.000	TRUE
EMP1	P1	0.67	0.83	0.66	20.92	0.000	0.000	TRUE
CD44	P1	0.67	0.99	0.92	20.86	0.000	0.000	TRUE
IL10RA	P1	0.67	0.69	0.20	29.64	0.000	0.000	TRUE
MPEG1	P1	0.66	0.57	0.06	32.97	0.000	0.000	TRUE
APLP2	P1	0.66	0.93	0.82	23.09	0.000	0.000	TRUE
CD14	P1	0.60	0.43	0.02	28.44	0.000	0.000	TRUE
PTAFR	P1	0.57	0.59	0.16	27.27	0.000	0.000	TRUE
HBEGF	P1	0.55	0.72	0.43	20.81	0.000	0.000	TRUE
HCAR3	P1	0.54	0.57	0.15	23.87	0.000	0.000	TRUE
SLC11A1	P1	0.54	0.45	0.06	26.04	0.000	0.000	TRUE
ICAM1	P1	0.53	0.63	0.35	20.09	0.000	0.000	TRUE
TLR2	P1	0.52	0.55	0.16	25.33	0.000	0.000	TRUE
CPD	P1	0.52	0.69	0.34	21.43	0.000	0.000	TRUE
ATP13A3	P1	0.51	0.72	0.51	16.97	0.000	0.000	TRUE
CD93	P1	0.49	0.44	0.06	24.33	0.000	0.000	TRUE
PILRA	P1	0.48	0.50	0.06	27.20	0.000	0.000	TRUE
RELT	P1	0.46	0.64	0.34	21.45	0.000	0.000	TRUE
IFNGR2	P1	0.46	0.63	0.36	20.09	0.000	0.000	TRUE
LRRC25	P1	0.46	0.48	0.04	28.31	0.000	0.000	TRUE
C5AR1	P1	0.45	0.32	0.01	23.68	0.000	0.000	TRUE
ITGA5	P1	0.44	0.70	0.45	19.40	0.000	0.000	TRUE
IGSF6	P1	0.44	0.46	0.09	21.03	0.000	0.000	TRUE
CD300E	P1	0.44	0.31	0.01	24.52	0.000	0.000	TRUE
ADAM17	P1	0.44	0.77	0.60	17.67	0.000	0.000	TRUE
SLC15A3	P1	0.43	0.46	0.05	25.21	0.000	0.000	TRUE
EVI2A	P1	0.43	0.61	0.34	17.16	0.000	0.000	TRUE
P2RX4	P1	0.42	0.61	0.35	17.76	0.000	0.000	TRUE
CD48	P1	0.40	0.53	0.38	12.58	0.000	0.000	TRUE
IL13RA1	P1	0.39	0.50	0.15	20.60	0.000	0.000	TRUE
IL17RA	P1	0.39	0.55	0.23	18.73	0.000	0.000	TRUE
SLC2A3	P1	0.39	0.90	0.81	9.88	0.000	0.000	TRUE
CXCL16	P1	0.39	0.38	0.05	21.26	0.000	0.000	TRUE
HLA-B	P1	0.39	1.00	0.99	22.90	0.000	0.000	TRUE
CD36	P1	0.39	0.43	0.10	23.49	0.000	0.000	TRUE
EREG	P1	0.39	0.22	0.03	14.74	0.000	0.000	TRUE
TLR4	P1	0.38	0.38	0.03	25.08	0.000	0.000	TRUE
MYADM	P1	0.37	0.87	0.86	17.46	0.000	0.000	TRUE
ABCA1	P1	0.37	0.49	0.25	18.47	0.000	0.000	TRUE
PRNP	P1	0.36	0.71	0.53	17.48	0.000	0.000	TRUE
CD302	P1	0.35	0.69	0.58	15.95	0.000	0.000	TRUE
FCGR2A	P1	0.35	0.45	0.18	18.83	0.000	0.000	TRUE
PLXDC2	P1	0.35	0.51	0.24	18.06	0.000	0.000	TRUE
LILRB2	P1	0.35	0.34	0.02	22.92	0.000	0.000	TRUE
CD53	P1	0.35	0.73	0.61	12.87	0.000	0.000	TRUE
SLCO3A1	P1	0.35	0.56	0.36	17.21	0.000	0.000	TRUE
CCR1	P1	0.34	0.35	0.02	23.38	0.000	0.000	TRUE
SLC43A2	P1	0.34	0.40	0.15	16.51	0.000	0.000	TRUE
TCIRG1	P1	0.34	0.54	0.28	15.37	0.000	0.000	TRUE
ITGAM	P1	0.32	0.35	0.07	21.41	0.000	0.000	TRUE
CLEC5A	P1	0.32	0.29	0.04	20.14	0.000	0.000	TRUE
LRP1	P1	0.31	0.30	0.01	23.58	0.000	0.000	TRUE
ANPEP	P1	0.31	0.47	0.38	2.47	0.013	0.030	TRUE
CD55	P1	0.30	0.66	0.57	15.53	0.000	0.000	TRUE
HCAR2	P1	0.30	0.41	0.12	18.48	0.000	0.000	TRUE
PTGER2	P1	0.30	0.45	0.29	12.29	0.000	0.000	TRUE
CSF2RA	P1	0.29	0.44	0.15	17.58	0.000	0.000	TRUE
SIRPA	P1	0.29	0.40	0.13	18.75	0.000	0.000	TRUE
STAB1	P1	0.29	0.44	0.23	16.19	0.000	0.000	TRUE
QSOX1	P1	0.29	0.53	0.34	13.88	0.000	0.000	TRUE
ATP1A1	P1	0.28	0.63	0.46	13.07	0.000	0.000	TRUE
CLEC12A	P1	0.28	0.50	0.34	13.51	0.000	0.000	TRUE
LDLR	P1	0.28	0.68	0.63	11.81	0.000	0.000	TRUE
ADAM9	P1	0.28	0.49	0.26	13.25	0.000	0.000	TRUE
FCAR	P1	0.27	0.28	0.01	22.68	0.000	0.000	TRUE
BST1	P1	0.27	0.39	0.18	15.40	0.000	0.000	TRUE
ITGAL	P1	0.27	0.46	0.25	10.97	0.000	0.000	TRUE

CD86	P1	0.26	0.32	0.07	19.17	0.000	0.000	TRUE
TNFSF13B	P1	0.25	0.61	0.51	9.11	0.000	0.000	TRUE
P2RX7	P1	0.25	0.30	0.10	13.17	0.000	0.000	TRUE
LILRB1	P1	0.25	0.26	0.02	16.48	0.000	0.000	TRUE
SLC6A6	P1	0.25	0.51	0.36	10.51	0.000	0.000	TRUE
SLAMF8	P1	0.25	0.26	0.02	5.94	0.000	0.000	TRUE
LAMP1	P1	0.25	0.74	0.66	10.56	0.000	0.000	TRUE
FCGR1A	P1	0.24	0.35	0.12	14.41	0.000	0.000	TRUE
IFNGR1	P1	0.24	0.58	0.48	11.62	0.000	0.000	TRUE
CXCR4	P1	0.23	0.50	0.39	8.64	0.000	0.000	TRUE
NRP1	P1	0.23	0.23	0.07	4.78	0.000	0.000	TRUE
IL6R	P1	0.23	0.41	0.21	13.12	0.000	0.000	TRUE
RNF149	P1	0.22	0.46	0.35	6.71	0.000	0.000	TRUE
LILRB3	P1	0.22	0.26	0.05	14.12	0.000	0.000	TRUE
CSF1R	P1	0.22	0.30	0.09	15.63	0.000	0.000	TRUE
HM13	P1	0.21	0.67	0.59	7.45	0.000	0.000	TRUE
PTPRJ	P1	0.21	0.30	0.09	14.17	0.000	0.000	TRUE
ADAM8	P1	0.20	0.52	0.40	10.68	0.000	0.000	TRUE
AQP9	P1	0.20	0.15	0.00	15.37	0.000	0.000	TRUE
CD4	P1	0.19	0.27	0.09	14.28	0.000	0.000	TRUE
HRH2	P1	0.19	0.29	0.11	14.76	0.000	0.000	TRUE
RNF13	P1	0.19	0.33	0.23	7.37	0.000	0.000	TRUE
IGF2R	P1	0.18	0.24	0.06	12.39	0.000	0.000	TRUE
ITGB1	P1	0.18	0.66	0.60	8.04	0.000	0.000	TRUE
CD1D	P1	0.18	0.23	0.03	15.86	0.000	0.000	TRUE
IGFLR1	P1	0.18	0.42	0.31	8.76	0.000	0.000	TRUE
GPR132	P1	0.18	0.26	0.08	13.21	0.000	0.000	TRUE
MRC1	P1	0.18	0.19	0.05	9.72	0.000	0.000	TRUE
SLC31A1	P1	0.18	0.33	0.17	11.40	0.000	0.000	TRUE
ADORA2A	P1	0.18	0.25	0.08	8.87	0.000	0.000	TRUE
UNC93B1	P1	0.18	0.47	0.33	8.27	0.000	0.000	TRUE
MMP14	P1	0.18	0.15	0.07	2.60	0.009	0.021	TRUE
GPR35	P1	0.17	0.25	0.07	13.30	0.000	0.000	TRUE
PLXNB2	P1	0.17	0.41	0.28	9.87	0.000	0.000	TRUE
IL10RB	P1	0.17	0.35	0.21	9.09	0.000	0.000	TRUE
SLC30A1	P1	0.17	0.34	0.19	8.44	0.000	0.000	TRUE
MEGF9	P1	0.17	0.28	0.13	9.61	0.000	0.000	TRUE
P2RY13	P1	0.17	0.19	0.04	14.79	0.000	0.000	TRUE
LRP10	P1	0.16	0.34	0.26	4.95	0.000	0.000	TRUE
TLR8	P1	0.16	0.17	0.01	11.74	0.000	0.000	TRUE
TMED7	P1	0.16	0.53	0.44	6.38	0.000	0.000	TRUE
SIRPB1	P1	0.16	0.20	0.03	15.09	0.000	0.000	TRUE
PTPRC	P1	0.16	0.91	0.91	9.48	0.000	0.000	TRUE
TSPAN14	P1	0.16	0.27	0.10	11.39	0.000	0.000	TRUE
LILRA2	P1	0.16	0.23	0.06	14.65	0.000	0.000	TRUE
CSF3R	P1	0.16	0.67	0.65	7.74	0.000	0.000	TRUE
MFSD2A	P1	0.15	0.23	0.06	13.26	0.000	0.000	TRUE
NPTN	P1	0.15	0.33	0.20	6.33	0.000	0.000	TRUE
LILRA5	P1	0.15	0.15	0.01	14.33	0.000	0.000	TRUE
TMEM9B	P1	0.15	0.41	0.31	8.03	0.000	0.000	TRUE
HAVCR2	P1	0.15	0.31	0.16	9.70	0.000	0.000	TRUE
LILRA1	P1	0.15	0.18	0.02	12.52	0.000	0.000	TRUE
SECTM1	P1	0.14	0.18	0.04	11.37	0.000	0.000	TRUE
NOTCH2	P1	0.14	0.31	0.17	9.32	0.000	0.000	TRUE
CCRL2	P1	0.14	0.18	0.04	9.09	0.000	0.000	TRUE
PECAM1	P1	0.14	0.47	0.45	7.27	0.000	0.000	TRUE
NFAM1	P1	0.14	0.17	0.02	15.56	0.000	0.000	TRUE
SEMA4A	P1	0.14	0.42	0.30	7.67	0.000	0.000	TRUE
NPC1	P1	0.14	0.22	0.09	8.72	0.000	0.000	TRUE
THBD	P1	0.14	0.14	0.03	10.26	0.000	0.000	TRUE
ADRB2	P1	0.14	0.19	0.06	12.17	0.000	0.000	TRUE
SEMA6B	P1	0.13	0.22	0.08	10.36	0.000	0.000	TRUE
CD180	P1	0.13	0.19	0.07	10.81	0.000	0.000	TRUE
SERINC1	P1	0.13	0.58	0.55	4.30	0.000	0.000	TRUE
IL4R	P1	0.13	0.31	0.16	8.24	0.000	0.000	TRUE
SLC44A1	P1	0.13	0.38	0.34	7.32	0.000	0.000	TRUE
ADAM10	P1	0.13	0.55	0.51	4.80	0.000	0.000	TRUE
MYOF	P1	0.13	0.19	0.06	9.72	0.000	0.000	TRUE
ENTPD1	P1	0.13	0.18	0.04	14.71	0.000	0.000	TRUE
PRLR	P1	0.12	0.14	0.02	11.88	0.000	0.000	TRUE
TMX4	P1	0.12	0.54	0.55	7.37	0.000	0.000	TRUE
SORT1	P1	0.12	0.19	0.06	10.57	0.000	0.000	TRUE
CD163	P1	0.12	0.13	0.00	14.48	0.000	0.000	TRUE
SLC36A4	P1	0.12	0.30	0.21	6.04	0.000	0.000	TRUE
FCER1A	P1	0.12	0.14	0.05	8.71	0.000	0.000	TRUE
MSLN	P1	0.11	0.18	0.10	5.44	0.000	0.000	TRUE
C3AR1	P1	0.11	0.11	0.02	8.51	0.000	0.000	TRUE
UPK3A	P1	0.11	0.16	0.04	10.23	0.000	0.000	TRUE
PSEN1	P1	0.11	0.36	0.28	4.84	0.000	0.000	TRUE
CSF1	P1	0.11	0.17	0.09	9.47	0.000	0.000	TRUE
FCGR1B	P1	0.11	0.16	0.06	9.47	0.000	0.000	TRUE
CR1	P1	0.10	0.11	0.01	11.44	0.000	0.000	TRUE
LYPD2	P1	0.10	0.06	0.00	5.30	0.000	0.000	TRUE
SLC7A5	P1	0.10	0.35	0.28	5.27	0.000	0.000	TRUE
SLC24A4	P1	0.10	0.12	0.02	10.47	0.000	0.000	TRUE
MCOLN1	P1	0.10	0.19	0.11	8.52	0.000	0.000	TRUE
ADCY7	P1	0.10	0.21	0.10	9.40	0.000	0.000	TRUE
VNN2	P1	0.10	0.10	0.01	10.67	0.000	0.000	TRUE
FCGR2B	P1	0.10	0.11	0.03	8.25	0.000	0.000	TRUE
ANO6	P1	0.09	0.27	0.21	5.27	0.000	0.000	TRUE
SLC8A1	P1	0.09	0.12	0.02	11.20	0.000	0.000	TRUE
CD300LF	P1	0.09	0.19	0.11	6.41	0.000	0.000	TRUE
TTYH3	P1	0.09	0.23	0.17	6.51	0.000	0.000	TRUE
SIRPB2	P1	0.09	0.13	0.03	10.11	0.000	0.000	TRUE
CEACAM4	P1	0.08	0.17	0.09	5.33	0.000	0.000	TRUE
GPR84	P1	0.08	0.13	0.03	9.02	0.000	0.000	TRUE
CD300C	P1	0.08	0.12	0.02	11.52	0.000	0.000	TRUE
TLR1	P1	0.08	0.22	0.15	2.39	0.017	0.037	TRUE
SIGLEC10	P1	0.08	0.10	0.02	9.49	0.000	0.000	TRUE
PANX1	P1	0.08	0.16	0.07	3.37	0.001	0.002	TRUE
P2RY6	P1	0.08	0.11	0.00	6.69	0.000	0.000	TRUE

NRCAM	P1	0.08	0.08	0.01	2.29	0.022	0.047	TRUE
SLC8B1	P1	0.08	0.19	0.12	5.36	0.000	0.000	TRUE
OLR1	P1	0.08	0.10	0.01	9.15	0.000	0.000	TRUE
TMEM104	P1	0.08	0.14	0.07	6.80	0.000	0.000	TRUE
SLC11A2	P1	0.07	0.22	0.17	3.04	0.002	0.006	TRUE
UBAC2	P1	0.07	0.36	0.35	3.19	0.001	0.004	TRUE
MILR1	P1	0.07	0.17	0.11	4.08	0.000	0.000	TRUE
VSTM1	P1	0.07	0.13	0.09	4.17	0.000	0.000	TRUE
SIGLEC14	P1	0.07	0.09	0.01	10.74	0.000	0.000	TRUE
TNFRSF10A	P1	0.07	0.18	0.13	4.22	0.000	0.000	TRUE
MSR1	P1	0.07	0.07	0.00	10.14	0.000	0.000	TRUE
RECK	P1	0.07	0.09	0.07	3.97	0.000	0.000	TRUE
OSTM1	P1	0.07	0.15	0.10	4.41	0.000	0.000	TRUE
TPCN1	P1	0.06	0.11	0.06	6.01	0.000	0.000	TRUE
C5AR2	P1	0.06	0.08	0.01	10.62	0.000	0.000	TRUE
PLB1	P1	0.06	0.16	0.11	5.72	0.000	0.000	TRUE
FPR1	P1	0.06	0.06	0.00	8.27	0.000	0.000	TRUE
TMEM154	P1	0.06	0.14	0.09	3.70	0.000	0.001	TRUE
CPM	P1	0.06	0.11	0.03	7.35	0.000	0.000	TRUE
FURIN	P1	0.06	0.32	0.32	3.52	0.000	0.001	TRUE
VNN3	P1	0.06	0.06	0.00	10.31	0.000	0.000	TRUE
SLC9A1	P1	0.06	0.14	0.09	5.58	0.000	0.000	TRUE
MUC12	P1	0.06	0.10	0.04	6.53	0.000	0.000	TRUE
ATG9A	P1	0.06	0.13	0.08	5.04	0.000	0.000	TRUE
SERINC5	P1	0.06	0.13	0.08	3.86	0.000	0.000	TRUE
SLC36A1	P1	0.06	0.11	0.05	6.92	0.000	0.000	TRUE
CD276	P1	0.06	0.08	0.01	4.42	0.000	0.000	TRUE
ACVR1B	P1	0.06	0.18	0.12	4.72	0.000	0.000	TRUE
ASGR2	P1	0.06	0.08	0.02	8.65	0.000	0.000	TRUE
TMEM8A	P1	0.06	0.18	0.14	3.19	0.001	0.004	TRUE
FFAR2	P1	0.06	0.07	0.00	10.66	0.000	0.000	TRUE
ASGR1	P1	0.05	0.12	0.08	4.74	0.000	0.000	TRUE
TNFSF8	P1	0.05	0.08	0.03	4.94	0.000	0.000	TRUE
S1PR3	P1	0.05	0.06	0.01	8.28	0.000	0.000	TRUE
FCGR3A	P1	0.05	0.04	0.00	5.98	0.000	0.000	TRUE
NT5E	P1	0.05	0.07	0.06	-2.31	0.021	0.045	TRUE
CD22	P1	0.05	0.07	0.02	7.92	0.000	0.000	TRUE
GPBAR1	P1	0.05	0.07	0.02	6.34	0.000	0.000	TRUE
SLC12A9	P1	0.05	0.14	0.09	4.14	0.000	0.000	TRUE
SELPLG	P1	0.05	0.15	0.11	3.29	0.001	0.003	TRUE
OPN3	P1	0.05	0.13	0.07	5.44	0.000	0.000	TRUE
TLR6	P1	0.05	0.12	0.06	4.21	0.000	0.000	TRUE
TMEM63B	P1	0.05	0.10	0.06	6.57	0.000	0.000	TRUE
SLC22A15	P1	0.05	0.06	0.01	5.82	0.000	0.000	TRUE
P2RY2	P1	0.05	0.07	0.02	8.26	0.000	0.000	TRUE
GPR160	P1	0.05	0.10	0.06	3.45	0.001	0.002	TRUE
TMEM158	P1	0.05	0.06	0.03	2.68	0.007	0.017	TRUE
SIGLEC9	P1	0.04	0.06	0.00	8.51	0.000	0.000	TRUE
IL1R1	P1	0.04	0.05	0.01	8.01	0.000	0.000	TRUE
STS	P1	0.04	0.07	0.02	5.53	0.000	0.000	TRUE
HEG1	P1	0.04	0.08	0.02	3.17	0.002	0.004	TRUE
GPRC5A	P1	0.04	0.05	0.01	7.91	0.000	0.000	TRUE
SDC2	P1	0.04	0.06	0.00	7.94	0.000	0.000	TRUE
SLC2A9	P1	0.04	0.07	0.03	6.14	0.000	0.000	TRUE
SLCO4A1	P1	0.04	0.08	0.06	-4.21	0.000	0.000	TRUE
HRH1	P1	0.04	0.05	0.01	8.48	0.000	0.000	TRUE
TLR5	P1	0.04	0.05	0.00	8.48	0.000	0.000	TRUE
CDH23	P1	0.04	0.05	0.01	6.40	0.000	0.000	TRUE
FOLR2	P1	0.03	0.05	0.01	6.09	0.000	0.000	TRUE
GPR65	P1	0.03	0.06	0.03	5.21	0.000	0.000	TRUE
IL1R2	P1	0.03	0.04	0.00	6.34	0.000	0.000	TRUE
CCR5	P1	0.03	0.05	0.00	5.15	0.000	0.000	TRUE
LDLRAD3	P1	0.03	0.05	0.01	3.26	0.001	0.003	TRUE
TMEM8B	P1	0.03	0.06	0.03	4.75	0.000	0.000	TRUE
ABCC3	P1	0.03	0.04	0.00	6.81	0.000	0.000	TRUE
SLCO4C1	P1	0.03	0.04	0.02	5.16	0.000	0.000	TRUE
TLR7	P1	0.03	0.04	0.01	6.28	0.000	0.000	TRUE
SIGLEC7	P1	0.03	0.04	0.00	6.33	0.000	0.000	TRUE
SLC16A6	P1	0.03	0.03	0.00	6.35	0.000	0.000	TRUE
CD101	P1	0.03	0.04	0.01	6.29	0.000	0.000	TRUE
HTR7	P1	0.03	0.05	0.02	3.61	0.000	0.001	TRUE
EPHB2	P1	0.03	0.04	0.01	3.31	0.001	0.002	TRUE
CRTAM	P1	0.03	0.05	0.02	5.71	0.000	0.000	TRUE
SLC37A2	P1	0.03	0.07	0.04	2.58	0.010	0.022	TRUE
ANKH	P1	0.02	0.06	0.04	2.82	0.005	0.012	TRUE
GDPD5	P1	0.02	0.05	0.02	4.06	0.000	0.000	TRUE
SERINC2	P1	0.02	0.04	0.02	4.44	0.000	0.000	TRUE
CLEC12B	P1	0.02	0.04	0.01	3.55	0.000	0.001	TRUE
SIGLEC1	P1	0.02	0.04	0.01	6.22	0.000	0.000	TRUE
FPR2	P1	0.02	0.02	0.00	4.60	0.000	0.000	TRUE
DCSTAMP	P1	0.02	0.02	0.00	2.61	0.009	0.021	TRUE
CCR2	P1	0.02	0.03	0.00	6.54	0.000	0.000	TRUE
IL31RA	P1	0.02	0.03	0.00	4.83	0.000	0.000	TRUE
SIGLEC5	P1	0.02	0.04	0.01	4.26	0.000	0.000	TRUE
CD1B	P1	0.02	0.03	0.00	3.22	0.001	0.003	TRUE
GJB2	P1	0.02	0.03	0.01	2.98	0.003	0.007	TRUE
FPR3	P1	0.02	0.02	0.00	3.76	0.000	0.000	TRUE
CMKLR1	P1	0.02	0.02	0.00	3.31	0.001	0.002	TRUE
GPR141	P1	0.02	0.05	0.03	3.36	0.001	0.002	TRUE
TREM2	P1	0.02	0.03	0.01	2.36	0.018	0.040	TRUE
ITGB3	P1	0.02	0.02	0.00	4.13	0.000	0.000	TRUE
NTSR1	P1	0.02	0.03	0.01	3.74	0.000	0.001	TRUE
DAGLA	P1	0.02	0.02	0.00	3.38	0.001	0.002	TRUE
TMEM150B	P1	0.02	0.04	0.02	3.65	0.000	0.001	TRUE
TGFA	P1	0.01	0.03	0.00	3.78	0.000	0.000	TRUE
OTOA	P1	0.01	0.03	0.01	3.49	0.000	0.001	TRUE
IL1RL2	P1	0.01	0.02	0.00	5.27	0.000	0.000	TRUE
CX3CR1	P1	0.01	0.02	0.00	2.82	0.005	0.012	TRUE
LILRA6	P1	0.01	0.02	0.00	5.40	0.000	0.000	TRUE
GPR18	P1	0.01	0.03	0.01	2.33	0.020	0.043	TRUE
MMP25	P1	0.01	0.03	0.02	3.37	0.001	0.002	TRUE

SLC4A8	P1	0.01	0.04	0.03	2.96	0.003	0.008	TRUE
LYPD3	P1	0.01	0.02	0.01	2.36	0.018	0.039	TRUE
LILRB5	P1	0.01	0.02	0.00	2.74	0.006	0.015	TRUE
CYSLTR2	P1	0.01	0.06	0.06	2.94	0.003	0.008	TRUE
SLC6A8	P1	0.01	0.03	0.01	3.37	0.001	0.002	TRUE
GABRR2	P1	0.01	0.03	0.00	2.63	0.008	0.019	TRUE
CEACAM3	P1	0.01	0.01	0.00	3.13	0.002	0.004	TRUE
MMP17	P1	0.01	0.02	0.01	2.86	0.004	0.010	TRUE
NCR1	P1	0.01	0.03	0.01	2.44	0.015	0.032	TRUE
VIPR1	P1	0.01	0.02	0.00	3.79	0.000	0.000	TRUE
LRP3	P1	0.01	0.02	0.01	2.29	0.022	0.047	TRUE
GRIN2C	P1	0.01	0.03	0.02	2.53	0.012	0.026	TRUE
NIPAL4	P1	0.01	0.02	0.01	3.53	0.000	0.001	TRUE
SCN4B	P1	0.01	0.01	0.00	3.28	0.001	0.003	TRUE
PCSK5	P1	0.01	0.02	0.00	2.40	0.016	0.036	TRUE
NCR3LG1	P1	0.01	0.03	0.02	2.38	0.017	0.037	TRUE
SORCS2	P1	0.01	0.01	0.00	2.64	0.008	0.019	TRUE
LRRC32	P1	0.01	0.01	0.00	3.07	0.002	0.005	TRUE
LY6K	P1	0.01	0.01	0.00	3.34	0.001	0.002	TRUE
ADAM12	P1	0.01	0.01	0.00	2.78	0.005	0.013	TRUE
ABCA6	P1	0.01	0.01	0.00	2.32	0.020	0.044	TRUE
PKD2L1	P1	0.00	0.01	0.00	2.77	0.006	0.013	TRUE
SLAMF9	P1	0.00	0.01	0.00	3.07	0.002	0.005	TRUE
SLC46A2	P1	0.00	0.00	0.00	2.68	0.007	0.017	TRUE
OR10G3	P1	0.00	0.01	0.00	2.61	0.009	0.021	TRUE
NEO1	P1	0.00	0.00	0.00	2.50	0.012	0.028	TRUE
SLC1A5	P2	0.33	0.71	0.29	30.49	0.000	0.000	TRUE
EBP	P2	0.32	0.84	0.46	24.13	0.000	0.000	TRUE
BSG	P2	0.29	0.96	0.71	19.24	0.000	0.000	TRUE
ATP1B3	P2	0.29	0.85	0.58	19.00	0.000	0.000	TRUE
SLC43A3	P2	0.27	0.60	0.23	28.75	0.000	0.000	TRUE
RPN1	P2	0.27	0.85	0.51	21.37	0.000	0.000	TRUE
SLC3A2	P2	0.25	0.91	0.67	17.82	0.000	0.000	TRUE
NUP210	P2	0.24	0.58	0.16	29.47	0.000	0.000	TRUE
SSR1	P2	0.24	0.85	0.55	17.35	0.000	0.000	TRUE
PRSS21	P2	0.23	0.73	0.38	15.41	0.000	0.000	TRUE
LMAN2	P2	0.22	0.93	0.69	16.00	0.000	0.000	TRUE
SLC29A1	P2	0.21	0.44	0.06	34.55	0.000	0.000	TRUE
CD320	P2	0.20	0.49	0.13	26.95	0.000	0.000	TRUE
SLC39A4	P2	0.20	0.61	0.30	17.69	0.000	0.000	TRUE
EMB	P2	0.19	0.74	0.51	14.00	0.000	0.000	TRUE
TSPAN3	P2	0.18	0.67	0.33	18.80	0.000	0.000	TRUE
TP53I13	P2	0.16	0.69	0.35	16.08	0.000	0.000	TRUE
CD38	P2	0.15	0.67	0.41	13.50	0.000	0.000	TRUE
CD151	P2	0.15	0.60	0.32	15.29	0.000	0.000	TRUE
SLC38A5	P2	0.14	0.43	0.14	22.40	0.000	0.000	TRUE
RNF167	P2	0.13	0.67	0.35	15.84	0.000	0.000	TRUE
SLC7A1	P2	0.13	0.41	0.13	21.81	0.000	0.000	TRUE
DPEP3	P2	0.12	0.64	0.50	5.03	0.000	0.000	TRUE
ITGAE	P2	0.12	0.83	0.59	10.27	0.000	0.000	TRUE
CHPT1	P2	0.12	0.56	0.27	15.73	0.000	0.000	TRUE
SLC2A6	P2	0.11	0.37	0.24	6.69	0.000	0.000	TRUE
TSPAN4	P2	0.11	0.45	0.19	17.31	0.000	0.000	TRUE
SLC19A1	P2	0.11	0.31	0.07	22.41	0.000	0.000	TRUE
SLC16A1	P2	0.11	0.30	0.07	22.73	0.000	0.000	TRUE
SLC52A2	P2	0.11	0.57	0.32	12.34	0.000	0.000	TRUE
SLC1A4	P2	0.10	0.34	0.10	18.19	0.000	0.000	TRUE
PIEZO1	P2	0.10	0.62	0.36	13.51	0.000	0.000	TRUE
SCARB1	P2	0.10	0.35	0.12	18.06	0.000	0.000	TRUE
IL27RA	P2	0.10	0.46	0.24	12.91	0.000	0.000	TRUE
NETO2	P2	0.10	0.26	0.07	18.57	0.000	0.000	TRUE
CSF2RB	P2	0.10	0.32	0.19	7.17	0.000	0.000	TRUE
SLC5A6	P2	0.10	0.30	0.09	18.27	0.000	0.000	TRUE
LRP8	P2	0.09	0.35	0.13	18.27	0.000	0.000	TRUE
ADAM15	P2	0.09	0.39	0.18	15.20	0.000	0.000	TRUE
ADCY3	P2	0.09	0.40	0.17	16.67	0.000	0.000	TRUE
TMEM245	P2	0.09	0.39	0.17	14.41	0.000	0.000	TRUE
INSR	P2	0.08	0.34	0.11	16.61	0.000	0.000	TRUE
SLC37A4	P2	0.08	0.26	0.06	19.96	0.000	0.000	TRUE
SLC39A6	P2	0.08	0.38	0.17	14.40	0.000	0.000	TRUE
SPPL2B	P2	0.08	0.47	0.25	12.39	0.000	0.000	TRUE
TMEM161A	P2	0.07	0.33	0.12	15.40	0.000	0.000	TRUE
SLC43A1	P2	0.07	0.28	0.09	17.73	0.000	0.000	TRUE
TRGC2	P2	0.07	0.33	0.16	11.31	0.000	0.000	TRUE
SLC39A14	P2	0.07	0.20	0.04	17.33	0.000	0.000	TRUE
PKD1	P2	0.07	0.37	0.16	15.22	0.000	0.000	TRUE
SLC22A16	P2	0.07	0.31	0.13	13.15	0.000	0.000	TRUE
MICB	P2	0.07	0.26	0.08	16.59	0.000	0.000	TRUE
HYAL2	P2	0.07	0.28	0.12	11.11	0.000	0.000	TRUE
EPCAM	P2	0.06	0.16	0.05	11.33	0.000	0.000	TRUE
CD59	P2	0.06	0.50	0.34	7.42	0.000	0.000	TRUE
SCAP	P2	0.06	0.34	0.14	14.03	0.000	0.000	TRUE
ATP13A1	P2	0.06	0.34	0.15	13.47	0.000	0.000	TRUE
GPR180	P2	0.06	0.26	0.09	13.97	0.000	0.000	TRUE
CNNM3	P2	0.06	0.26	0.09	15.15	0.000	0.000	TRUE
LAMP5	P2	0.06	0.09	0.02	5.51	0.000	0.000	TRUE
TMX3	P2	0.06	0.46	0.25	11.20	0.000	0.000	TRUE
PTK7	P2	0.06	0.27	0.10	16.44	0.000	0.000	TRUE
CLSTN1	P2	0.06	0.49	0.28	9.51	0.000	0.000	TRUE
LTB4R	P2	0.06	0.28	0.10	15.88	0.000	0.000	TRUE
SLC4A7	P2	0.06	0.31	0.13	10.80	0.000	0.000	TRUE
C11orf24	P2	0.06	0.22	0.07	13.69	0.000	0.000	TRUE
TM9SF1	P2	0.06	0.38	0.20	11.39	0.000	0.000	TRUE
GINM1	P2	0.06	0.64	0.41	7.68	0.000	0.000	TRUE
SLC7A6	P2	0.05	0.34	0.17	9.37	0.000	0.000	TRUE
ADIPOR2	P2	0.05	0.49	0.28	10.76	0.000	0.000	TRUE
SLC12A2	P2	0.05	0.29	0.14	10.11	0.000	0.000	TRUE
FLVCR1	P2	0.05	0.26	0.12	11.70	0.000	0.000	TRUE
VSIG10	P2	0.05	0.24	0.09	14.16	0.000	0.000	TRUE
KCNK5	P2	0.05	0.21	0.09	11.71	0.000	0.000	TRUE
SLC16A7	P2	0.04	0.35	0.18	13.08	0.000	0.000	TRUE

TSPAN13	P2	0.04	0.36	0.22	7.68	0.000	0.000	TRUE
LRP5	P2	0.04	0.22	0.08	11.68	0.000	0.000	TRUE
FAM189B	P2	0.04	0.21	0.08	11.74	0.000	0.000	TRUE
NRXN2	P2	0.04	0.20	0.09	11.40	0.000	0.000	TRUE
TCTN3	P2	0.04	0.38	0.21	9.70	0.000	0.000	TRUE
GPR27	P2	0.04	0.31	0.17	9.29	0.000	0.000	TRUE
TMEM9	P2	0.04	0.28	0.14	12.27	0.000	0.000	TRUE
LMBR1	P2	0.04	0.26	0.12	12.08	0.000	0.000	TRUE
SLC20A2	P2	0.04	0.32	0.15	9.74	0.000	0.000	TRUE
TMEM67	P2	0.04	0.21	0.09	10.69	0.000	0.000	TRUE
TSPAN5	P2	0.04	0.17	0.06	9.17	0.000	0.000	TRUE
FGFRL1	P2	0.04	0.19	0.08	10.32	0.000	0.000	TRUE
SLC41A3	P2	0.04	0.30	0.16	11.35	0.000	0.000	TRUE
SIGLEC12	P2	0.04	0.10	0.02	11.53	0.000	0.000	TRUE
ZDHHC5	P2	0.04	0.42	0.24	9.52	0.000	0.000	TRUE
ABCA2	P2	0.04	0.21	0.09	12.34	0.000	0.000	TRUE
TPRA1	P2	0.03	0.24	0.12	9.82	0.000	0.000	TRUE
DAG1	P2	0.03	0.14	0.05	9.82	0.000	0.000	TRUE
BACE1	P2	0.03	0.18	0.08	10.65	0.000	0.000	TRUE
QSOX2	P2	0.03	0.21	0.10	10.76	0.000	0.000	TRUE
CHRNA5	P2	0.03	0.09	0.01	11.71	0.000	0.000	TRUE
PILRB	P2	0.03	0.36	0.21	7.62	0.000	0.000	TRUE
MFSD5	P2	0.03	0.21	0.10	7.98	0.000	0.000	TRUE
DAGLB	P2	0.03	0.28	0.14	10.02	0.000	0.000	TRUE
MRC2	P2	0.03	0.21	0.11	10.15	0.000	0.000	TRUE
C1orf159	P2	0.03	0.14	0.05	10.41	0.000	0.000	TRUE
SUSD3	P2	0.03	0.17	0.09	8.89	0.000	0.000	TRUE
GPC6	P2	0.03	0.12	0.04	4.64	0.000	0.000	TRUE
SLC12A5	P2	0.03	0.15	0.06	9.38	0.000	0.000	TRUE
RTN4R	P2	0.03	0.10	0.03	11.74	0.000	0.000	TRUE
SLC2A8	P2	0.03	0.10	0.03	9.32	0.000	0.000	TRUE
FAM171A2	P2	0.02	0.11	0.03	10.42	0.000	0.000	TRUE
CDH4	P2	0.02	0.15	0.06	4.87	0.000	0.000	TRUE
ESAM	P2	0.02	0.14	0.07	6.47	0.000	0.000	TRUE
KLRG1	P2	0.02	0.14	0.06	9.51	0.000	0.000	TRUE
P2RY1	P2	0.02	0.14	0.05	9.53	0.000	0.000	TRUE
SLC2A5	P2	0.02	0.13	0.06	7.29	0.000	0.000	TRUE
IGSF8	P2	0.02	0.24	0.13	8.25	0.000	0.000	TRUE
ADORA2B	P2	0.02	0.09	0.03	8.39	0.000	0.000	TRUE
CEACAM6	P2	0.02	0.08	0.03	3.52	0.000	0.001	TRUE
TSPAN7	P2	0.02	0.13	0.06	6.08	0.000	0.000	TRUE
TMEM62	P2	0.02	0.16	0.07	7.36	0.000	0.000	TRUE
TPSG1	P2	0.02	0.04	0.01	2.47	0.013	0.030	TRUE
GPR137	P2	0.02	0.28	0.16	9.68	0.000	0.000	TRUE
TSPAN11	P2	0.02	0.04	0.00	4.66	0.000	0.000	TRUE
LMAN2L	P2	0.02	0.12	0.05	9.37	0.000	0.000	TRUE
IGF1R	P2	0.02	0.14	0.07	8.71	0.000	0.000	TRUE
PODXL2	P2	0.02	0.10	0.04	9.14	0.000	0.000	TRUE
CELSR3	P2	0.02	0.06	0.01	8.87	0.000	0.000	TRUE
LRP11	P2	0.02	0.05	0.01	5.89	0.000	0.000	TRUE
LRRN1	P2	0.02	0.09	0.03	4.60	0.000	0.000	TRUE
P2RY11	P2	0.02	0.15	0.08	8.24	0.000	0.000	TRUE
NCR3	P2	0.02	0.07	0.03	5.88	0.000	0.000	TRUE
DGCR2	P2	0.02	0.14	0.06	8.93	0.000	0.000	TRUE
LRP4	P2	0.02	0.05	0.01	5.51	0.000	0.000	TRUE
SLC26A6	P2	0.02	0.11	0.05	7.64	0.000	0.000	TRUE
PIGO	P2	0.02	0.14	0.07	8.06	0.000	0.000	TRUE
TMEM116	P2	0.02	0.10	0.04	8.79	0.000	0.000	TRUE
JAM3	P2	0.02	0.08	0.02	6.55	0.000	0.000	TRUE
ADAM22	P2	0.02	0.07	0.02	6.12	0.000	0.000	TRUE
DCBLD2	P2	0.02	0.07	0.02	6.75	0.000	0.000	TRUE
SLC29A2	P2	0.02	0.06	0.02	8.03	0.000	0.000	TRUE
TYRO3	P2	0.01	0.05	0.01	8.43	0.000	0.000	TRUE
CDH24	P2	0.01	0.05	0.01	8.19	0.000	0.000	TRUE
SLC2A13	P2	0.01	0.06	0.02	6.65	0.000	0.000	TRUE
SLC38A9	P2	0.01	0.17	0.09	7.70	0.000	0.000	TRUE
ACVR2B	P2	0.01	0.05	0.01	6.85	0.000	0.000	TRUE
ENPP6	P2	0.01	0.02	0.00	3.23	0.001	0.003	TRUE
ABCG2	P2	0.01	0.04	0.01	5.56	0.000	0.000	TRUE
CD19	P2	0.01	0.05	0.02	6.00	0.000	0.000	TRUE
FGFR1	P2	0.01	0.12	0.07	5.96	0.000	0.000	TRUE
GPC1	P2	0.01	0.04	0.01	7.09	0.000	0.000	TRUE
VNN1	P2	0.01	0.09	0.05	3.82	0.000	0.000	TRUE
ITGA7	P2	0.01	0.03	0.01	4.79	0.000	0.000	TRUE
RAMP2	P2	0.01	0.04	0.01	5.73	0.000	0.000	TRUE
RNF150	P2	0.01	0.04	0.01	3.02	0.003	0.006	TRUE
TNFRSF8	P2	0.01	0.05	0.01	5.02	0.000	0.000	TRUE
TCTN2	P2	0.01	0.06	0.02	6.95	0.000	0.000	TRUE
GPR3	P2	0.01	0.04	0.01	7.17	0.000	0.000	TRUE
CD163L1	P2	0.01	0.03	0.01	3.05	0.002	0.006	TRUE
EPHB4	P2	0.01	0.05	0.02	5.60	0.000	0.000	TRUE
NRG2	P2	0.01	0.04	0.01	5.29	0.000	0.000	TRUE
ABCB9	P2	0.01	0.06	0.02	6.70	0.000	0.000	TRUE
CLDN7	P2	0.01	0.09	0.04	4.61	0.000	0.000	TRUE
GJA3	P2	0.01	0.04	0.01	6.57	0.000	0.000	TRUE
ATP1A3	P2	0.01	0.04	0.01	6.65	0.000	0.000	TRUE
SMO	P2	0.01	0.03	0.01	6.19	0.000	0.000	TRUE
SLC46A1	P2	0.01	0.03	0.01	7.09	0.000	0.000	TRUE
LHFPL5	P2	0.01	0.06	0.03	4.45	0.000	0.000	TRUE
SLC22A5	P2	0.01	0.04	0.02	6.03	0.000	0.000	TRUE
SLC2A10	P2	0.01	0.03	0.01	3.52	0.000	0.001	TRUE
SLC16A4	P2	0.01	0.07	0.03	5.14	0.000	0.000	TRUE
MCAM	P2	0.01	0.03	0.01	3.57	0.000	0.001	TRUE
ASIC1	P2	0.01	0.02	0.00	5.97	0.000	0.000	TRUE
NLGN2	P2	0.01	0.04	0.01	6.06	0.000	0.000	TRUE
EVA1C	P2	0.01	0.04	0.01	3.75	0.000	0.001	TRUE
LYPD5	P2	0.01	0.02	0.00	4.80	0.000	0.000	TRUE
DLL3	P2	0.01	0.02	0.00	6.03	0.000	0.000	TRUE
SLC15A2	P2	0.01	0.07	0.04	6.20	0.000	0.000	TRUE
SCNN1D	P2	0.01	0.03	0.01	4.07	0.000	0.000	TRUE
SLC19A2	P2	0.01	0.08	0.04	5.56	0.000	0.000	TRUE

GPR137C	P2	0.01	0.03	0.01	7.27	0.000	0.000	TRUE
MAMDC4	P2	0.01	0.04	0.01	4.78	0.000	0.000	TRUE
OXER1	P2	0.01	0.04	0.01	4.84	0.000	0.000	TRUE
KISS1R	P2	0.01	0.02	0.00	3.81	0.000	0.000	TRUE
PIEZO2	P2	0.01	0.02	0.00	5.66	0.000	0.000	TRUE
LPAR4	P2	0.01	0.06	0.03	3.50	0.000	0.001	TRUE
FOLH1	P2	0.01	0.04	0.01	2.76	0.006	0.014	TRUE
EFNA2	P2	0.01	0.04	0.02	2.48	0.013	0.029	TRUE
KDR	P2	0.01	0.01	0.00	2.47	0.014	0.030	TRUE
SLC29A4	P2	0.01	0.02	0.00	4.58	0.000	0.000	TRUE
NPHS1	P2	0.01	0.02	0.00	3.98	0.000	0.000	TRUE
SUSD5	P2	0.00	0.02	0.00	2.88	0.004	0.010	TRUE
EFNB2	P2	0.00	0.05	0.02	3.64	0.000	0.001	TRUE
EPOR	P2	0.00	0.03	0.01	5.18	0.000	0.000	TRUE
SLC2A4	P2	0.00	0.01	0.00	3.77	0.000	0.000	TRUE
CSPG5	P2	0.00	0.02	0.00	4.91	0.000	0.000	TRUE
CUZD1	P2	0.00	0.02	0.01	2.52	0.012	0.026	TRUE
GPR150	P2	0.00	0.01	0.00	2.87	0.004	0.010	TRUE
FZD7	P2	0.00	0.04	0.02	3.99	0.000	0.000	TRUE
TSPAN6	P2	0.00	0.01	0.00	3.02	0.003	0.006	TRUE
CHRNA10	P2	0.00	0.04	0.02	2.62	0.009	0.020	TRUE
SEMA4F	P2	0.00	0.02	0.01	3.98	0.000	0.000	TRUE
SLC6A9	P2	0.00	0.02	0.01	3.81	0.000	0.000	TRUE
GPR19	P2	0.00	0.02	0.00	5.52	0.000	0.000	TRUE
KIR3DL2	P2	0.00	0.01	0.00	4.39	0.000	0.000	TRUE
SCN8A	P2	0.00	0.01	0.00	3.85	0.000	0.000	TRUE
ITGB4	P2	0.00	0.02	0.01	3.60	0.000	0.001	TRUE
MAG	P2	0.00	0.01	0.00	3.35	0.001	0.002	TRUE
LRRC3B	P2	0.00	0.02	0.00	2.84	0.005	0.011	TRUE
ADRA2B	P2	0.00	0.05	0.03	3.05	0.002	0.006	TRUE
CD8B	P2	0.00	0.01	0.00	3.04	0.002	0.006	TRUE
VSIG8	P2	0.00	0.02	0.01	2.49	0.013	0.028	TRUE
IL12RB2	P2	0.00	0.03	0.01	3.45	0.001	0.001	TRUE
IL2RB	P2	0.00	0.05	0.02	3.05	0.002	0.006	TRUE
ALK	P2	0.00	0.01	0.00	2.28	0.023	0.049	TRUE
TNFRSF10C	P2	0.00	0.02	0.01	4.50	0.000	0.000	TRUE
EFNA3	P2	0.00	0.03	0.02	3.22	0.001	0.003	TRUE
SELP	P2	0.00	0.01	0.00	2.72	0.007	0.016	TRUE
CACNG4	P2	0.00	0.02	0.01	2.50	0.012	0.028	TRUE
SLC9A3	P2	0.00	0.01	0.00	3.33	0.001	0.002	TRUE
SLC18A2	P2	0.00	0.02	0.01	2.32	0.021	0.044	TRUE
OR10A2	P2	0.00	0.01	0.00	2.52	0.012	0.027	TRUE
LRFN1	P2	0.00	0.03	0.02	3.33	0.001	0.002	TRUE
RXFP2	P2	0.00	0.01	0.00	2.78	0.005	0.013	TRUE
TAS2R20	P2	0.00	0.01	0.00	2.30	0.022	0.046	TRUE
EPHA1	P2	0.00	0.01	0.00	2.62	0.009	0.020	TRUE
OR4F15	P2	0.00	0.01	0.00	2.57	0.010	0.023	TRUE
APLP1	P2	0.00	0.01	0.01	3.01	0.003	0.006	TRUE
OR6A2	P2	0.00	0.01	0.00	2.60	0.009	0.021	TRUE
AMIGO1	P2	0.00	0.01	0.00	2.28	0.023	0.048	TRUE
SLC26A4	P2	0.00	0.01	0.00	2.39	0.017	0.037	TRUE
OR8D4	P2	0.00	0.01	0.00	2.40	0.016	0.036	TRUE
BEST2	P2	0.00	0.01	0.00	3.32	0.001	0.002	TRUE
ASTN2	P2	0.00	0.01	0.00	2.88	0.004	0.010	TRUE
GFRA3	P2	0.00	0.01	0.00	2.56	0.010	0.023	TRUE
FLRT1	P2	0.00	0.00	0.00	2.63	0.009	0.020	TRUE
ABCA12	P2	0.00	0.00	0.00	2.28	0.022	0.048	TRUE
SLC4A4	P2	0.00	0.00	0.00	2.30	0.021	0.046	TRUE
OR2AG1	P2	0.00	0.00	0.00	2.37	0.018	0.038	TRUE
VSIG10L	P2	0.00	0.01	0.01	2.49	0.013	0.029	TRUE
SLC5A2	P2	0.00	0.00	0.00	2.26	0.024	0.050	TRUE
CD52	P3	0.45	0.83	0.62	24.32	0.000	0.000	TRUE
SPN	P3	0.39	0.90	0.63	26.38	0.000	0.000	TRUE
CD69	P3	0.38	0.80	0.60	18.50	0.000	0.000	TRUE
GYPC	P3	0.31	0.82	0.68	17.65	0.000	0.000	TRUE
LY6E	P3	0.31	0.83	0.62	18.51	0.000	0.000	TRUE
CD63	P3	0.30	0.98	0.90	16.78	0.000	0.000	TRUE
CD34	P3	0.29	0.60	0.38	22.64	0.000	0.000	TRUE
RNF130	P3	0.29	0.89	0.67	18.56	0.000	0.000	TRUE
CD96	P3	0.28	0.68	0.42	22.87	0.000	0.000	TRUE
ITGA4	P3	0.27	0.82	0.61	16.57	0.000	0.000	TRUE
BST2	P3	0.24	0.90	0.77	13.26	0.000	0.000	TRUE
HLA-DRB1	P3	0.23	0.90	0.81	10.82	0.000	0.000	TRUE
TM7SF3	P3	0.23	0.67	0.40	21.86	0.000	0.000	TRUE
HLA-DMA	P3	0.22	0.88	0.70	11.78	0.000	0.000	TRUE
ITM2B	P3	0.22	0.99	0.91	11.01	0.000	0.000	TRUE
ATP6V0A2	P3	0.21	0.54	0.37	16.56	0.000	0.000	TRUE
CD47	P3	0.21	0.92	0.76	12.94	0.000	0.000	TRUE
IL2RG	P3	0.20	0.85	0.66	14.38	0.000	0.000	TRUE
CD82	P3	0.19	0.82	0.65	13.03	0.000	0.000	TRUE
TMEM219	P3	0.19	0.85	0.65	13.79	0.000	0.000	TRUE
HLA-A	P3	0.19	1.00	0.98	12.27	0.000	0.000	TRUE
SYPL1	P3	0.18	0.70	0.49	16.16	0.000	0.000	TRUE
CD37	P3	0.18	0.84	0.66	14.71	0.000	0.000	TRUE
ICAM3	P3	0.17	0.66	0.49	17.19	0.000	0.000	TRUE
SPINT2	P3	0.17	0.74	0.58	12.59	0.000	0.000	TRUE
M6PR	P3	0.16	0.73	0.55	15.05	0.000	0.000	TRUE
SELL	P3	0.16	0.45	0.31	13.88	0.000	0.000	TRUE
ATRAID	P3	0.16	0.69	0.48	15.46	0.000	0.000	TRUE
ITM2C	P3	0.16	0.63	0.46	12.71	0.000	0.000	TRUE
F2R	P3	0.16	0.29	0.12	18.78	0.000	0.000	TRUE
IL1RL1	P3	0.15	0.15	0.05	12.20	0.000	0.000	TRUE
STT3B	P3	0.15	0.80	0.66	12.25	0.000	0.000	TRUE
HLA-E	P3	0.15	0.99	0.93	8.29	0.000	0.000	TRUE
S1PR4	P3	0.14	0.55	0.36	16.82	0.000	0.000	TRUE
TMEM179B	P3	0.14	0.59	0.41	13.75	0.000	0.000	TRUE
PTTG1IP	P3	0.14	0.73	0.56	11.33	0.000	0.000	TRUE
HLA-C	P3	0.14	1.00	0.96	5.81	0.000	0.000	TRUE
ICAM2	P3	0.14	0.53	0.35	15.00	0.000	0.000	TRUE
CD9	P3	0.14	0.32	0.17	15.27	0.000	0.000	TRUE
KIT	P3	0.13	0.33	0.18	15.17	0.000	0.000	TRUE

SORL1	P3	0.12	0.46	0.31	13.87	0.000	0.000	TRUE
SERINC3	P3	0.12	0.61	0.43	13.39	0.000	0.000	TRUE
ABCC4	P3	0.12	0.24	0.11	14.47	0.000	0.000	TRUE
HLA-DRB5	P3	0.12	0.68	0.59	8.62	0.000	0.000	TRUE
SLC9A7	P3	0.12	0.45	0.29	13.62	0.000	0.000	TRUE
PROM1	P3	0.12	0.36	0.19	16.37	0.000	0.000	TRUE
TGOLN2	P3	0.12	0.81	0.66	8.29	0.000	0.000	TRUE
NPR3	P3	0.11	0.26	0.13	14.61	0.000	0.000	TRUE
SLC39A8	P3	0.11	0.45	0.30	12.64	0.000	0.000	TRUE
TNFRSF14	P3	0.11	0.63	0.46	11.61	0.000	0.000	TRUE
ABCC1	P3	0.11	0.51	0.34	12.42	0.000	0.000	TRUE
CD164	P3	0.11	0.91	0.78	6.78	0.000	0.000	TRUE
CD84	P3	0.11	0.36	0.22	13.01	0.000	0.000	TRUE
CD79B	P3	0.11	0.31	0.16	18.13	0.000	0.000	TRUE
TFPI	P3	0.11	0.46	0.32	9.97	0.000	0.000	TRUE
NTRK1	P3	0.10	0.15	0.03	15.53	0.000	0.000	TRUE
CD33	P3	0.10	0.40	0.27	11.14	0.000	0.000	TRUE
IL1RAP	P3	0.10	0.44	0.31	11.74	0.000	0.000	TRUE
IL9R	P3	0.10	0.23	0.10	14.38	0.000	0.000	TRUE
ITGA2B	P3	0.09	0.15	0.05	13.35	0.000	0.000	TRUE
PTPRA	P3	0.09	0.65	0.50	8.80	0.000	0.000	TRUE
MFSD12	P3	0.09	0.44	0.31	10.07	0.000	0.000	TRUE
F11R	P3	0.09	0.50	0.35	11.81	0.000	0.000	TRUE
LAMP2	P3	0.09	0.76	0.62	7.40	0.000	0.000	TRUE
BTN3A2	P3	0.09	0.37	0.24	12.37	0.000	0.000	TRUE
PKD2	P3	0.09	0.32	0.20	11.21	0.000	0.000	TRUE
SPNS3	P3	0.09	0.31	0.24	6.55	0.000	0.000	TRUE
APP	P3	0.08	0.48	0.35	8.83	0.000	0.000	TRUE
PIGT	P3	0.08	0.43	0.30	11.14	0.000	0.000	TRUE
TGFBR2	P3	0.08	0.39	0.26	11.59	0.000	0.000	TRUE
CD244	P3	0.08	0.28	0.16	14.64	0.000	0.000	TRUE
SIGIRR	P3	0.08	0.43	0.30	11.15	0.000	0.000	TRUE
P2RX1	P3	0.08	0.36	0.22	13.99	0.000	0.000	TRUE
ATP1B1	P3	0.08	0.31	0.20	12.12	0.000	0.000	TRUE
P2RY8	P3	0.08	0.34	0.23	9.93	0.000	0.000	TRUE
SEMA7A	P3	0.08	0.24	0.14	10.31	0.000	0.000	TRUE
CD46	P3	0.08	0.74	0.60	6.83	0.000	0.000	TRUE
SLC40A1	P3	0.07	0.14	0.07	7.93	0.000	0.000	TRUE
SLC39A10	P3	0.07	0.43	0.31	8.59	0.000	0.000	TRUE
CD300A	P3	0.07	0.24	0.14	11.67	0.000	0.000	TRUE
SLC12A6	P3	0.07	0.47	0.36	7.92	0.000	0.000	TRUE
TRPV2	P3	0.07	0.26	0.15	12.64	0.000	0.000	TRUE
SV2A	P3	0.07	0.25	0.15	10.92	0.000	0.000	TRUE
FAM171A1	P3	0.07	0.22	0.11	9.67	0.000	0.000	TRUE
SLC17A9	P3	0.06	0.25	0.16	11.93	0.000	0.000	TRUE
GLIPR1	P3	0.06	0.49	0.44	4.14	0.000	0.000	TRUE
RNFT1	P3	0.06	0.28	0.16	10.49	0.000	0.000	TRUE
TMCO3	P3	0.06	0.52	0.41	8.29	0.000	0.000	TRUE
TM4SF1	P3	0.06	0.22	0.16	7.16	0.000	0.000	TRUE
TMIGD2	P3	0.06	0.21	0.12	9.96	0.000	0.000	TRUE
GPR174	P3	0.06	0.34	0.22	11.54	0.000	0.000	TRUE
TNFRSF1A	P3	0.06	0.43	0.33	7.17	0.000	0.000	TRUE
CLN3	P3	0.05	0.35	0.24	8.69	0.000	0.000	TRUE
ITFG1	P3	0.05	0.32	0.22	9.70	0.000	0.000	TRUE
ATP2B4	P3	0.05	0.30	0.21	6.66	0.000	0.000	TRUE
SEMA4D	P3	0.05	0.34	0.24	8.05	0.000	0.000	TRUE
AMN	P3	0.05	0.21	0.13	9.12	0.000	0.000	TRUE
EPHB6	P3	0.05	0.21	0.12	10.81	0.000	0.000	TRUE
SUSD1	P3	0.05	0.29	0.20	9.55	0.000	0.000	TRUE
NRROS	P3	0.05	0.35	0.24	6.68	0.000	0.000	TRUE
TMEM106B	P3	0.05	0.21	0.12	10.02	0.000	0.000	TRUE
TSPAN31	P3	0.05	0.29	0.19	9.42	0.000	0.000	TRUE
GP1BB	P3	0.05	0.12	0.05	8.97	0.000	0.000	TRUE
ITGA9	P3	0.05	0.17	0.09	11.10	0.000	0.000	TRUE
NCSTN	P3	0.05	0.29	0.20	7.71	0.000	0.000	TRUE
CDCP1	P3	0.05	0.17	0.12	6.51	0.000	0.000	TRUE
STIM1	P3	0.05	0.31	0.21	9.27	0.000	0.000	TRUE
SLC37A1	P3	0.05	0.34	0.25	6.20	0.000	0.000	TRUE
PLXNA1	P3	0.05	0.12	0.07	5.55	0.000	0.000	TRUE
TMEM63A	P3	0.04	0.22	0.13	8.36	0.000	0.000	TRUE
RXFP1	P3	0.04	0.13	0.07	5.58	0.000	0.000	TRUE
LTBR	P3	0.04	0.33	0.24	7.58	0.000	0.000	TRUE
CTNS	P3	0.04	0.18	0.11	7.22	0.000	0.000	TRUE
NOTCH1	P3	0.04	0.29	0.21	8.89	0.000	0.000	TRUE
ICAM4	P3	0.04	0.20	0.13	8.21	0.000	0.000	TRUE
KCNMB3	P3	0.04	0.13	0.07	8.55	0.000	0.000	TRUE
UMODL1	P3	0.04	0.16	0.11	6.28	0.000	0.000	TRUE
LRRC37B	P3	0.04	0.23	0.16	5.56	0.000	0.000	TRUE
LRP6	P3	0.04	0.17	0.09	3.98	0.000	0.000	TRUE
ERMP1	P3	0.04	0.15	0.09	7.26	0.000	0.000	TRUE
SIGLEC6	P3	0.04	0.08	0.03	6.64	0.000	0.000	TRUE
TMEM150A	P3	0.04	0.14	0.07	8.87	0.000	0.000	TRUE
TM9SF4	P3	0.04	0.24	0.16	9.27	0.000	0.000	TRUE
TXNDC15	P3	0.04	0.22	0.15	10.13	0.000	0.000	TRUE
SLC24A3	P3	0.04	0.09	0.02	6.78	0.000	0.000	TRUE
GPR12	P3	0.04	0.09	0.04	3.29	0.001	0.003	TRUE
C3orf80	P3	0.04	0.15	0.10	7.42	0.000	0.000	TRUE
TNFRSF18	P3	0.04	0.23	0.18	6.99	0.000	0.000	TRUE
ATP13A2	P3	0.04	0.15	0.09	8.53	0.000	0.000	TRUE
ADCY6	P3	0.03	0.13	0.07	9.29	0.000	0.000	TRUE
THSD7A	P3	0.03	0.08	0.02	3.78	0.000	0.000	TRUE
RYK	P3	0.03	0.24	0.17	7.32	0.000	0.000	TRUE
GRIK5	P3	0.03	0.12	0.06	8.83	0.000	0.000	TRUE
CD7	P3	0.03	0.13	0.08	5.56	0.000	0.000	TRUE
HTR1F	P3	0.03	0.13	0.07	9.76	0.000	0.000	TRUE
GJA1	P3	0.03	0.09	0.05	7.51	0.000	0.000	TRUE
SUCNR1	P3	0.03	0.18	0.13	5.29	0.000	0.000	TRUE
CASD1	P3	0.03	0.16	0.10	7.97	0.000	0.000	TRUE
MFSD6	P3	0.03	0.20	0.13	8.60	0.000	0.000	TRUE
SVOPL	P3	0.03	0.12	0.07	7.86	0.000	0.000	TRUE
CLEC14A	P3	0.03	0.15	0.10	5.62	0.000	0.000	TRUE

SLC33A1	P3	0.03	0.23	0.16	7.58	0.000	0.000	TRUE
CHRNB1	P3	0.03	0.23	0.17	7.38	0.000	0.000	TRUE
NIPAL2	P3	0.03	0.11	0.06	8.49	0.000	0.000	TRUE
ACE	P3	0.03	0.09	0.04	7.48	0.000	0.000	TRUE
GP1BA	P3	0.03	0.08	0.04	5.23	0.000	0.000	TRUE
LPAR5	P3	0.03	0.07	0.03	8.35	0.000	0.000	TRUE
SPNS2	P3	0.03	0.16	0.11	5.57	0.000	0.000	TRUE
ROBO3	P3	0.03	0.18	0.13	5.61	0.000	0.000	TRUE
BACE2	P3	0.03	0.11	0.07	3.90	0.000	0.000	TRUE
SLC46A3	P3	0.03	0.11	0.06	4.95	0.000	0.000	TRUE
SLC26A2	P3	0.03	0.17	0.12	6.36	0.000	0.000	TRUE
SLC39A9	P3	0.03	0.18	0.12	6.62	0.000	0.000	TRUE
LRFN4	P3	0.03	0.14	0.09	7.04	0.000	0.000	TRUE
CD79A	P3	0.03	0.12	0.08	5.78	0.000	0.000	TRUE
FAM174A	P3	0.03	0.12	0.07	8.63	0.000	0.000	TRUE
CLDN15	P3	0.02	0.18	0.12	6.83	0.000	0.000	TRUE
BTN3A3	P3	0.02	0.14	0.09	7.73	0.000	0.000	TRUE
SLC44A2	P3	0.02	0.13	0.08	5.18	0.000	0.000	TRUE
PSEN2	P3	0.02	0.09	0.05	4.23	0.000	0.000	TRUE
SEMA6A	P3	0.02	0.03	0.01	4.06	0.000	0.000	TRUE
ZFYVE27	P3	0.02	0.18	0.13	5.34	0.000	0.000	TRUE
TIE1	P3	0.02	0.07	0.03	8.34	0.000	0.000	TRUE
NAGPA	P3	0.02	0.15	0.11	6.38	0.000	0.000	TRUE
BTN3A1	P3	0.02	0.15	0.10	6.25	0.000	0.000	TRUE
MFSD8	P3	0.02	0.15	0.10	6.82	0.000	0.000	TRUE
F2RL3	P3	0.02	0.05	0.01	7.62	0.000	0.000	TRUE
HLA-DQA2	P3	0.02	0.10	0.07	3.66	0.000	0.001	TRUE
NTNG2	P3	0.02	0.11	0.07	6.41	0.000	0.000	TRUE
ACVR1	P3	0.02	0.11	0.07	7.35	0.000	0.000	TRUE
TSPAN2	P3	0.02	0.08	0.05	5.28	0.000	0.000	TRUE
ABCC5	P3	0.02	0.12	0.08	5.82	0.000	0.000	TRUE
TRAT1	P3	0.02	0.08	0.03	5.23	0.000	0.000	TRUE
CDH26	P3	0.02	0.08	0.05	3.83	0.000	0.000	TRUE
TNFRSF25	P3	0.02	0.13	0.10	5.31	0.000	0.000	TRUE
FLT3LG	P3	0.02	0.08	0.04	6.35	0.000	0.000	TRUE
TMEM25	P3	0.02	0.07	0.04	5.42	0.000	0.000	TRUE
IL18R1	P3	0.02	0.17	0.12	2.58	0.010	0.022	TRUE
S1PR1	P3	0.02	0.06	0.03	3.37	0.001	0.002	TRUE
DPEP2	P3	0.02	0.10	0.06	7.83	0.000	0.000	TRUE
IL12RB1	P3	0.02	0.11	0.07	4.44	0.000	0.000	TRUE
IL17RE	P3	0.02	0.08	0.05	7.50	0.000	0.000	TRUE
CDH9	P3	0.02	0.04	0.02	2.33	0.020	0.042	TRUE
SLC12A4	P3	0.02	0.10	0.07	4.28	0.000	0.000	TRUE
SLC2A11	P3	0.02	0.09	0.06	6.52	0.000	0.000	TRUE
TBXA2R	P3	0.02	0.04	0.01	6.80	0.000	0.000	TRUE
NAALADL1	P3	0.02	0.06	0.03	4.36	0.000	0.000	TRUE
ACP2	P3	0.02	0.09	0.06	4.02	0.000	0.000	TRUE
ATRN	P3	0.02	0.13	0.09	4.36	0.000	0.000	TRUE
LMBRD2	P3	0.02	0.09	0.06	5.90	0.000	0.000	TRUE
MEGF8	P3	0.01	0.08	0.05	6.62	0.000	0.000	TRUE
TSPAN17	P3	0.01	0.11	0.08	5.14	0.000	0.000	TRUE
MICA	P3	0.01	0.12	0.09	4.75	0.000	0.000	TRUE
NRG4	P3	0.01	0.08	0.06	3.01	0.003	0.006	TRUE
PLXNA3	P3	0.01	0.09	0.06	5.79	0.000	0.000	TRUE
GPR146	P3	0.01	0.08	0.05	6.13	0.000	0.000	TRUE
ZP3	P3	0.01	0.07	0.04	4.86	0.000	0.000	TRUE
ICAM5	P3	0.01	0.05	0.02	5.61	0.000	0.000	TRUE
SLC16A5	P3	0.01	0.06	0.03	5.97	0.000	0.000	TRUE
TMEM182	P3	0.01	0.05	0.03	6.80	0.000	0.000	TRUE
KIRREL3	P3	0.01	0.03	0.00	6.09	0.000	0.000	TRUE
SLC35A5	P3	0.01	0.09	0.06	5.60	0.000	0.000	TRUE
SLC29A3	P3	0.01	0.08	0.05	4.13	0.000	0.000	TRUE
TMEM204	P3	0.01	0.05	0.03	4.28	0.000	0.000	TRUE
MEGF10	P3	0.01	0.04	0.02	2.76	0.006	0.014	TRUE
IL17RC	P3	0.01	0.09	0.06	6.18	0.000	0.000	TRUE
SLC45A4	P3	0.01	0.08	0.05	4.84	0.000	0.000	TRUE
TMEM140	P3	0.01	0.14	0.10	3.43	0.001	0.002	TRUE
LTK	P3	0.01	0.04	0.02	5.75	0.000	0.000	TRUE
LCT	P3	0.01	0.04	0.03	3.17	0.002	0.004	TRUE
AQP2	P3	0.01	0.01	0.00	3.18	0.001	0.004	TRUE
HLA-G	P3	0.01	0.08	0.06	2.43	0.015	0.033	TRUE
DPEP1	P3	0.01	0.07	0.05	3.50	0.000	0.001	TRUE
SLAMF6	P3	0.01	0.06	0.03	4.43	0.000	0.000	TRUE
ABCC10	P3	0.01	0.06	0.04	3.87	0.000	0.000	TRUE
MFSD2B	P3	0.01	0.04	0.02	4.97	0.000	0.000	TRUE
PTGER1	P3	0.01	0.03	0.01	3.77	0.000	0.000	TRUE
MFAP3L	P3	0.01	0.02	0.00	4.28	0.000	0.000	TRUE
TMEM132E	P3	0.01	0.05	0.03	2.70	0.007	0.016	TRUE
CADM2	P3	0.01	0.03	0.01	4.38	0.000	0.000	TRUE
TREML2	P3	0.01	0.03	0.02	2.69	0.007	0.017	TRUE
GABRD	P3	0.01	0.03	0.02	2.56	0.010	0.024	TRUE
EDA2R	P3	0.01	0.02	0.01	4.75	0.000	0.000	TRUE
CNNM2	P3	0.01	0.08	0.06	3.73	0.000	0.001	TRUE
SLC5A10	P3	0.01	0.02	0.01	2.61	0.009	0.021	TRUE
LRRC24	P3	0.01	0.05	0.03	3.56	0.000	0.001	TRUE
TLR3	P3	0.01	0.03	0.02	3.22	0.001	0.003	TRUE
MMEL1	P3	0.01	0.04	0.03	4.40	0.000	0.000	TRUE
GPC2	P3	0.01	0.03	0.01	3.47	0.001	0.001	TRUE
DUOX2	P3	0.01	0.01	0.00	2.69	0.007	0.017	TRUE
ITGB5	P3	0.01	0.02	0.01	3.85	0.000	0.000	TRUE
SLC38A11	P3	0.01	0.01	0.00	4.25	0.000	0.000	TRUE
PTH2R	P3	0.01	0.03	0.02	4.40	0.000	0.000	TRUE
MALRD1	P3	0.00	0.04	0.03	2.57	0.010	0.023	TRUE
THSD1	P3	0.00	0.03	0.02	2.85	0.004	0.011	TRUE
EFNA4	P3	0.00	0.05	0.04	3.35	0.001	0.002	TRUE
MANSC1	P3	0.00	0.03	0.02	4.22	0.000	0.000	TRUE
TAS2R14	P3	0.00	0.04	0.03	3.16	0.002	0.004	TRUE
SLC14A1	P3	0.00	0.04	0.03	3.03	0.002	0.006	TRUE
OR2C3	P3	0.00	0.01	0.01	2.49	0.013	0.029	TRUE
CD24	P3	0.00	0.02	0.01	2.66	0.008	0.018	TRUE
UNC5B	P3	0.00	0.01	0.01	2.69	0.007	0.017	TRUE

DISP2	P3	0.00	0.02	0.01	2.53	0.011	0.025	TRUE
PTGDR2	P3	0.00	0.01	0.00	3.23	0.001	0.003	TRUE
XKR3	P3	0.00	0.01	0.01	2.75	0.006	0.014	TRUE
BCAN	P3	0.00	0.01	0.00	3.20	0.001	0.003	TRUE
SLC52A3	P3	0.00	0.01	0.01	3.80	0.000	0.000	TRUE
PCDHGB2	P3	0.00	0.01	0.00	2.76	0.006	0.014	TRUE
PLXNB1	P3	0.00	0.02	0.02	2.90	0.004	0.009	TRUE
AMHR2	P3	0.00	0.00	0.00	3.36	0.001	0.002	TRUE
SLC26A1	P3	0.00	0.01	0.01	3.18	0.001	0.004	TRUE
SLC2A14	P3	0.00	0.02	0.01	4.01	0.000	0.000	TRUE
SCN4A	P3	0.00	0.01	0.00	2.64	0.008	0.019	TRUE
LGR6	P3	0.00	0.01	0.01	2.60	0.009	0.021	TRUE
IL5RA	P3	0.00	0.01	0.00	2.45	0.014	0.032	TRUE
SLC10A1	P3	0.00	0.01	0.00	3.37	0.001	0.002	TRUE
DUOXA1	P3	0.00	0.01	0.00	3.16	0.002	0.004	TRUE
CHRNA7	P3	0.00	0.01	0.01	2.46	0.014	0.031	TRUE
OR52H1	P3	0.00	0.00	0.00	2.59	0.010	0.022	TRUE
GLP1R	P3	0.00	0.00	0.00	2.40	0.017	0.036	TRUE
ADCY2	P3	0.00	0.01	0.00	2.31	0.021	0.045	TRUE
HRH4	P3	0.00	0.00	0.00	2.72	0.006	0.015	TRUE
GPR171	P3	0.00	0.10	0.07	3.32	0.001	0.002	TRUE
NIPAL1	P3	0.00	0.00	0.00	2.28	0.023	0.048	TRUE
LAMP3	P4	0.73	0.52	0.07	29.79	0.000	0.000	TRUE
CCR7	P4	0.49	0.45	0.23	24.27	0.000	0.000	TRUE
CD40	P4	0.44	0.59	0.36	25.95	0.000	0.000	TRUE
HLA-DPA1	P4	0.37	0.91	0.83	12.17	0.000	0.000	TRUE
HLA-DPB1	P4	0.32	0.91	0.85	8.87	0.000	0.000	TRUE
HLA-DQB1	P4	0.32	0.77	0.64	13.32	0.000	0.000	TRUE
CD74	P4	0.32	0.97	0.94	8.64	0.000	0.000	TRUE
SLAMF7	P4	0.30	0.31	0.10	19.56	0.000	0.000	TRUE
HLA-DRA	P4	0.29	0.95	0.93	10.40	0.000	0.000	TRUE
HLA-DQA1	P4	0.29	0.71	0.52	14.50	0.000	0.000	TRUE
VCAM1	P4	0.28	0.41	0.17	15.93	0.000	0.000	TRUE
IL2RA	P4	0.27	0.45	0.26	18.00	0.000	0.000	TRUE
ANTXR2	P4	0.26	0.67	0.41	18.78	0.000	0.000	TRUE
SLAMF1	P4	0.25	0.39	0.14	19.40	0.000	0.000	TRUE
HLA-DMB	P4	0.25	0.55	0.34	15.32	0.000	0.000	TRUE
ADAM28	P4	0.24	0.47	0.23	20.47	0.000	0.000	TRUE
AREG	P4	0.24	0.36	0.27	6.96	0.000	0.000	TRUE
PTGER4	P4	0.24	0.74	0.55	16.71	0.000	0.000	TRUE
LY75	P4	0.23	0.51	0.30	18.20	0.000	0.000	TRUE
SLC38A1	P4	0.23	0.77	0.58	11.80	0.000	0.000	TRUE
NRP2	P4	0.23	0.29	0.06	19.87	0.000	0.000	TRUE
SPPL2A	P4	0.22	0.70	0.49	16.53	0.000	0.000	TRUE
ALCAM	P4	0.22	0.51	0.28	17.94	0.000	0.000	TRUE
SLC5A3	P4	0.21	0.51	0.38	10.04	0.000	0.000	TRUE
IL7R	P4	0.20	0.25	0.08	17.90	0.000	0.000	TRUE
TMEM123	P4	0.20	0.89	0.78	10.58	0.000	0.000	TRUE
PTGIR	P4	0.20	0.47	0.35	11.67	0.000	0.000	TRUE
TSPAN33	P4	0.20	0.41	0.20	18.52	0.000	0.000	TRUE
IL3RA	P4	0.18	0.69	0.55	11.31	0.000	0.000	TRUE
CD274	P4	0.18	0.32	0.15	16.72	0.000	0.000	TRUE
SLCO5A1	P4	0.18	0.35	0.12	17.34	0.000	0.000	TRUE
CD58	P4	0.17	0.57	0.39	15.67	0.000	0.000	TRUE
TNFRSF9	P4	0.16	0.39	0.23	14.68	0.000	0.000	TRUE
FLT3	P4	0.16	0.42	0.27	14.28	0.000	0.000	TRUE
HLA-F	P4	0.16	0.58	0.46	10.66	0.000	0.000	TRUE
ITGB8	P4	0.15	0.18	0.05	14.42	0.000	0.000	TRUE
MPZL1	P4	0.14	0.51	0.32	14.01	0.000	0.000	TRUE
IL21R	P4	0.14	0.35	0.17	15.78	0.000	0.000	TRUE
FAS	P4	0.13	0.37	0.21	13.18	0.000	0.000	TRUE
IL15RA	P4	0.13	0.41	0.26	13.46	0.000	0.000	TRUE
LDLRAD4	P4	0.13	0.35	0.25	7.29	0.000	0.000	TRUE
IL6ST	P4	0.13	0.48	0.30	12.65	0.000	0.000	TRUE
SUCO	P4	0.12	0.59	0.42	11.07	0.000	0.000	TRUE
RHBDF2	P4	0.12	0.35	0.18	13.92	0.000	0.000	TRUE
TM9SF3	P4	0.12	0.83	0.70	6.78	0.000	0.000	TRUE
PMEPA1	P4	0.12	0.22	0.06	16.66	0.000	0.000	TRUE
IFNAR2	P4	0.12	0.52	0.33	13.02	0.000	0.000	TRUE
PVR	P4	0.12	0.23	0.08	15.24	0.000	0.000	TRUE
SLC15A4	P4	0.11	0.33	0.17	13.07	0.000	0.000	TRUE
CD80	P4	0.11	0.17	0.02	17.18	0.000	0.000	TRUE
LRP12	P4	0.11	0.46	0.33	11.46	0.000	0.000	TRUE
CD109	P4	0.11	0.38	0.27	8.37	0.000	0.000	TRUE
LNPEP	P4	0.11	0.60	0.44	8.30	0.000	0.000	TRUE
ICOSLG	P4	0.11	0.29	0.14	13.28	0.000	0.000	TRUE
CD1C	P4	0.11	0.10	0.01	9.21	0.000	0.000	TRUE
CD200	P4	0.11	0.22	0.09	12.92	0.000	0.000	TRUE
ADAM19	P4	0.10	0.23	0.09	14.67	0.000	0.000	TRUE
ITGB7	P4	0.10	0.20	0.10	7.25	0.000	0.000	TRUE
RELL1	P4	0.10	0.41	0.29	10.36	0.000	0.000	TRUE
CD70	P4	0.10	0.19	0.08	11.80	0.000	0.000	TRUE
SCARB2	P4	0.10	0.50	0.35	9.44	0.000	0.000	TRUE
TMEM30A	P4	0.10	0.61	0.46	7.71	0.000	0.000	TRUE
TM9SF2	P4	0.09	0.68	0.53	6.68	0.000	0.000	TRUE
IFNAR1	P4	0.09	0.57	0.43	8.71	0.000	0.000	TRUE
CLDND1	P4	0.09	0.62	0.47	8.01	0.000	0.000	TRUE
PLXND1	P4	0.09	0.36	0.22	11.62	0.000	0.000	TRUE
HLA-DOB	P4	0.09	0.15	0.03	16.07	0.000	0.000	TRUE
ECE1	P4	0.09	0.20	0.08	13.02	0.000	0.000	TRUE
SSTR2	P4	0.08	0.24	0.13	10.02	0.000	0.000	TRUE
LPAR6	P4	0.08	0.26	0.15	9.21	0.000	0.000	TRUE
TMEM106A	P4	0.08	0.38	0.24	9.59	0.000	0.000	TRUE
JAG1	P4	0.08	0.16	0.06	10.50	0.000	0.000	TRUE
BTN2A2	P4	0.08	0.34	0.21	8.91	0.000	0.000	TRUE
GJA4	P4	0.07	0.09	0.02	9.72	0.000	0.000	TRUE
CYSLTR1	P4	0.07	0.32	0.21	7.41	0.000	0.000	TRUE
LMBRD1	P4	0.07	0.39	0.27	7.81	0.000	0.000	TRUE
ANO9	P4	0.07	0.16	0.05	11.42	0.000	0.000	TRUE
HLA-DOA	P4	0.07	0.23	0.13	10.07	0.000	0.000	TRUE
TMEM87A	P4	0.06	0.55	0.42	6.31	0.000	0.000	TRUE

GRAMD1B	P4	0.06	0.19	0.08	10.75	0.000	0.000	TRUE
TGFBR1	P4	0.06	0.35	0.24	6.12	0.000	0.000	TRUE
SLC41A2	P4	0.06	0.15	0.04	14.41	0.000	0.000	TRUE
LMBR1L	P4	0.06	0.37	0.25	7.89	0.000	0.000	TRUE
DLL4	P4	0.06	0.08	0.01	10.23	0.000	0.000	TRUE
CLEC2D	P4	0.06	0.21	0.10	10.66	0.000	0.000	TRUE
TNFSF4	P4	0.06	0.18	0.11	7.32	0.000	0.000	TRUE
ACHE	P4	0.06	0.12	0.03	11.79	0.000	0.000	TRUE
ITGAV	P4	0.06	0.22	0.12	10.84	0.000	0.000	TRUE
ITGA1	P4	0.06	0.23	0.14	6.53	0.000	0.000	TRUE
CD1E	P4	0.06	0.07	0.00	6.80	0.000	0.000	TRUE
CRLF2	P4	0.05	0.17	0.11	6.97	0.000	0.000	TRUE
BTN2A1	P4	0.05	0.30	0.18	8.00	0.000	0.000	TRUE
GPR157	P4	0.05	0.09	0.02	11.02	0.000	0.000	TRUE
GPR137B	P4	0.05	0.11	0.03	12.20	0.000	0.000	TRUE
GPR107	P4	0.05	0.28	0.18	9.51	0.000	0.000	TRUE
CALCRL	P4	0.05	0.15	0.08	8.08	0.000	0.000	TRUE
SCARF1	P4	0.05	0.26	0.17	7.80	0.000	0.000	TRUE
LILRA4	P4	0.05	0.06	0.00	8.09	0.000	0.000	TRUE
P2RY10	P4	0.05	0.14	0.07	11.03	0.000	0.000	TRUE
BMPR2	P4	0.05	0.20	0.11	10.32	0.000	0.000	TRUE
MR1	P4	0.05	0.29	0.21	7.15	0.000	0.000	TRUE
PDGFRA	P4	0.05	0.14	0.05	8.81	0.000	0.000	TRUE
PTCRA	P4	0.05	0.04	0.00	5.79	0.000	0.000	TRUE
PLXNC1	P4	0.05	0.22	0.13	8.52	0.000	0.000	TRUE
CRIM1	P4	0.04	0.14	0.08	7.87	0.000	0.000	TRUE
TNFRSF11A	P4	0.04	0.09	0.01	13.79	0.000	0.000	TRUE
SLC2A1	P4	0.04	0.24	0.17	5.34	0.000	0.000	TRUE
ITGA6	P4	0.04	0.26	0.18	6.63	0.000	0.000	TRUE
MS4A1	P4	0.04	0.04	0.00	8.50	0.000	0.000	TRUE
LYSMD3	P4	0.04	0.33	0.25	4.21	0.000	0.000	TRUE
ENPP4	P4	0.04	0.13	0.08	3.80	0.000	0.000	TRUE
LAG3	P4	0.04	0.22	0.15	2.93	0.003	0.008	TRUE
TPBG	P4	0.04	0.18	0.11	5.84	0.000	0.000	TRUE
NIPAL3	P4	0.04	0.22	0.15	6.16	0.000	0.000	TRUE
TMEM87B	P4	0.04	0.30	0.22	6.66	0.000	0.000	TRUE
NEGR1	P4	0.04	0.08	0.04	3.69	0.000	0.001	TRUE
SSPN	P4	0.04	0.11	0.04	8.05	0.000	0.000	TRUE
SDK2	P4	0.03	0.09	0.03	6.25	0.000	0.000	TRUE
ADCY9	P4	0.03	0.11	0.05	6.59	0.000	0.000	TRUE
GPRC5C	P4	0.03	0.20	0.15	3.95	0.000	0.000	TRUE
P2RY14	P4	0.03	0.12	0.06	7.10	0.000	0.000	TRUE
MFSD11	P4	0.03	0.25	0.17	6.28	0.000	0.000	TRUE
SLC8A3	P4	0.03	0.09	0.02	8.42	0.000	0.000	TRUE
ACVR2A	P4	0.03	0.08	0.02	8.94	0.000	0.000	TRUE
PAM	P4	0.03	0.12	0.06	8.41	0.000	0.000	TRUE
TSPAN15	P4	0.03	0.05	0.01	9.26	0.000	0.000	TRUE
ERVK13-1	P4	0.03	0.16	0.10	6.37	0.000	0.000	TRUE
ACVRL1	P4	0.03	0.08	0.03	8.00	0.000	0.000	TRUE
PRRT3	P4	0.03	0.10	0.05	5.52	0.000	0.000	TRUE
SCN1B	P4	0.03	0.09	0.04	7.58	0.000	0.000	TRUE
SLC6A12	P4	0.03	0.05	0.00	9.14	0.000	0.000	TRUE
KIAA1324L	P4	0.03	0.12	0.07	5.97	0.000	0.000	TRUE
FLVCR2	P4	0.03	0.07	0.02	7.67	0.000	0.000	TRUE
PTPRM	P4	0.03	0.12	0.08	2.99	0.003	0.007	TRUE
CHL1	P4	0.03	0.05	0.00	5.13	0.000	0.000	TRUE
ACKR1	P4	0.03	0.06	0.02	5.83	0.000	0.000	TRUE
EMP2	P4	0.03	0.08	0.04	5.90	0.000	0.000	TRUE
CXCR3	P4	0.03	0.07	0.03	6.46	0.000	0.000	TRUE
CD226	P4	0.02	0.06	0.02	6.84	0.000	0.000	TRUE
SIDT2	P4	0.02	0.13	0.09	4.00	0.000	0.000	TRUE
KCNMB1	P4	0.02	0.08	0.04	6.50	0.000	0.000	TRUE
SLC12A7	P4	0.02	0.19	0.14	3.65	0.000	0.001	TRUE
MC2R	P4	0.02	0.05	0.00	5.13	0.000	0.000	TRUE
CCR6	P4	0.02	0.04	0.00	6.00	0.000	0.000	TRUE
LY9	P4	0.02	0.04	0.01	4.37	0.000	0.000	TRUE
MPZL3	P4	0.02	0.08	0.03	5.05	0.000	0.000	TRUE
GABBR1	P4	0.02	0.08	0.03	8.36	0.000	0.000	TRUE
ABCA5	P4	0.02	0.09	0.05	3.80	0.000	0.000	TRUE
PGAP1	P4	0.02	0.12	0.07	4.98	0.000	0.000	TRUE
CLSTN3	P4	0.02	0.15	0.09	5.30	0.000	0.000	TRUE
DPP4	P4	0.02	0.09	0.03	6.94	0.000	0.000	TRUE
PLXNA4	P4	0.02	0.04	0.01	2.87	0.004	0.010	TRUE
TTYH2	P4	0.02	0.09	0.05	6.32	0.000	0.000	TRUE
ABCA7	P4	0.02	0.10	0.06	2.91	0.004	0.009	TRUE
PDCD1LG2	P4	0.02	0.07	0.02	6.87	0.000	0.000	TRUE
AVPR1B	P4	0.02	0.05	0.00	3.49	0.000	0.001	TRUE
LRRC37A2	P4	0.02	0.11	0.06	4.23	0.000	0.000	TRUE
FZD6	P4	0.02	0.17	0.12	2.40	0.016	0.036	TRUE
SEMA4C	P4	0.02	0.06	0.02	7.12	0.000	0.000	TRUE
SLC41A1	P4	0.02	0.09	0.04	7.13	0.000	0.000	TRUE
EFNB1	P4	0.02	0.10	0.06	5.26	0.000	0.000	TRUE
SIDT1	P4	0.02	0.07	0.04	4.31	0.000	0.000	TRUE
GPR34	P4	0.02	0.03	0.01	2.74	0.006	0.014	TRUE
LYPD6B	P4	0.02	0.05	0.00	5.13	0.000	0.000	TRUE
LRIG2	P4	0.02	0.14	0.09	3.65	0.000	0.001	TRUE
NPFFR1	P4	0.02	0.03	0.00	5.73	0.000	0.000	TRUE
MPZL2	P4	0.02	0.06	0.03	2.59	0.009	0.022	TRUE
CEACAM19	P4	0.02	0.05	0.01	5.30	0.000	0.000	TRUE
CACNG8	P4	0.02	0.09	0.06	3.55	0.000	0.001	TRUE
SLC28A1	P4	0.02	0.03	0.00	3.51	0.000	0.001	TRUE
SLC9A6	P4	0.02	0.10	0.07	2.85	0.004	0.010	TRUE
HLA-DQB2	P4	0.02	0.05	0.02	4.26	0.000	0.000	TRUE
GFRA2	P4	0.02	0.03	0.00	3.51	0.000	0.001	TRUE
CDH1	P4	0.02	0.02	0.00	3.08	0.002	0.005	TRUE
GPR55	P4	0.02	0.03	0.01	5.95	0.000	0.000	TRUE
CLDN12	P4	0.01	0.06	0.03	5.73	0.000	0.000	TRUE
SLC26A9	P4	0.01	0.03	0.00	6.12	0.000	0.000	TRUE
CEACAM21	P4	0.01	0.07	0.04	4.50	0.000	0.000	TRUE
PTGFRN	P4	0.01	0.04	0.01	5.27	0.000	0.000	TRUE
IL18RAP	P4	0.01	0.04	0.01	2.68	0.007	0.017	TRUE

TMPRSS13	P4	0.01	0.04	0.01	5.42	0.000	0.000	TRUE
ITGAD	P4	0.01	0.03	0.00	3.13	0.002	0.004	TRUE
LAYN	P4	0.01	0.04	0.02	3.99	0.000	0.000	TRUE
TNFRSF21	P4	0.01	0.06	0.03	2.94	0.003	0.008	TRUE
TMEM231	P4	0.01	0.06	0.03	2.85	0.004	0.011	TRUE
CNR2	P4	0.01	0.04	0.02	3.22	0.001	0.003	TRUE
HCAR1	P4	0.01	0.06	0.03	2.79	0.005	0.013	TRUE
TNFSF15	P4	0.01	0.02	0.01	5.65	0.000	0.000	TRUE
AXL	P4	0.01	0.04	0.01	3.84	0.000	0.000	TRUE
FZD4	P4	0.01	0.02	0.00	4.31	0.000	0.000	TRUE
TMEM132A	P4	0.01	0.03	0.01	5.67	0.000	0.000	TRUE
AJAP1	P4	0.01	0.02	0.00	2.81	0.005	0.012	TRUE
PIGR	P4	0.01	0.02	0.00	3.25	0.001	0.003	TRUE
EGF	P4	0.01	0.04	0.02	2.74	0.006	0.014	TRUE
PANX2	P4	0.01	0.17	0.12	4.64	0.000	0.000	TRUE
IGSF3	P4	0.01	0.02	0.00	4.60	0.000	0.000	TRUE
FKRP	P4	0.01	0.10	0.07	4.08	0.000	0.000	TRUE
ART3	P4	0.01	0.03	0.01	3.12	0.002	0.005	TRUE
CXCR5	P4	0.01	0.03	0.01	6.09	0.000	0.000	TRUE
PTPRS	P4	0.01	0.04	0.02	3.02	0.002	0.006	TRUE
BTLA	P4	0.01	0.02	0.00	3.28	0.001	0.003	TRUE
SCARF2	P4	0.01	0.02	0.00	3.15	0.002	0.004	TRUE
CEACAM1	P4	0.01	0.04	0.02	5.43	0.000	0.000	TRUE
BCAM	P4	0.01	0.04	0.02	2.60	0.009	0.021	TRUE
GPR82	P4	0.01	0.03	0.01	3.15	0.002	0.004	TRUE
PTCHD4	P4	0.01	0.01	0.00	2.83	0.005	0.011	TRUE
SLC8A2	P4	0.01	0.02	0.00	3.71	0.000	0.001	TRUE
ACKR3	P4	0.01	0.02	0.01	4.78	0.000	0.000	TRUE
DRD4	P4	0.01	0.02	0.00	2.74	0.006	0.015	TRUE
GJC2	P4	0.01	0.02	0.00	2.45	0.014	0.031	TRUE
LSMEM1	P4	0.01	0.05	0.03	3.12	0.002	0.005	TRUE
FCRL5	P4	0.01	0.01	0.00	2.30	0.022	0.046	TRUE
PTPRF	P4	0.01	0.02	0.00	3.87	0.000	0.000	TRUE
SEMA5B	P4	0.01	0.02	0.00	2.96	0.003	0.008	TRUE
PROCR	P4	0.01	0.06	0.04	3.00	0.003	0.007	TRUE
GALR2	P4	0.01	0.02	0.00	3.78	0.000	0.000	TRUE
TMEM255A	P4	0.01	0.02	0.00	5.23	0.000	0.000	TRUE
CNNM4	P4	0.01	0.11	0.08	3.43	0.001	0.002	TRUE
SLC22A13	P4	0.01	0.02	0.00	4.74	0.000	0.000	TRUE
CD5	P4	0.01	0.03	0.02	3.13	0.002	0.005	TRUE
PLVAP	P4	0.01	0.02	0.00	5.10	0.000	0.000	TRUE
EFNA5	P4	0.01	0.02	0.00	2.96	0.003	0.007	TRUE
SLC23A1	P4	0.01	0.02	0.01	3.01	0.003	0.006	TRUE
DUOX1	P4	0.01	0.03	0.02	4.00	0.000	0.000	TRUE
CHRNA6	P4	0.01	0.03	0.01	3.32	0.001	0.002	TRUE
GRIN3B	P4	0.01	0.02	0.00	2.76	0.006	0.014	TRUE
TLR10	P4	0.01	0.02	0.00	3.37	0.001	0.002	TRUE
HEPHL1	P4	0.01	0.01	0.00	2.80	0.005	0.012	TRUE
EPHA2	P4	0.01	0.01	0.00	3.83	0.000	0.000	TRUE
RTN4RL1	P4	0.01	0.02	0.01	2.84	0.004	0.011	TRUE
OXTR	P4	0.01	0.02	0.00	4.00	0.000	0.000	TRUE
TNFSF18	P4	0.01	0.01	0.00	2.78	0.005	0.013	TRUE
GP5	P4	0.01	0.01	0.00	3.12	0.002	0.005	TRUE
SLC4A5	P4	0.01	0.04	0.02	2.92	0.003	0.008	TRUE
LRP1B	P4	0.01	0.01	0.00	3.03	0.002	0.006	TRUE
MFAP3	P4	0.01	0.05	0.04	2.39	0.017	0.036	TRUE
OMG	P4	0.01	0.02	0.00	3.65	0.000	0.001	TRUE
NTNG1	P4	0.01	0.02	0.00	3.96	0.000	0.000	TRUE
DLL1	P4	0.01	0.03	0.01	2.39	0.017	0.037	TRUE
CDH2	P4	0.01	0.02	0.00	2.93	0.003	0.008	TRUE
GPR153	P4	0.01	0.01	0.00	4.45	0.000	0.000	TRUE
PARM1	P4	0.01	0.01	0.00	2.44	0.015	0.032	TRUE
TMEFF1	P4	0.01	0.02	0.01	3.65	0.000	0.001	TRUE
PTPRN	P4	0.01	0.01	0.00	2.30	0.022	0.046	TRUE
CSPG4	P4	0.01	0.01	0.00	3.73	0.000	0.001	TRUE
LHFPL4	P4	0.01	0.03	0.02	2.35	0.019	0.041	TRUE
CLDN9	P4	0.01	0.01	0.00	2.63	0.009	0.020	TRUE
GGT7	P4	0.01	0.06	0.05	2.53	0.011	0.025	TRUE
SLC37A3	P4	0.01	0.05	0.03	2.32	0.020	0.044	TRUE
ITGA3	P4	0.01	0.03	0.02	3.49	0.000	0.001	TRUE
CD6	P4	0.01	0.04	0.03	4.09	0.000	0.000	TRUE
ADAM11	P4	0.01	0.02	0.01	3.78	0.000	0.000	TRUE
NOTCH3	P4	0.01	0.02	0.01	2.84	0.005	0.011	TRUE
SLC12A3	P4	0.01	0.01	0.00	4.09	0.000	0.000	TRUE
NLGN3	P4	0.00	0.03	0.01	2.57	0.010	0.023	TRUE
SYP	P4	0.00	0.03	0.02	2.61	0.009	0.021	TRUE
VASN	P4	0.00	0.01	0.00	2.79	0.005	0.013	TRUE
CNTNAP2	P4	0.00	0.01	0.00	2.45	0.014	0.032	TRUE
DDR1	P4	0.00	0.01	0.00	3.88	0.000	0.000	TRUE
AOC3	P4	0.00	0.01	0.00	2.91	0.004	0.009	TRUE
CD8A	P4	0.00	0.01	0.00	3.75	0.000	0.001	TRUE
NLGN4Y	P4	0.00	0.01	0.00	3.37	0.001	0.002	TRUE
CDON	P4	0.00	0.01	0.00	4.13	0.000	0.000	TRUE
RNF43	P4	0.00	0.06	0.04	2.36	0.018	0.039	TRUE
SLC6A16	P4	0.00	0.01	0.00	3.09	0.002	0.005	TRUE
FAM171B	P4	0.00	0.02	0.01	2.72	0.007	0.015	TRUE
EFNB3	P4	0.00	0.02	0.01	2.50	0.012	0.028	TRUE
PTPRK	P4	0.00	0.01	0.00	3.16	0.002	0.004	TRUE
SIGLEC15	P4	0.00	0.03	0.02	2.31	0.021	0.045	TRUE
GPR155	P4	0.00	0.03	0.02	3.24	0.001	0.003	TRUE
GPR173	P4	0.00	0.02	0.01	2.34	0.019	0.041	TRUE
PCDH12	P4	0.00	0.01	0.00	3.09	0.002	0.005	TRUE
SLC1A2	P4	0.00	0.02	0.01	2.43	0.015	0.033	TRUE
UPK2	P4	0.00	0.01	0.00	2.82	0.005	0.011	TRUE
ILDR1	P4	0.00	0.01	0.00	2.91	0.004	0.009	TRUE
TAS1R3	P4	0.00	0.01	0.00	2.95	0.003	0.008	TRUE
CDH13	P4	0.00	0.01	0.00	2.44	0.015	0.032	TRUE
GPC5	P4	0.00	0.01	0.00	2.39	0.017	0.037	TRUE
CDHR3	P4	0.00	0.01	0.00	2.89	0.004	0.009	TRUE
CD248	P4	0.00	0.02	0.01	3.05	0.002	0.006	TRUE
LRRTM2	P4	0.00	0.02	0.01	2.94	0.003	0.008	TRUE

SLC6A7	P4	0.00	0.01	0.00	3.56	0.000	0.001	TRUE
GPM6B	P4	0.00	0.05	0.04	2.69	0.007	0.017	TRUE
JAG2	P4	0.00	0.01	0.00	2.76	0.006	0.014	TRUE
CADM1	P4	0.00	0.01	0.00	2.32	0.021	0.044	TRUE
TRABD2A	P4	0.00	0.02	0.01	2.92	0.004	0.009	TRUE
SEZ6	P4	0.00	0.03	0.02	2.55	0.011	0.024	TRUE
DLK2	P4	0.00	0.01	0.00	2.37	0.018	0.039	TRUE
TLR9	P4	0.00	0.01	0.01	3.42	0.001	0.002	TRUE
ROR2	P4	0.00	0.01	0.00	3.57	0.000	0.001	TRUE
PCDHGB7	P4	0.00	0.01	0.00	3.80	0.000	0.000	TRUE
MST1R	P4	0.00	0.01	0.00	2.65	0.008	0.019	TRUE
ATP2B2	P4	0.00	0.01	0.00	2.62	0.009	0.020	TRUE
IGDCC4	P4	0.00	0.01	0.00	2.58	0.010	0.023	TRUE
TIGIT	P4	0.00	0.01	0.01	3.27	0.001	0.003	TRUE
CD27	P4	0.00	0.01	0.00	2.68	0.007	0.017	TRUE
TGFBR3	P4	0.00	0.01	0.00	3.34	0.001	0.002	TRUE
PDCD1	P4	0.00	0.01	0.00	2.27	0.023	0.049	TRUE
CNTNAP1	P4	0.00	0.01	0.00	2.96	0.003	0.008	TRUE
ABCB1	P4	0.00	0.01	0.01	2.95	0.003	0.008	TRUE
PCDHGB6	P4	0.00	0.01	0.00	2.93	0.003	0.008	TRUE
LIFR	P4	0.00	0.01	0.00	2.27	0.023	0.050	TRUE
ERVW-1	P4	0.00	0.00	0.00	2.37	0.018	0.039	TRUE
OR1I1	P4	0.00	0.01	0.00	2.83	0.005	0.011	TRUE
SLC26A5	P4	0.00	0.00	0.00	2.69	0.007	0.017	TRUE
CD160	P4	0.00	0.02	0.02	2.40	0.016	0.036	TRUE
IL17RD	P4	0.00	0.00	0.00	3.46	0.001	0.001	TRUE

Table S4. Abundance of AML programs in hematopoietic cell lines, related to **Figure 2**.

Cell line	AML program			
	P1	P2	P3	P4
PL21	0.42	0.03	0.15	0.02
MONOMAC1	0.41	0.04	0.28	0.10
NOMO1	0.41	0.04	0.22	0.08
OCIAML2	0.40	0.06	0.26	0.11
SIGM5	0.38	0.10	0.14	0.02
OCIAML3	0.37	0.00	0.13	0.05
OCIAML5	0.35	0.04	0.32	0.08
EOL1	0.35	0.03	0.28	0.08
THP1	0.33	0.16	0.29	0.15
SKM1	0.32	0.14	0.27	0.11
MV411	0.31	0.08	0.20	0.01
MOLM13	0.28	0.07	0.25	0.02
PLB985	0.20	0.00	0.07	0.01
HL60	0.11	0.04	0.08	0.00
REH	0.02	0.37	0.29	0.27
SUPT1	0.01	0.34	0.31	0.30
BL41	0.05	0.33	0.13	0.18
ST486	0.01	0.32	0.26	0.30
CI1	0.09	0.31	0.16	0.25
GA10	0.02	0.30	0.11	0.22
RAJI	0.04	0.30	0.13	0.21
P3HR1	0.08	0.27	0.18	0.26
MINO	0.10	0.26	0.23	0.25
BL70	0.02	0.26	0.07	0.14
697	0.03	0.20	0.14	0.19
M07E	0.07	0.04	0.68	0.07
KU812	0.05	0.08	0.61	0.07
HEL	0.09	0.07	0.61	0.07
CMK	0.14	0.05	0.59	0.08
MOLM16	0.07	0.08	0.59	0.10
SET2	0.10	0.07	0.57	0.14
KASUMI6	0.13	0.01	0.55	0.10
KG1	0.08	0.19	0.53	0.17
HEL9217	0.05	0.21	0.52	0.13
NCO2	0.06	0.07	0.50	0.13
KARPAS422	0.09	0.08	0.49	0.34
TF1	0.10	0.19	0.46	0.11
MOLT16	0.09	0.04	0.46	0.30
OCIM1	0.07	0.02	0.46	0.15
MUTZ3	0.26	0.00	0.45	0.24
KO52	0.09	0.07	0.45	0.08
GDM1	0.10	0.05	0.44	0.21
KCL22	0.09	0.19	0.44	0.22
CMLT1	0.07	0.05	0.44	0.36
P31FUJ	0.26	0.13	0.42	0.20
JURLMK1	0.12	0.06	0.42	0.14
LP1	0.07	0.00	0.42	0.41
SKNO1	0.02	0.21	0.42	0.08

K562	0.09	0.12	0.42	0.21
LOUCY	0.03	0.24	0.41	0.29
MEG01	0.07	0.07	0.40	0.06
RPMI8402	0.00	0.21	0.40	0.25
AML193	0.28	0.16	0.39	0.09
CA46	0.03	0.08	0.39	0.38
HNT34	0.08	0.01	0.39	0.19
MOLT13	0.03	0.21	0.39	0.31
F36P	0.12	0.01	0.38	0.12
PEER	0.00	0.27	0.38	0.14
MONOMAC6	0.27	0.19	0.37	0.15
SKMM2	0.06	0.07	0.36	0.21
DND41	0.04	0.20	0.36	0.32
A4FUK	0.11	0.13	0.36	0.25
JURKAT	0.04	0.22	0.35	0.31
P12ICHIKAWA	0.02	0.27	0.35	0.26
SUPT11	0.05	0.23	0.35	0.32
A3KAW	0.11	0.01	0.35	0.24
LAMA84	0.12	0.01	0.34	0.09
MOLP8	0.11	0.02	0.34	0.31
OPM2	0.10	0.02	0.34	0.28
EJM	0.12	0.13	0.34	0.33
KMS27	0.11	0.09	0.34	0.20
BCP1	0.08	0.13	0.34	0.23
HUT78	0.06	0.28	0.34	0.29
ALLSIL	0.02	0.26	0.33	0.26
EM2	0.21	0.17	0.33	0.08
KMS28BM	0.07	0.13	0.32	0.23
JJN3	0.09	0.13	0.32	0.27
KASUMI1	0.09	0.09	0.32	0.11
ME1	0.04	0.02	0.31	0.03
U937	0.29	0.11	0.31	0.06
KMS34	0.07	0.19	0.31	0.20
MOLT3	0.02	0.15	0.30	0.29
MOLP2	0.15	0.01	0.30	0.21
NB4	0.18	0.24	0.30	0.04
MOLM6	0.14	0.01	0.30	0.04
JK1	0.11	0.00	0.29	0.09
KHM1B	0.08	0.09	0.29	0.13
KMS21BM	0.10	0.03	0.29	0.19
KMS12BM	0.05	0.20	0.28	0.18
KYO1	0.05	0.04	0.26	0.04
RL	0.07	0.19	0.26	0.19
KOPN8	0.13	0.16	0.25	0.18
NCIH929	0.09	0.03	0.24	0.15
AMO1	0.07	0.13	0.22	0.16
BV173	0.01	0.11	0.19	0.19
RPMI8226	0.12	0.11	0.18	0.16
RS411	0.02	0.10	0.16	0.01
OCIMY7	0.05	0.00	0.15	0.11
JVM2	0.17	0.00	0.16	0.51
HUT102	0.14	0.00	0.21	0.50

EB1	0.06	0.03	0.34	0.50
HDLM2	0.09	0.09	0.25	0.49
HS611T	0.11	0.11	0.21	0.48
EHEB	0.16	0.00	0.12	0.48
JVM3	0.16	0.00	0.14	0.46
GRANTA519	0.08	0.07	0.24	0.45
SR786	0.14	0.07	0.30	0.43
L1236	0.11	0.10	0.27	0.43
SUPM2	0.16	0.05	0.23	0.40
L363	0.05	0.12	0.39	0.40
MHHCALL4	0.06	0.14	0.33	0.40
DEL	0.18	0.01	0.24	0.39
SUPB15	0.04	0.24	0.27	0.39
KMS26	0.05	0.13	0.35	0.39
MHHCALL3	0.05	0.24	0.27	0.39
KMH2	0.10	0.07	0.13	0.37
PF382	0.03	0.23	0.26	0.37
SUDHL5	0.05	0.26	0.18	0.36
MUTZ5	0.04	0.07	0.21	0.36
KARPAS299	0.23	0.04	0.21	0.36
TOLEDO	0.02	0.32	0.22	0.36
L540	0.12	0.05	0.23	0.36
MJ	0.06	0.23	0.29	0.36
OPM1	0.07	0.05	0.18	0.36
MEC1	0.11	0.07	0.19	0.35
PFEIFFER	0.03	0.20	0.30	0.35
KASUMI2	0.05	0.17	0.28	0.35
HDMYZ	0.10	0.06	0.31	0.35
BDCM	0.09	0.01	0.13	0.34
NALM1	0.03	0.11	0.29	0.34
KMS20	0.10	0.20	0.34	0.34
OCILY3	0.09	0.12	0.11	0.34
U266B1	0.09	0.17	0.32	0.34
NALM6	0.03	0.21	0.22	0.34
HUNS1	0.05	0.17	0.15	0.34
REC1	0.10	0.12	0.27	0.34
HT	0.07	0.23	0.28	0.33
DAUDI	0.08	0.07	0.17	0.33
NALM19	0.03	0.25	0.31	0.33
KMS11	0.09	0.21	0.29	0.33
JM1	0.01	0.29	0.20	0.33
C8166	0.10	0.12	0.18	0.32
MHHCALL2	0.04	0.27	0.28	0.32
KE37	0.03	0.23	0.29	0.32
HPBALL	0.03	0.19	0.26	0.32
TALL1	0.01	0.22	0.30	0.32
KMM1	0.04	0.25	0.30	0.32
RI1	0.08	0.18	0.18	0.32
SUDHL6	0.04	0.20	0.22	0.32
INA6	0.08	0.10	0.29	0.31
DB	0.03	0.18	0.31	0.31
SEM	0.07	0.24	0.28	0.30

NUDUL1	0.07	0.18	0.18	0.30
NAMALWA	0.04	0.21	0.26	0.29
KE97	0.06	0.12	0.16	0.29
L428	0.05	0.12	0.15	0.29
MM1S	0.14	0.22	0.20	0.28
HH	0.07	0.21	0.24	0.28
SUDHL1	0.10	0.13	0.26	0.27
KARPAS620	0.11	0.14	0.25	0.27
WSUDLCL2	0.05	0.06	0.22	0.26
MC116	0.04	0.19	0.17	0.25
OCILY19	0.04	0.05	0.19	0.24
KMS18	0.06	0.02	0.16	0.23
DOHH2	0.08	0.04	0.13	0.21
RCHACV	0.06	0.20	0.20	0.21
KIJK	0.18	0.10	0.16	0.21
OCIMY5	0.07	0.03	0.19	0.20
SUDHL8	0.01	0.19	0.16	0.20
JEKO1	0.09	0.15	0.17	0.19
SUDHL10	0.07	0.10	0.18	0.18
NUDHL1	0.16	0.00	0.16	0.17
SUDHL4	0.08	0.03	0.15	0.15

Table S5. $CD4^{IL10}$ marker genes significantly changing in $CD4^{IL10}$ cells due to co-culture with sensitive or resistant blasts, related to **Figure 3**.

Gene symbol - The symbol of gene for which the differential expression is assessed.

Average log2 fold change - The weighted average log2 fold change of gene in CD4IL10 cells after 24 hour co-culture with AML sensitive or resistant to killing (**Methods**).

DE meta z-score - The meta z-score obtained by aggregating individual differntial expression (DE) z-score statistics of CD4IL10 cells after 24 hour co-culture with AML sensitive or resistant to killing, using Liptak's method, across CD4IL10 donors (**Methods**).

-log10 DE *p*-value - The -log10 of the p-value obtained by converting the value in column **DE meta z-score** to a two-sided *p*-value. Sign indicates the dirrection of differential expression, <0 - under-expressed, >0 - over-expressed.

-log10 DE *q*-value - The **-lo**g10 *q*-value corresponding to the *p*-value in column **-log10 DE *p*-value**, after adjusted for multiple hypothesis testing using Benjamini-Hochberg method. Sign indicates the dirrection of differential expression, <0 - under-expressed, >0 - over-expressed.

AML association - Flag indicating wgether the gene is significantly over-expressed in CD4IL10 co-cutured with either sensitive or resistant AML.

Suface protein - Flag indicating whether the gene is a surface protein, as annotated in Cell Surface Protein Atlas (*Bausch-Fluck et al. 2018*)**.**

Note: For columns B,C,F,G the the natural log of the number of CD4IL10 cells from each CD4IL10 donor before and after co-culture with each AML patient were used as weights.

	$CD4^{IL10}$ co-cutured with sensitive AML				$CD4^{IL10}$ co-cutured with resistant AML					
**Gene symbol**	**Average log_2 fold change**	**DE meta z-score**	**$-log_{10}$ DE *p*-value**	**$-log_{10}$ DE *q*-value**	**Average log_2 fold change**	**DE meta z-score**	**$-log_{10}$ DE *p*-value**	**$-log_{10}$ DE *q*-value**	**AML association**	**Surface protein**
IL2RA	0.85	23.40	120.42	118.08	-0.26	-12.72	-36.32	-34.73	Sensitive	TRUE
TNFRSF18	0.72	23.95	126.03	123.66	-0.07	-5.51	-7.44	-6.53	Sensitive	TRUE
TNFRSF4	0.62	17.40	67.12	65.19	-0.14	-8.37	-16.24	-15.06	Sensitive	TRUE
HAVCR2	0.41	15.70	54.84	53.03	-0.07	-3.37	-3.12	-2.47	Sensitive	TRUE
SLAMF7	0.38	13.02	38.02	36.40	0.09	0.59	0.26	0.03	Sensitive	TRUE
IL3RA	0.37	13.31	39.69	38.05	0.06	-2.31	-1.68	-1.17	Sensitive	TRUE
CSF1	0.34	11.62	30.48	29.00	-0.04	-5.91	-8.46	-7.51	Sensitive	TRUE
CD38	0.33	11.67	30.73	29.25	-0.13	-11.33	-29.01	-27.56	Sensitive	TRUE
IL12RB2	0.30	14.96	49.90	48.13	-0.03	-5.67	-7.83	-6.91	Sensitive	TRUE
CTLA4	0.29	14.42	46.40	44.67	-0.01	-4.71	-5.60	-4.79	Sensitive	TRUE
SLAMF1	0.28	11.11	27.93	26.49	0.04	-1.25	-0.67	-0.32	Sensitive	TRUE
CD83	0.27	21.85	105.12	102.88	0.04	2.37	1.75	1.23	Sensitive	TRUE
ADAM19	0.25	12.05	32.72	31.20	-0.20	-8.09	-15.24	-14.07	Sensitive	TRUE
TCTN3	0.25	9.08	18.98	17.73	0.06	-1.06	-0.54	-0.22	Sensitive	TRUE
ICAM1	0.25	18.16	73.00	71.02	0.03	0.58	0.25	0.03	Sensitive	TRUE
CD70	0.24	11.41	29.44	27.98	0.04	-2.69	-2.15	-1.59	Sensitive	TRUE
LRP8	0.19	11.94	32.12	30.61	-0.02	-4.30	-4.77	-4.00	Sensitive	TRUE
SPPL2A	0.18	6.78	10.92	9.88	0.04	0.23	0.09	0.00	Sensitive	TRUE
PGAP1	0.18	10.75	26.24	24.84	0.05	0.98	0.49	0.20	Sensitive	TRUE
CSF2RB	0.18	6.38	9.75	8.75	0.00	-3.88	-3.99	-3.27	Sensitive	TRUE
TNFRSF8	0.16	11.74	31.10	29.60	0.01	-3.73	-3.72	-3.02	Sensitive	TRUE
SLC38A5	0.15	8.67	17.36	16.15	0.02	-0.09	-0.03	0.00	Sensitive	TRUE
SLC7A5	0.14	7.48	13.12	12.01	-0.03	-5.57	-7.60	-6.69	Sensitive	TRUE
FASLG	0.14	11.69	30.83	29.34	0.04	2.37	1.75	1.23	Sensitive	TRUE
ATP6V0A2	0.13	6.54	10.21	9.19	0.04	-0.13	-0.05	0.00	Sensitive	TRUE
CD274	0.12	11.75	31.15	29.65	0.02	0.86	0.41	0.14	Sensitive	TRUE

SEMA4A	0.12	13.93	43.36	41.67	0.02	2.01	1.35	0.88	Sensitive	TRUE
FURIN	0.12	4.85	5.91	5.07	-0.15	-7.73	-13.98	-12.85	Sensitive	TRUE
NOTCH1	0.11	5.27	6.86	5.98	0.01	-0.84	-0.39	-0.13	Sensitive	TRUE
HEG1	0.11	5.83	8.25	7.31	0.01	-0.54	-0.23	-0.03	Sensitive	TRUE
PTGIR	0.10	13.80	42.61	40.93	0.01	1.99	1.33	0.87	Sensitive	TRUE
ECE1	0.10	5.01	6.27	5.42	0.04	-0.19	-0.07	0.00	Sensitive	TRUE
TMEM178B	0.09	5.97	8.63	7.67	0.02	-1.00	-0.50	-0.20	Sensitive	TRUE
IL15RA	0.09	8.45	16.55	15.36	0.02	-0.84	-0.40	-0.13	Sensitive	TRUE
GPR137	0.08	6.68	10.61	9.58	0.03	0.37	0.15	0.00	Sensitive	TRUE
NOTCH2	0.08	2.50	1.91	1.36	-0.04	-4.67	-5.53	-4.72	Sensitive	TRUE
APLP2	0.07	2.69	2.15	1.59	-0.02	-4.60	-5.37	-4.57	Sensitive	TRUE
IL6ST	0.06	2.64	2.09	1.53	-0.01	-5.85	-8.31	-7.37	Sensitive	TRUE
CCR4	0.05	6.22	9.30	8.32	0.00	-2.52	-1.94	-1.39	Sensitive	TRUE
NETO2	0.05	6.82	11.05	10.01	-0.01	-3.33	-3.06	-2.41	Sensitive	TRUE
MFSD12	0.05	3.26	2.95	2.31	0.01	-1.80	-1.14	-0.71	Sensitive	TRUE
PKD2	0.05	5.31	6.96	6.08	0.01	-0.59	-0.26	-0.03	Sensitive	TRUE
ERVK13-1	0.05	2.72	2.19	1.62	0.03	1.31	0.72	0.36	Sensitive	TRUE
ACP2	0.05	3.03	2.61	2.00	0.02	0.60	0.26	0.03	Sensitive	TRUE
SLC9A7	0.05	5.06	6.39	5.53	0.00	-2.23	-1.59	-1.08	Sensitive	TRUE
ICAM2	0.05	4.06	4.30	3.56	0.03	-0.17	-0.06	0.00	Sensitive	TRUE
SLC39A14	0.04	6.85	11.13	10.08	0.00	-1.49	-0.86	-0.47	Sensitive	TRUE
SLC41A2	0.04	3.23	2.91	2.27	-0.01	-2.89	-2.41	-1.82	Sensitive	TRUE
MFSD2A	0.04	9.06	18.89	17.64	0.00	1.48	0.85	0.47	Sensitive	TRUE
CD86	0.04	7.93	14.65	13.50	0.00	0.46	0.19	0.03	Sensitive	TRUE
TSPAN33	0.04	7.16	12.11	11.03	0.00	-0.08	-0.03	0.00	Sensitive	TRUE
LAYN	0.03	3.99	4.19	3.46	0.00	-0.79	-0.36	-0.11	Sensitive	TRUE
ADCY3	0.03	2.64	2.08	1.52	-0.01	-2.40	-1.79	-1.26	Sensitive	TRUE
NCSTN	0.03	2.84	2.35	1.77	0.02	0.78	0.36	0.11	Sensitive	TRUE
IL6R	0.03	7.54	13.33	12.22	0.00	1.53	0.90	0.50	Sensitive	TRUE
PDCD1	0.03	4.56	5.30	4.50	0.02	2.44	1.83	1.30	Sensitive	TRUE
NTRK1	0.03	5.70	7.92	6.99	0.01	1.48	0.86	0.47	Sensitive	TRUE
PVR	0.03	3.62	3.53	2.84	0.00	-4.22	-4.61	-3.85	Sensitive	TRUE
CD68	0.03	4.69	5.57	4.75	0.01	1.17	0.62	0.28	Sensitive	TRUE
FKRP	0.03	2.65	2.09	1.53	0.01	-1.25	-0.67	-0.32	Sensitive	TRUE
P2RY11	0.03	3.11	2.73	2.11	0.01	0.78	0.36	0.11	Sensitive	TRUE
SEMA7A	0.03	5.45	7.30	6.40	0.01	1.47	0.85	0.46	Sensitive	TRUE
SLC2A6	0.03	8.20	15.62	14.45	0.00	2.31	1.69	1.17	Sensitive	TRUE
SLC43A2	0.02	6.56	10.27	9.25	0.01	2.11	1.46	0.98	Sensitive	TRUE
SLC30A1	0.02	3.62	3.52	2.84	0.01	-0.66	-0.29	-0.06	Sensitive	TRUE
QSOX2	0.02	2.61	2.04	1.49	0.00	-0.65	-0.29	-0.05	Sensitive	TRUE
GPR84	0.02	7.38	12.79	11.70	0.01	2.36	1.74	1.22	Sensitive	TRUE
IL1RL1	0.02	4.36	4.88	4.10	0.00	-0.24	-0.09	0.00	Sensitive	TRUE
NLGN4Y	0.02	6.00	8.72	7.76	0.00	0.76	0.35	0.10	Sensitive	TRUE
KCNK5	0.02	2.77	2.26	1.68	0.00	-1.16	-0.61	-0.27	Sensitive	TRUE
TFPI	0.02	5.12	6.51	5.65	0.01	0.67	0.30	0.06	Sensitive	TRUE
ART3	0.02	6.00	8.72	7.76	0.00	0.69	0.31	0.07	Sensitive	TRUE
SLC37A1	0.02	2.53	1.94	1.39	0.01	0.55	0.24	0.03	Sensitive	TRUE
SLC43A3	0.02	6.13	9.05	8.08	0.00	-2.79	-2.28	-1.70	Sensitive	TRUE
MYOF	0.02	4.78	5.75	4.93	0.00	-0.26	-0.10	0.00	Sensitive	TRUE
MRC2	0.02	3.71	3.68	2.98	0.00	-0.07	-0.03	0.00	Sensitive	TRUE
SSTR2	0.01	3.98	4.16	3.43	0.00	2.03	1.37	0.90	Sensitive	TRUE
GALR2	0.01	5.12	6.50	5.64	0.00	1.32	0.73	0.36	Sensitive	TRUE
HYAL2	0.01	3.94	4.09	3.37	-0.01	-3.42	-3.20	-2.54	Sensitive	TRUE
ADAM28	0.01	3.89	3.99	3.27	0.00	-0.57	-0.24	-0.03	Sensitive	TRUE
SCARB1	0.01	3.11	2.73	2.11	0.00	-0.86	-0.41	-0.15	Sensitive	TRUE

FLVCR2	0.01	3.90	4.02	3.30	0.00	1.70	1.05	0.63	Sensitive	TRUE
ITGB8	0.01	4.18	4.53	3.77	0.00	-0.04	-0.01	0.00	Sensitive	TRUE
ATP1A4	0.01	4.81	5.83	5.00	0.01	2.12	1.47	0.98	Sensitive	TRUE
IL13RA1	0.01	4.16	4.50	3.75	0.00	-1.17	-0.61	-0.28	Sensitive	TRUE
SLC14A1	0.01	3.10	2.72	2.10	0.00	2.36	1.74	1.22	Sensitive	TRUE
JAG1	0.01	3.93	4.07	3.34	0.00	0.27	0.11	0.00	Sensitive	TRUE
TIE1	0.01	2.85	2.36	1.77	0.00	1.50	0.87	0.48	Sensitive	TRUE
CD276	0.01	3.26	2.95	2.31	0.00	-2.07	-1.41	-0.93	Sensitive	TRUE
CD200	0.01	3.10	2.71	2.09	0.00	0.08	0.03	0.00	Sensitive	TRUE
SLC46A1	0.01	3.54	3.40	2.72	0.00	0.15	0.06	0.00	Sensitive	TRUE
FCGR1A	0.01	3.08	2.68	2.06	0.00	0.48	0.20	0.03	Sensitive	TRUE
SUCNR1	0.01	4.55	5.27	4.47	0.00	1.23	0.66	0.31	Sensitive	TRUE
SLCO5A1	0.01	3.71	3.68	2.98	0.00	0.69	0.31	0.07	Sensitive	TRUE
ITGA2B	0.01	3.68	3.63	2.94	0.00	1.79	1.13	0.69	Sensitive	TRUE
TENM3	0.01	3.91	4.03	3.31	0.00	1.43	0.81	0.44	Sensitive	TRUE
P2RX1	0.01	3.24	2.92	2.28	0.00	2.05	1.39	0.92	Sensitive	TRUE
SLC15A3	0.00	3.13	2.76	2.14	0.00	0.00	0.00	0.00	Sensitive	TRUE
CD36	0.00	2.73	2.20	1.63	0.00	-0.49	-0.20	-0.03	Sensitive	TRUE
DLL3	0.00	3.83	3.89	3.18	0.00	0.20	0.08	0.00	Sensitive	TRUE
SIRPA	0.00	2.94	2.49	1.89	0.00	0.91	0.44	0.17	Sensitive	TRUE
NOTCH4	0.00	3.02	2.60	1.99	0.00	-0.79	-0.37	-0.11	Sensitive	TRUE
CSF3R	0.00	3.44	3.24	2.58	0.00	1.20	0.64	0.30	Sensitive	TRUE
MERTK	0.00	2.62	2.06	1.50	0.00	-0.62	-0.27	-0.04	Sensitive	TRUE
RXFP1	0.00	2.63	2.07	1.51	0.00	-0.74	-0.34	-0.09	Sensitive	TRUE
SSPN	0.00	3.01	2.59	1.98	0.00	1.21	0.65	0.30	Sensitive	TRUE
HCAR3	0.00	2.61	2.04	1.49	0.00	0.00	0.00	0.00	Sensitive	TRUE
DLL4	0.00	3.18	2.84	2.21	0.00	0.53	0.22	0.03	Sensitive	TRUE
SLC22A4	0.00	4.01	4.22	3.49	0.00	1.68	1.04	0.61	Sensitive	TRUE
CADM1	0.00	2.88	2.40	1.80	0.00	0.21	0.08	0.00	Sensitive	TRUE
SLC8A3	0.00	2.99	2.55	1.95	0.00	0.00	0.00	0.00	Sensitive	TRUE
MUC4	0.00	2.80	2.29	1.71	0.00	1.48	0.86	0.47	Sensitive	TRUE
TMPRSS6	0.00	3.13	2.76	2.13	0.00	-0.48	-0.20	-0.03	Sensitive	TRUE
LRRTM2	0.00	2.57	2.00	1.45	0.00	0.98	0.48	0.19	Sensitive	TRUE
SLC5A3	0.06	-0.25	-0.09	0.00	0.28	10.82	26.56	25.15	Resistant	TRUE
TNFRSF14	0.07	1.02	0.52	0.21	0.23	10.87	26.80	25.38	Resistant	TRUE
ITGA1	-0.16	-11.12	-27.99	-26.56	0.21	6.72	10.75	9.71	Resistant	TRUE
PIK3IP1	-0.04	-7.73	-13.98	-12.85	0.17	11.09	27.86	26.43	Resistant	TRUE
SPN	-0.03	-2.16	-1.51	-1.02	0.13	5.94	8.53	7.58	Resistant	TRUE
HLA-C	-0.21	-2.52	-1.93	-1.39	0.10	8.79	17.82	16.59	Resistant	TRUE
CXCR4	-0.07	-4.89	-6.00	-5.16	0.10	3.52	3.36	2.68	Resistant	TRUE
CD44	0.06	0.43	0.18	0.02	0.10	4.20	4.58	3.82	Resistant	TRUE
ITGB7	-0.12	-10.48	-24.96	-23.58	0.09	3.97	4.14	3.41	Resistant	TRUE
P2RY8	-0.13	-11.42	-29.48	-28.02	0.09	3.46	3.27	2.60	Resistant	TRUE
EMP3	-0.44	-12.44	-34.78	-33.22	0.09	6.31	9.57	8.58	Resistant	TRUE
CLN3	-0.04	-3.58	-3.46	-2.78	0.07	4.61	5.40	4.60	Resistant	TRUE
TRPV2	-0.05	-7.06	-11.79	-10.72	0.07	2.64	2.08	1.52	Resistant	TRUE
IL10RA	-0.17	-10.37	-24.45	-23.08	0.07	2.76	2.23	1.66	Resistant	TRUE
ITGAL	-0.01	0.62	0.27	0.04	0.04	2.67	2.12	1.55	Resistant	TRUE
EMB	-0.49	-21.73	-104.02	-101.79	0.04	2.98	2.54	1.94	Resistant	TRUE
TMEM158	-0.01	-5.91	-8.46	-7.51	0.03	4.41	4.98	4.20	Resistant	TRUE
RNF43	0.01	2.30	1.67	1.16	0.02	5.28	6.89	6.01	Resistant	TRUE
HLA-E	-0.19	-8.46	-16.58	-15.38	0.02	2.62	2.05	1.50	Resistant	TRUE
SLC52A1	0.00	1.38	0.77	0.40	0.01	4.63	5.44	4.64	Resistant	TRUE
CD34	0.00	0.68	0.30	0.07	0.01	2.68	2.14	1.57	Resistant	TRUE
TLR3	0.00	1.18	0.62	0.28	0.01	2.48	1.88	1.34	Resistant	TRUE

RHBDL2	0.00	2.41	1.79	1.27	0.00	3.22	2.89	2.25	Resistant	TRUE
DPEP3	0.00	-1.98	-1.33	-0.86	0.00	2.88	2.40	1.81	Resistant	TRUE
CD300LF	0.00	0.00	0.00	0.00	0.00	2.86	2.38	1.79	Resistant	TRUE
EDA	0.00	0.37	0.15	0.00	0.00	2.67	2.12	1.56	Resistant	TRUE
